# Human pluripotent stem cell-derived kidney organoids reveal tubular epithelial pathobiology of heterozygous *HNF1B*-associated dysplastic kidney malformations

**DOI:** 10.1016/j.stemcr.2024.04.011

**Published:** 2024-05-23

**Authors:** Ioannis Bantounas, Kirsty M. Rooney, Filipa M. Lopes, Faris Tengku, Steven Woods, Leo A.H. Zeef, I-Hsuan Lin, Shweta Y. Kuba, Nicola Bates, Sandra Hummelgaard, Katherine A. Hillman, Silvia Cereghini, Adrian S. Woolf, Susan J. Kimber

**Affiliations:** 1 Division of Cell Matrix Biology and Regenerative Medicine, Faculty of Biology, Medicine and Health, University of Manchester, and the Manchester Academic Health Science Centre, Manchester, UK; 2 Bioinformatics Core Facility, University of Manchester, Manchester, UK; 3 Department of Biomedicine, Aarhus University, Denmark; 4 Manchester Institute of Nephrology and Transplantation, Manchester University NHS Foundation Trust, Manchester, UK; 5 Sorbonne Université, CNRS, Institut de Biologie Paris Seine, Laboratorial de Biologie du Développement, IBPS, UMR7622, F-75005 Paris, France; 6 Royal Manchester Children’s Hospital, Manchester University NHS Foundation Trust, Manchester, UK

**Keywords:** HNF1B, kidney disease, kidney RNA-seq, proximal tubule, pluripotent stem cells, organoid, CRISPR, glutamate receptors, GRIK3, cAMP

## Abstract

*Hepatocyte nuclear factor 1B* (*HNF1B*) encodes a transcription factor expressed in developing human kidney epithelia. Heterozygous *HNF1B* mutations are the commonest monogenic cause of dysplastic kidney malformations (DKMs). To understand their pathobiology, we generated heterozygous *HNF1B* mutant kidney organoids from CRISPR-Cas9 gene-edited human embryonic stem cells (ESCs) and induced pluripotent stem cells (iPSCs) reprogrammed from a family with *HNF1B*-associated DKMs. Mutant organoids contained enlarged malformed tubules displaying deregulated cell turnover. Numerous genes implicated in Mendelian kidney tubulopathies were downregulated, and mutant tubules resisted the cyclic AMP (cAMP)-mediated dilatation seen in controls. Bulk and single-cell RNA sequencing (scRNA-seq) analyses indicated abnormal *Wingless/Integrated* (WNT), calcium, and glutamatergic pathways, the latter hitherto unstudied in developing kidneys. Glutamate ionotropic receptor kainate type subunit 3 *(GRIK3)* was upregulated in malformed mutant nephron tubules and prominent in *HNF1B* mutant fetal human dysplastic kidney epithelia. These results reveal morphological, molecular, and physiological roles for HNF1B in human kidney tubule differentiation and morphogenesis illuminating the developmental origin of mutant-*HNF1B*-causing kidney disease.

## Highlights

• Human ESC- and iPSC- derived organoids modeled heterozygous *HNF1B* malformed kidneys
• Mutant organoids had malformed tubules and glomeruli with gene dysregulation
• Bulk and scRNA-seq together revealed a unique mal-differentiated tubule population
• GRIK3 was high in dysplastic tubules in *HNF1B* mutant native kidneys and organoids

## Graphical abstract

**Figure:**
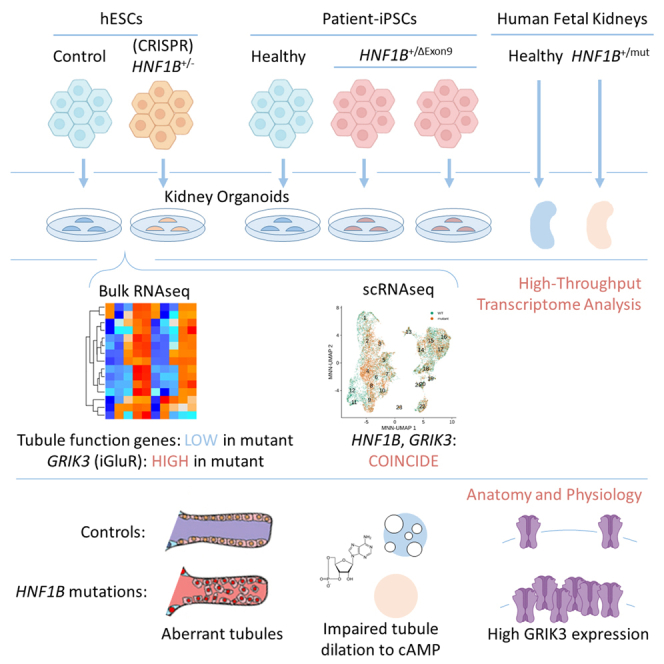

## Teaser

Bantounas and colleagues created an organoid model of +/−*HNF1B*-kidney disease by differentiation of CRISPR-edited hESCs or *HNF1B*-patient iPSCs. Mutant organoids had tubules with anatomical and physiological aberrations, and abnormal glomeruli. Bulk- and scRNA-seq identified dysregulation of key developmental gene pathways involved in inherited kidney disease and altered cell-type composition suggesting a novel role for GRIK3 in kidney development and disease.

## Introduction

Mammalian kidney development is a complex process (Jafree et al., 2019; Wilson and Little, 2021; Woolf, 2019). The human metanephric kidney initiates at 5 weeks gestation (Woolf, 2019) when the metanephric mesenchyme (MM) is penetrated by the ureteric bud (UB). MM/UB crosstalk results in MM undergoing mesenchymal-to-epithelial transition to generate primitive nephrons, each differentiating into a glomerulus, a proximal tubule (PT), and the distal nephron tubule (DT), including the loop of Henle and the distal convoluted tubule (McMahon, 2016). Waves of nephrogenesis forming glomeruli and nephron tubules occur between antenatal weeks 7 and 34, and the UB arborizes into collecting ducts (CDs), each fusing with a DT. Stromal fibroblast-like cells (Wilson and Little, 2021) and blood and lymphatic endothelia (Jafree et al., 2019) are found between developing tubules. Capillaries invade glomerular podocyte tufts delivering blood for filtration, and tubules modify the ultrafiltrate to make definitive urine. Dysplastic kidney malformations (DKMs) are major causes of kidney failure in children and young adults (Kohl et al., 2022). Ultrasonography detects echo-bright organs that, lacking cortical-medullary distinction (Kohl et al., 2022), manifest aberrant nephron morphogenesis and deregulated cell turnover (Winyard et al., 1996a, 1996b).

The commonest genetic DKMs are due to heterozygous mutations of *hepatocyte nuclear factor 1B* (*HNF1B*) (Adalat et al., 2009, 2019). *HNF1B* encodes a homeodomain transcription factor that dimerizes with itself or *HNF1A* (Clissold et al., 2015). *HNF1B*-associated kidney disease can vary in severity, without clear genotype-phenotype correlations (Lim et al., 2020). Some fetuses undergo termination (Haumaitre et al., 2006). Others survive but postnatally exhibit urinary electrolyte wasting, suggesting that HNF1B is required for tubular functional differentiation (Adalat et al., 2019, 2009). *HNF1B* is expressed in developing and mature human nephron epithelia and CDs (Haumaitre et al., 2006; Kolatsi-Joannou et al., 2001) but not in the MM, stroma, or mature podocytes. *HNF1B*-associated DKMs contain abnormal multi-layered tubules and dysmorphic glomeruli (Haumaitre et al., 2006; Nakayama et al., 2023).

*HNF1B* has been manipulated in kidney somatic cell lines (Chan et al., 2020; Piedrafita et al., 2021), *Xenopus* (Grand et al., 2023), and mice. Cre-mediated deletion of both alleles in mouse metanephroi deregulates expression of genes including those implicated in WNT signaling and polycystic kidney disease (PKD) (Desgrange et al., 2017; Fiorentino et al., 2020; Gresh et al., 2004; Lokmane et al., 2010). Postnatal biallelic deletion perturbs mouse kidney mitochondrial respiration (Casemayou et al., 2017), enhances fibrosis (Chan et al., 2018), and impairs regeneration (Verdeguer et al., 2010). Germline homozygous mutant mice are early embryonic lethal, and humans with biallelic germline mutations have not been described. Thus, the relevance of biallelic models to understand human heterozygous *HNF1B*-associated DKMs is uncertain. Recently, however, aberrant kidney tubules were described in mice carrying a heterozygous intron 2 splice donor site germline *Hnf1b* mutation (Niborski et al., 2021). Nevertheless, kidney gene expression patterns are not always identical in humans and mice (Lindstrom et al., 2018), so new human experimental models are needed.

Kidney organoids from human pluripotent stem cells (hPSCs) are being used to understand normal and abnormal kidney development (Bantounas et al., 2018; Rooney et al., 2021; Taguchi et al., 2014; Takasato et al., 2015; Woolf, 2019). We hypothesized that such hPSC-derived organoids would model human *HNF1B-*associated DKMs. We therefore undertook morphological, functional, and molecular analyses of heterozygous *HNF1B* mutant organoids derived from CRISPR-Cas9 gene-edited wild-type human embryonic stem cells (hESCs) or from human induced pluripotent stem cells (iPSCs) (hiPSCs) reprogrammed from peripheral blood mononuclear cells (PBMNs) of siblings with *HNF1B*-associated DKMs. Mutant organoids displayed malformed nephrons and deregulated cell turnover. Genes implicated in Mendelian tubulopathies were downregulated in mutant organoids which resisted cyclic AMP (cAMP)-mediated tubule dilatation, seen in unaffected controls. Bioinformatic analyses predicted abnormal pathways, including WNT, and glutamatergic signaling, the latter hitherto unstudied in kidney development. Glutamate ionotropic receptor (iGluR) kainate type subunit 3 (GRIK3) was markedly upregulated in mutant organoids and detected in human fetal *HNF1B*-associated DKM tubules. Our results illuminate important roles for HNF1B in human kidney development and identify potentially druggable targets.

## Results

### Generating heterozygous *HNF1B* mutant hESCs

**Figure 1:**
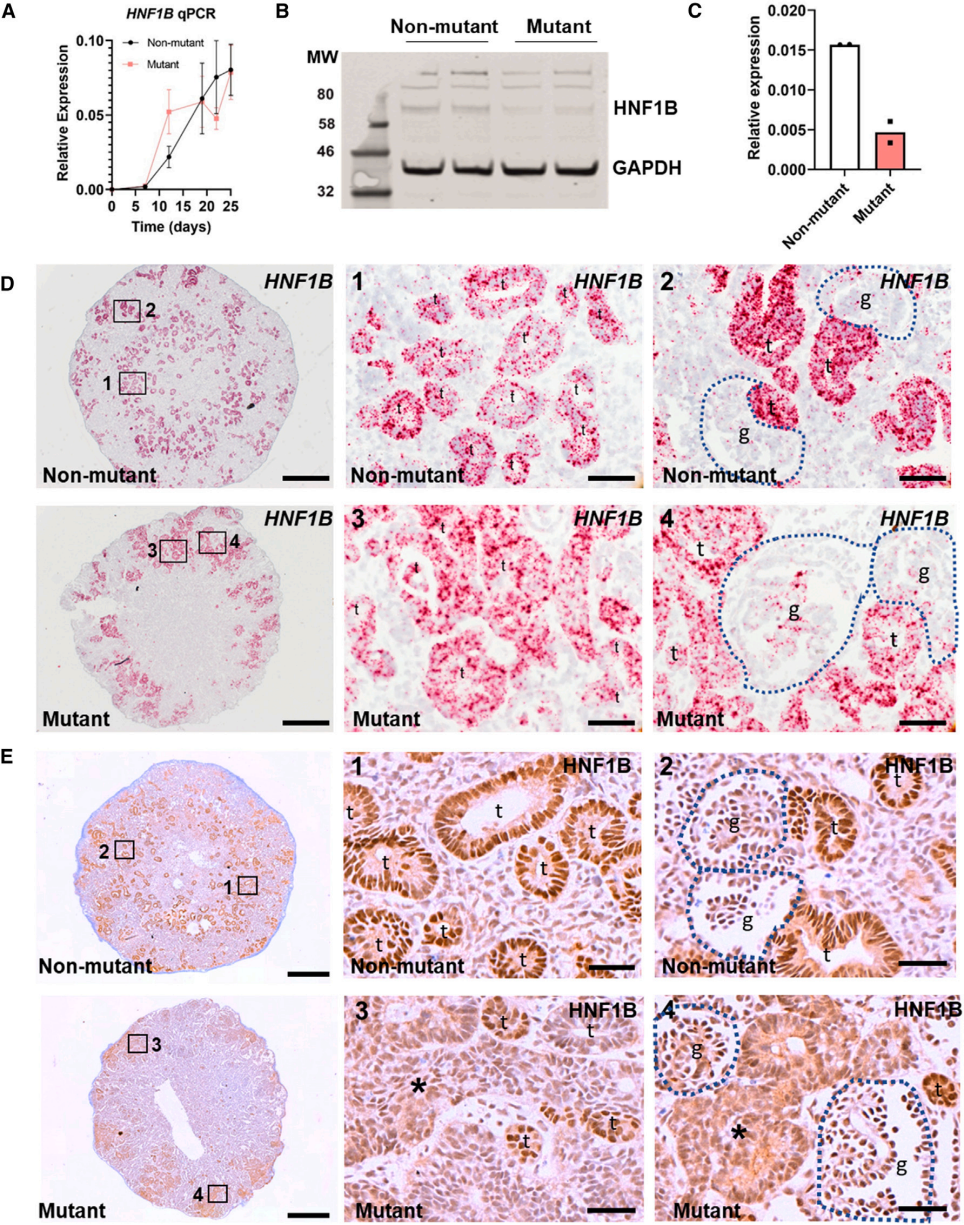
HNF1B in hESC-derived organoids (A) RT-qPCR using *HNF1B* exon 2 primers showed similar upregulation in *mutant* and isogenic *non-mutant* lines over 25 days; bars denote standard error of the mean (SEM). (B) Western blot of day 25 organoids (two organoid pools/genotype) detected HNF1B doublets at 63 kDa. (C) Decreased HNF1B/GAPDH values in mutant organoids (mean and individual values shown). (D) BaseScope ISH *HNF1B* (red dots) with nuclei counterstained blue with hematoxylin. Left-hand frames show low power overviews with enlarged areas in boxes 1–4. Non-mutant tubules (*t* in 1) expressed *HNF1B*, but signal was scarce in glomeruli (*g* in 2). In mutants, *HNF1B* was expressed in bulky, aberrant tubules (*t* in 3) and in tufts of aberrant-looking glomeruli (*g* in 4). (E) HNF1B immunostaining (brown) in day-25 organoids, with hematoxylin counterstain. Left-hand frames; overviews with boxes 1–4 enlarged in the other frames. HNF1B was detected in nuclei of wild-type tubules (*t* in 1 and 2). In mutants, HNF1B was in small-caliber tubules (*t* in 3 and 4), but signals were attenuated and diffuse in bulky, aberrant tubules (*asterisks* in 3 and 4). Bars: (D) 200 μM (overview) and 20 μM (enlargements); and (E) 500 μM (overview) and 40 μM (enlargements).

CRISPR-Cas9 gene editing was used to mutate *HNF1B* in MAN13 hESCs (hPSCreg-ID: UMANe002-A) and create the IBM13-19 heterozygous line (hPSCreg-ID: UMANe002-A-4; hereafter called “mutant”) (Figure S1A), which harbors a frameshift in exon 1 (Figure S1B), resulting in a premature stop codon 51 nucleotides downstream and a protein lacking the DNA binding and transactivation domains. A non-mutated clone (IBM13-08; hPSCreg-ID: UMANe002-A-5) was used as isogenic control line (Figure S1B). Using a 2D kidney differentiation protocol (Bantounas et al., 2018, 2021), we observed an attenuated immunohistochemical HNF1B signal in the mutant with an antibody against a 111 amino acid epitope that would be disrupted by the mutation (Figure S1C). We next generated mutant and unaffected kidney organoids (Bantounas et al., 2018, 2021; Takasato et al., 2015) (Figure S1D) of similar size (Figures S1E and S1F), but phase contrast suggested larger internal structures in mutant organoids (Figure S1G). Quantitative reverse-transcription PCR (RT-qPCR) using primers that would detect both non-mutant and mutant *HNF1B* mRNAs showed similar non-mutant and mutant levels (Figure 1A), while HNF1B protein was, as expected, significantly decreased in the mutant (Figures 1B and 1C). RNA *in situ* hybridization (ISH) with a probe detecting non-mutant and mutant HNF1B transcripts showed tubular expression, including in aberrant mutant tubules (Figure 1D). HNF1B immunohistochemistry followed this pattern, but HNF1B was attenuated in the mutant (Figure 1E).

### Histology and functionality of hESC-derived organoids

**Figure 2:**
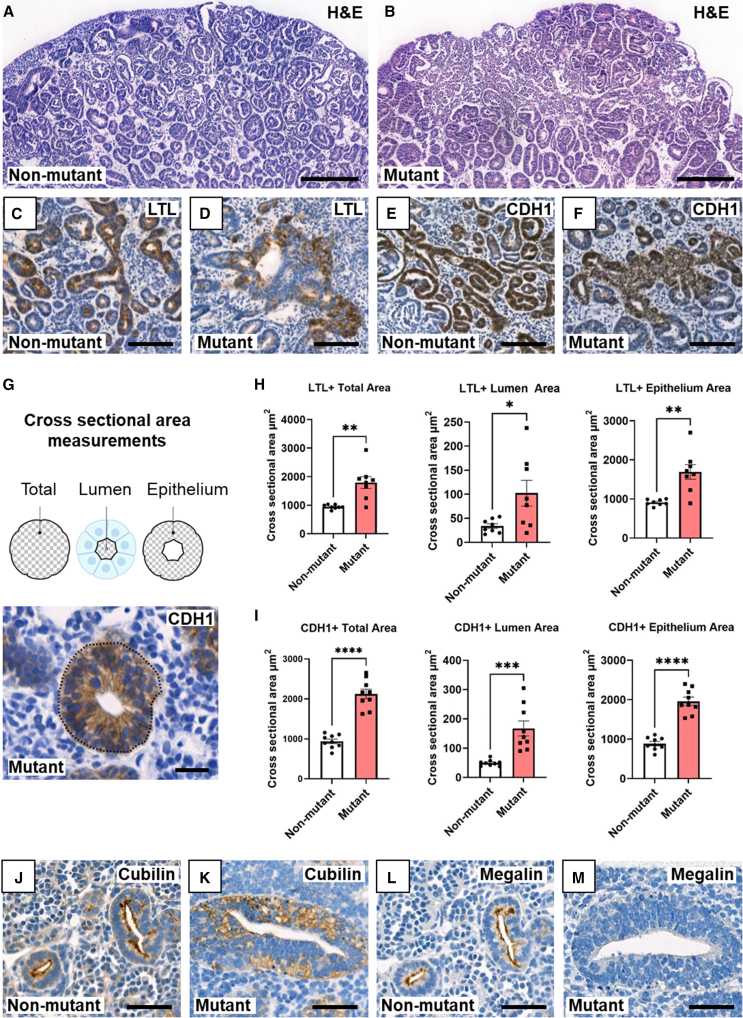
Aberrant tubules in *HNF1B* mutant organoids (A) Non-mutant and (B) mutant organoids counterstained with hematoxylin and eosin. Internal structures appeared larger in mutants. (C) Non-mutant and (D) mutant organoids stained with LTL (brown) with hematoxylin counterstain, showing slender LTL+ non-mutant tubules and bulky mutant tubules with patchy staining. (E and F) (E) Non-mutant and (F) mutant organoids immunostained for CDH1 (brown) with hematoxylin counterstain, showing slender CDH1+ wild-type tubules and bulky mutant tubules with patchy staining. (G) Above: cartoon showing the total, lumen, and epithelium areas measured from perpendicularly cross-sectioned tubules. Below: example tubule immunostained (brown) for CDH1 in mutant. (H) Area of LTL+ profiles. (I) Areas of CDH1+ profiles (in H and I: mean ± SEM; *n* = 9 organoids from three independent differentiation experiments; ^∗^*p* < 0.05, ^∗∗^*p* < 0.005, ^∗∗∗^*p* < 0.0005, ^∗∗∗∗^*p* < 0.00005, t test). (J and K) Cubilin immunostaining (brown): apical pattern in non-mutant tubules but a diffuse pattern in mutants. (L and M) Megalin immunostaining: apical pattern in non-mutant tubules (L) but not detected in mutant tubules (M). Bars: (A and B) 200 μM; (C–F) 100 μM; (G) 20 μM; and (J–M) 50 μM.

**Figure 3:**
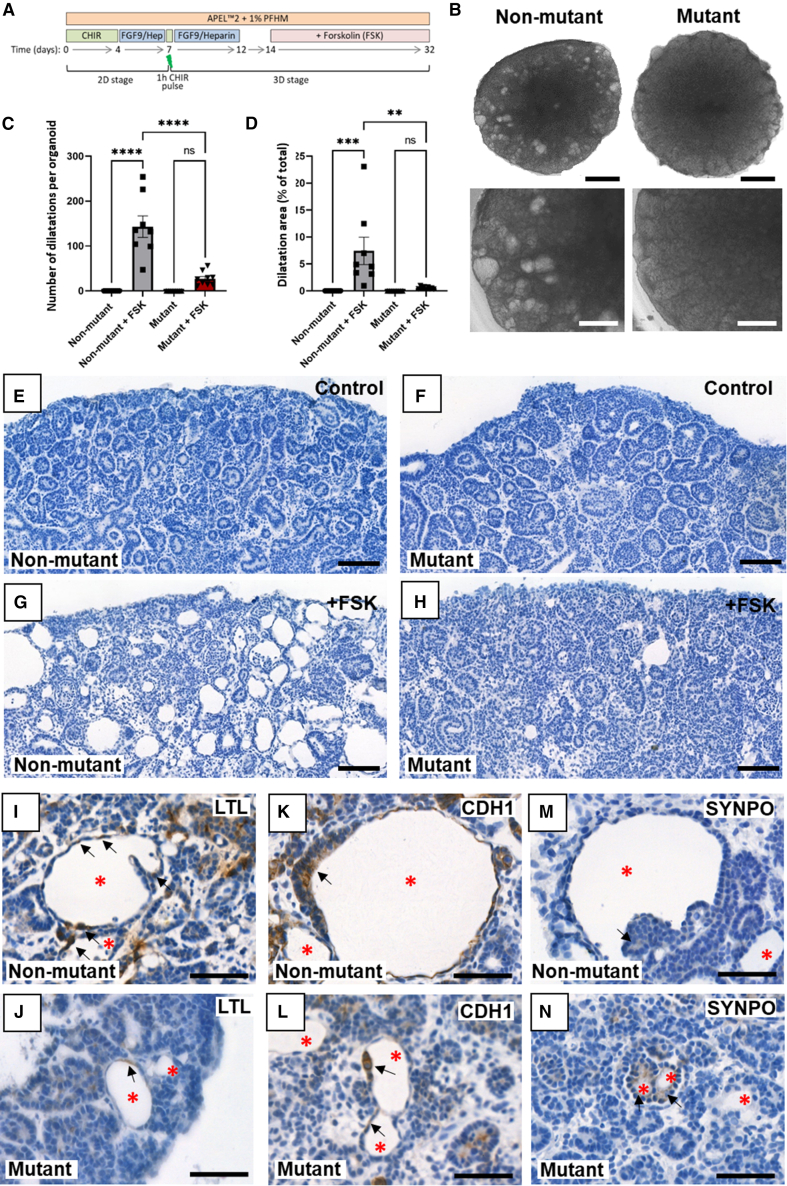
Deficient cAMP-induced lumen dilatation in *HNF1B* mutant organoids (A) Organoids were exposed to forskolin (FSK) between 14 and 32 days. (B) Phase contrast images at day 32 showed that FSK had induced numerous dilated structures in non-mutant organoids but few in mutants. (C) Numbers of dilatations per organoid on histology. (D) Quantification of total dilated percentage area per organoid (C and D: mean ± SEM; *n* = 9 organoids across 3 independent experiments; ^∗∗∗∗^*p* < 0.00005, ^∗∗∗^*p* < 0.0005, ^∗∗^*p* < 0.005, one-way ANOVA with multiple comparisons). (E–H) Hematoxylin stained sections of non-mutant and mutant organoids, without (control) or with added FSK (+FSK). (I–N) Organoid sections reacted with LTL or immunoprobed for CDH1 or SYNPO and counterstained with haematoxylin. Red asterisks indicate dilated structures; black arrows indicate associated cells. Bars: (B) 1 mm (upper panels) and 500 μM (lower panels); (E–H) 200 μM; and (I–N) 50 μM.

Analysis of the organoids revealed bulkier tubules (Figures 2A and 2B) in mutant organoids. In control organoids *Lotus tetragonolobus* lectin (LTL), a PT marker (Kishi et al., 2019), bound a subset of slender tubules in a uniform manner (Figure 2C), whereas mutant dysmorphic tubules exhibited patchy staining (Figure 2D). In native human kidneys, DTs and CDs are rich in CDH1 (Nouwen et al., 1993). In control organoids, a subset of tubules was CDH1+ (Figure 2E), and mutant dysmorphic tubules showed patchy immunostaining (Figure 2F). For both LTL+ and CDH1+ tubules, the cross-sectional areas of mutant tubules were significantly larger than those of wild-type tubules (Figures 2G–2I). *In vivo*, Megalin and Cubilin form a receptor complex on the apical (luminal) plasma membrane of PTs (Nielsen et al., 2016). Slender control organoid tubules displayed this apical pattern (Figures 2J and 2L), but bulky mutant tubules showed predominantly cellular Cubilin immunostaining (Figure 2K), while Megalin was absent (Figure 2M). Synaptopodin+ (a podocyte, actin-associated protein) glomeruli appeared larger with less compact podocyte tufts in the mutant (Figures S1H, and S1I). Control and mutant organoids contained PECAM1+ endothelia between tubules, but neither featured capillaries within glomerular tufts (Figures S1J and S1K). We measured bromodeoxyuridine (BrdU) incorporation (proliferation) and activated Caspase-3 immunostaining (apoptosis) (Figures S1L–S1Q). BrdU+ tubule nuclei and apoptotic figures were significantly increased across the whole organoid in mutants. Forskolin (FSK) is an adenylate cyclase activator that increases intracellular 3′,5′-cAMP. When wild-type metanephroi are exposed to FSK in organ culture, nephron lumina dilate, reflecting fluid transport and tubule functionality (Anders et al., 2013). Adding FSK to organoids (Figure 3A) generated numerous translucent areas in controls, whereas few were evident in mutants (Figure 3B). Both the numbers and the area occupied by these dilatations were significantly less in mutants (Figures 3C–3H). Probing with LTL or for CDH1 or Synaptopodin (Figures 3I–3N) suggested that dilatations in non-mutant organoids affected PTs, DTs, and glomeruli. The smaller dilated structures in mutants were harder to categorize, although some affected glomerular tufts (Figure 3N).

### Organoid differentiation from *HNF1B* mutant patient-derived hiPSCs

To determine whether the phenotype of CRISPR-Cas9-mutated hESC-derived organoids was representative of *HNF1B*-associated kidney disease, we evaluated organoids generated from hiPSCs derived from a family carrying a heterozygous deletion of exon 9 (*HNF1B*^+/ΔExon9^) (Figures S2A–S2C). HiPSCs from two brothers with DKMs (TF171A; hPSCreg-ID: UMANCi003-A and TF172D; hPSCreg-ID: UMANCi002-A) and their unaffected *HNF1B*^+/+^ mother (TF173B; hPSCreg-ID: UMANCi001-A) were generated, and the mutation was confirmed by genomic qPCR (Figure S2D). *HNF1B* transcripts, assessed using exon 2 primers, were similar in control and mutant organoids (Figure S2E), but, using mutation-specific (exon 9) primers, transcripts were lower in mutant organoids (Figure S2E). Similarly to the mutant hESC-derived organoids, *HNF1B*^+/ΔExon9^ organoids exhibited dysmorphic tubules with multi-layered epithelia and bulky glomeruli (Figure S2F) and were unable to form cysts after 8-Br-cAMP (Figures S2G–S2I).

### Overview of hESC-derived organoid transcriptomes

For model validity, we compared bulk RNA sequencing (RNA-seq) profiles of differentiating control hESCs with human kidneys at 10–12 weeks gestation when they contain a nephrogenic cortex of MM and branching UB tips, with deeper maturing nephrons, CDs, and stromal cells. Principal-component analysis (PCA) showed that organoid profiles approached those of native fetal kidneys as differentiation progressed (Figure S3A), with key glomerular and PT marker genes, but fewer DT and CD transcripts (Figures S3B–S3G).

Moreover, the transcriptional profiles of control and mutant organoids had similar temporal progression (Figure S3J) during differentiation. When the levels of *HNF1B* rose markedly at day 12 (Figure S3K), the number of significantly differentially expressed genes (DEGs) also increased, up to 631 on day 25 (Figures S3L and S3M). Among these were genes containing the canonical *HNF1B* binding motif upstream of their transcription start site (Figure S3N-RI). Gene ontology (GO) enrichment analysis and Kyoto Encyclopedia of Genes and Genomes (KEGG) pathway analysis, comparing mutant and isogenic control organoids (Figure S4 and Table S4), showed that calcium ion binding, cadherin binding, collagen containing extracellular matrix, and integral component of plasma membrane were dysregulated, along with WNT signaling, a pathway essential for kidney development (with *WNT5A*, *WNT10B*, *FZD4*, and *DKK1* among DEGs).

### Characteristic kidney transcripts in *HNF1B* mutant hESC-derived organoids

**Figure 4:**
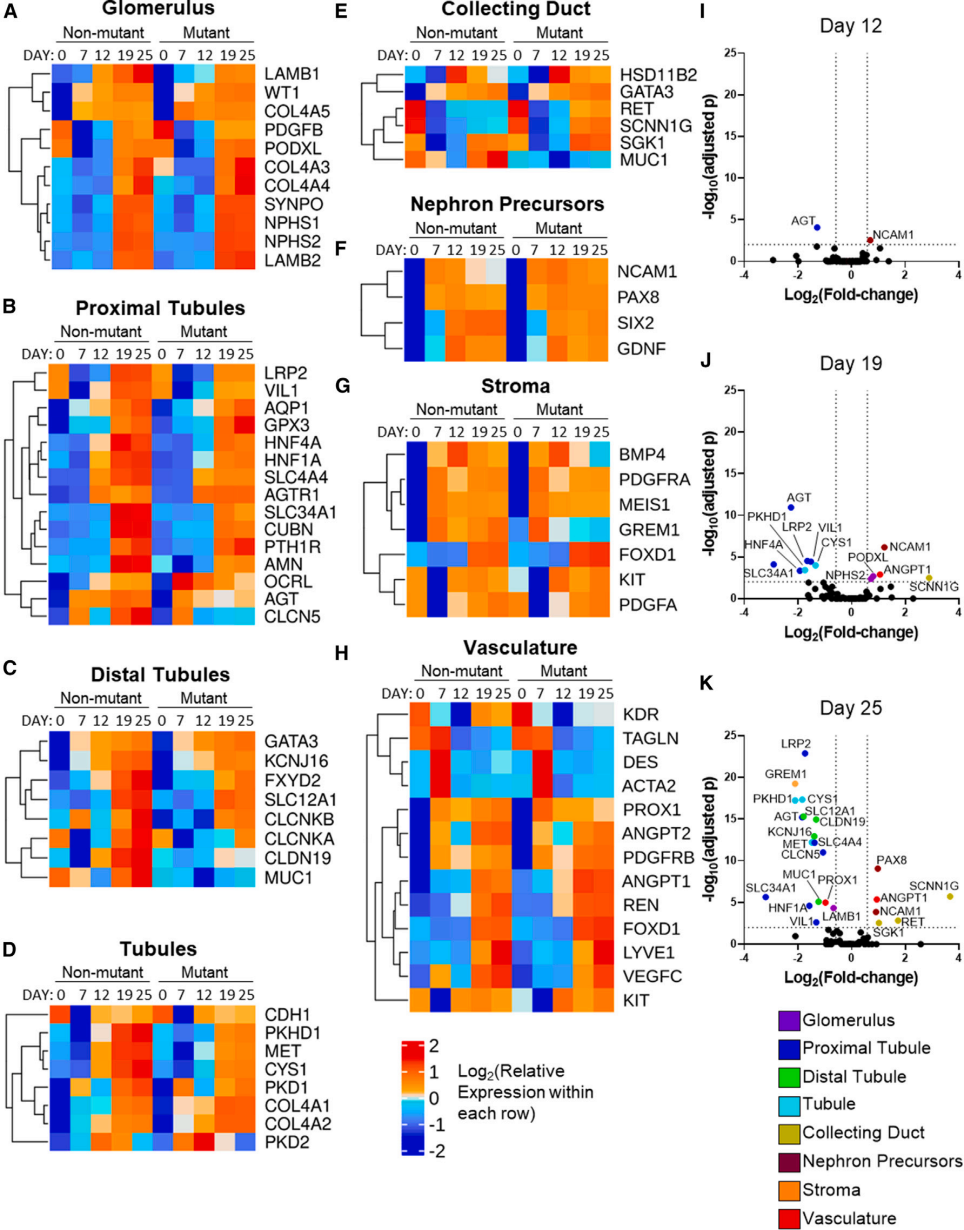
Profiles of established kidney transcripts in hESC-derived organoids (A–H) Heatmaps through the differentiation protocol, with days 12, 19, and 25 being the organoid phase. Each element in the heatmaps represents the mean of three independent differentiation experiments. Genes were included if their average read count >50 on at least one day of organoid differentiation. (I–K) Volcano plots showing significantly deregulated transcripts at days 12 (I), 19 (J), and 25 (K) with cut-offs of a log_2_(fold change) of 0.5 and log_10_(p-adjusted) significance value of 2 with expected lineage color coded; key below.

We examined organoids for cell type-specific or cell type-enriched transcripts in native kidneys. Heatmaps in Figures 4A–4H show transcripts detectable in control and mutant organoids during differentiation (further validating the organoid model), while genes differentially expressed in the mutant are shown in volcano plots (Figures 4I–4K). Strikingly, numerous genes differentially expressed between mutant and non-mutant are implicated in Mendelian kidney diseases (especially tubulopathies), including various channels/transporters (Table S5). Of those, *HNF1A*, a transcription factor binding partner of HNF1B causing PT dysfunction when mutated in mice, was significantly reduced in the mutant, and ISH experiments (Figure S5) detected transcripts in control organoid tubules but a lower signal in mutants.

Additionally, in the glomerulus, basement membrane gene *LAMB1* was significantly lower in day-25 mutants, while podocyte gene *PODXL* was up at day 19 (Figures 4J and 4K). In PTs (additional to genes in Table S5), *VIL1*, encoding brush border Villin, was significantly lower in mutants (Figures 4J and 4K); DT and CD gene *SGK1* (serum/glucocorticoid-regulated kinase 1) and RET (in branching UB branch tips) were upregulated in mutants (Figures 4J and 4K). Genes associated broadly with tubule epithelia were downregulated in mutants (Figures 4I–4K) including *MET* (HGF receptor), autosomal recessive PKD-associated gene *CYS1* (Cystin), and *PKHD1* (Fibrocystin) (Table S5). Considering MM and primitive nephron-expressed transcripts (Figure 4F), *NCAM1* and *PAX8* were higher in mutants (Figures 4I–4K), while *GREM1*, a BMP4-antagonist, was lower (Figure 4K). Among transcripts implicated in differentiation of endothelia and vascular smooth muscle cells (SMCs), *ANGPT1* was significantly higher in mutant organoids, while *PROX1* was lower (Figures 4J and 4K).

### scRNA-seq analysis reveals aberrant cell populations in nephrons of mutant organoids

**Figure 5:**
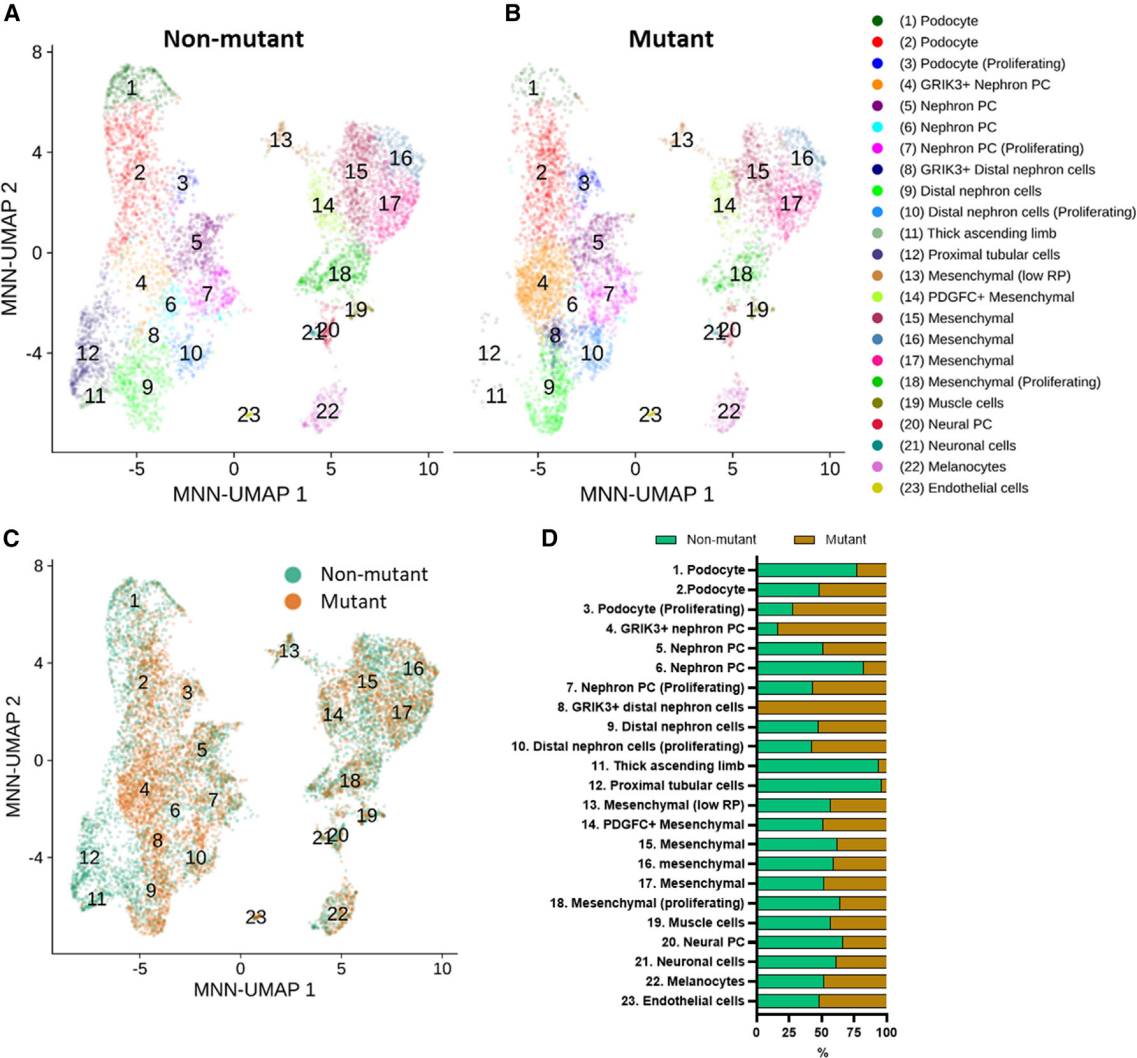
Single-cell RNA-seq comparison of non-mutant and mutant organoids (A and B) MNN-corrected UMAP of cells in non-mutant (A) and mutant (B) organoids, with cell clusters numbered and highlighted in distinct colors. (C) MNN-corrected UMAP of non-mutant and mutant cells in the same space, highlighted in different colors. (D) The percentage of non-mutant and mutant cells in each cluster.

To better understand the molecular and cellular basis of the developmental aberrations, we performed single-cell RNA-seq (scRNA-seq) comparing day-25 non-mutant and mutant organoids, analyzing 6,168 non-mutant and 5,783 mutant cells. Clustering analysis identified 23 distinct cell populations (Figures 5A and 5B). These were annotated by significant known key marker gene expression in each cluster (Table S6). Mutant organoids almost completely lacked molecularly typical PT and thick ascending limb loop of Henle populations (clusters 12 and 11 in Figures 5A–5D). This was consistent with the mutant organoid downregulation of transcripts normally expressed by these tubules, revealed in the bulk RNA-seq analysis. Concordant with histological glomerular aberrations, we identified differences in the podocyte populations between non-mutant and mutant organoids (clusters 1, 2, and 3, in Figures 5A–5D). Cluster 1 was extensively depleted of mutant cells while proliferating podocytes (cluster 3) were more abundant in mutant organoids (∼72% mutant cells) (Figure 5D), and gene expression indicated they were in G2M or S phase.

We also observed differences in three clusters representing nephron progenitor cells between non-mutant and mutant organoids (clusters 4, 5, and 6, in Figures 5A and 5B). Cluster 6 comprised mostly (∼82%) non-mutant cells, while cluster 5 contained non-mutant and mutant cells approximately equally (Figure 5D). Strikingly, cluster 4 contained 84% mutant cells and was highly proliferative with 97% of cells in G2M or S phase from their expression.

### Deregulated glutamate receptor and pathway genes in mutant organoids

*GRIK3*, encoding iGluR kainate type subunit 3, was among the ten most deregulated transcripts in mutants in the bulk RNA-seq. This prompted us to examine iGluR expression (known for its role in neurotransmission and neuronal plasticity (Hansen et al., 2021) in human kidney. In addition to *GRIK3*, several iGluR subunits from all receptor subfamilies (N-methyl-D-Aspartate [NMDA], α-amino-3-hydroxy-5-methyl-4-isoxazole-propionic acid [AMPA], Kainate, Delta) were expressed in fetal human kidneys and organoids (Figure S6A).

**Figure 6:**
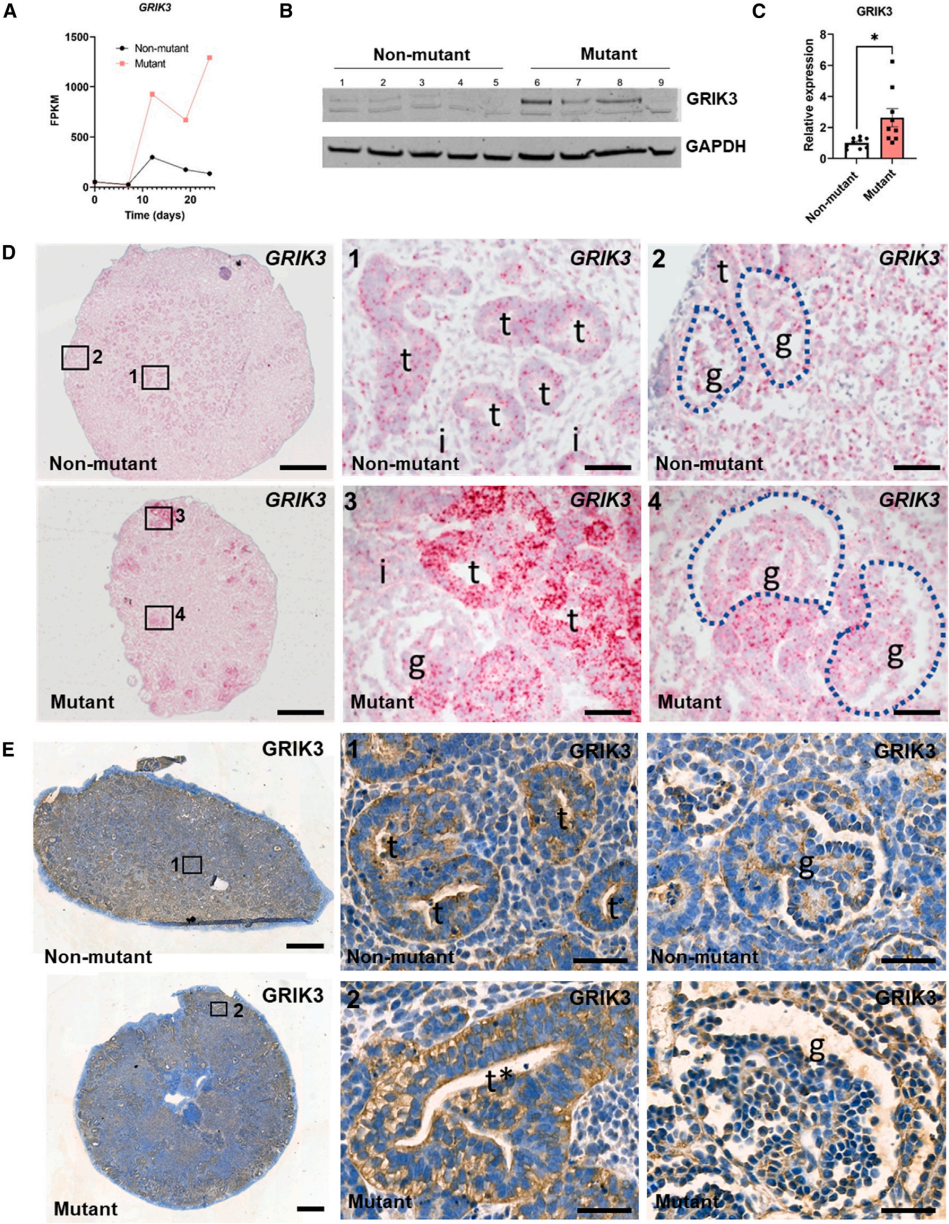
GRIK3 in kidney organoids (A) RNA-seq average read counts of *GRIK3* during differentiation of non-mutant and *HNF1B* mutant hESCs, showing increased levels in mutant organoids (days 12, 19, and 25). (B) GRIK3 western blot (5 non-mutant and 4 mutant samples). (C) Quantification of B confirmed increased GRIK3/GAPDH in mutant organoids (mean ± SEM; *n* = 9, across four independent differentiation experiments; ^∗^*p* < 0.05, t test). (D) BaseScope for *GRIK3*, signal-red dots; nuclei counterstained with hematoxylin. Left-hand images, low power overviews of day 25 organoids; other frames show high power images (1–4). In non-mutant organoids *GRIK3* was expressed in tubules (*t*) with sparser signals in interstitial cells (*i* in 1) and glomeruli (*g* in 2). In mutant organoids, *GRIK3* was highly expressed in large dysmorphic tubules (*t* in 3), with transcripts also in aberrant glomeruli (*g* in 4). (E) GRIK3 immunostaining (brown). Left-hand images: overviews of day 25 organoids; other frames (1 and 2) are high power images. In non-mutant organoids GRIK3 was immunodetected in tubules (*t* in 1). In mutant organoids, GRIK3 was prominent in multi-layered dysplastic tubules (*t* and *asterisk* in 2). A low level of immunostaining was noted in glomeruli (*g*) of both genotypes. Bars: (D) 200 μM (left frames) and 20 μM (other frames); (E) 500 μM (left frames) and 40 μM (other frames).

**Figure 7:**
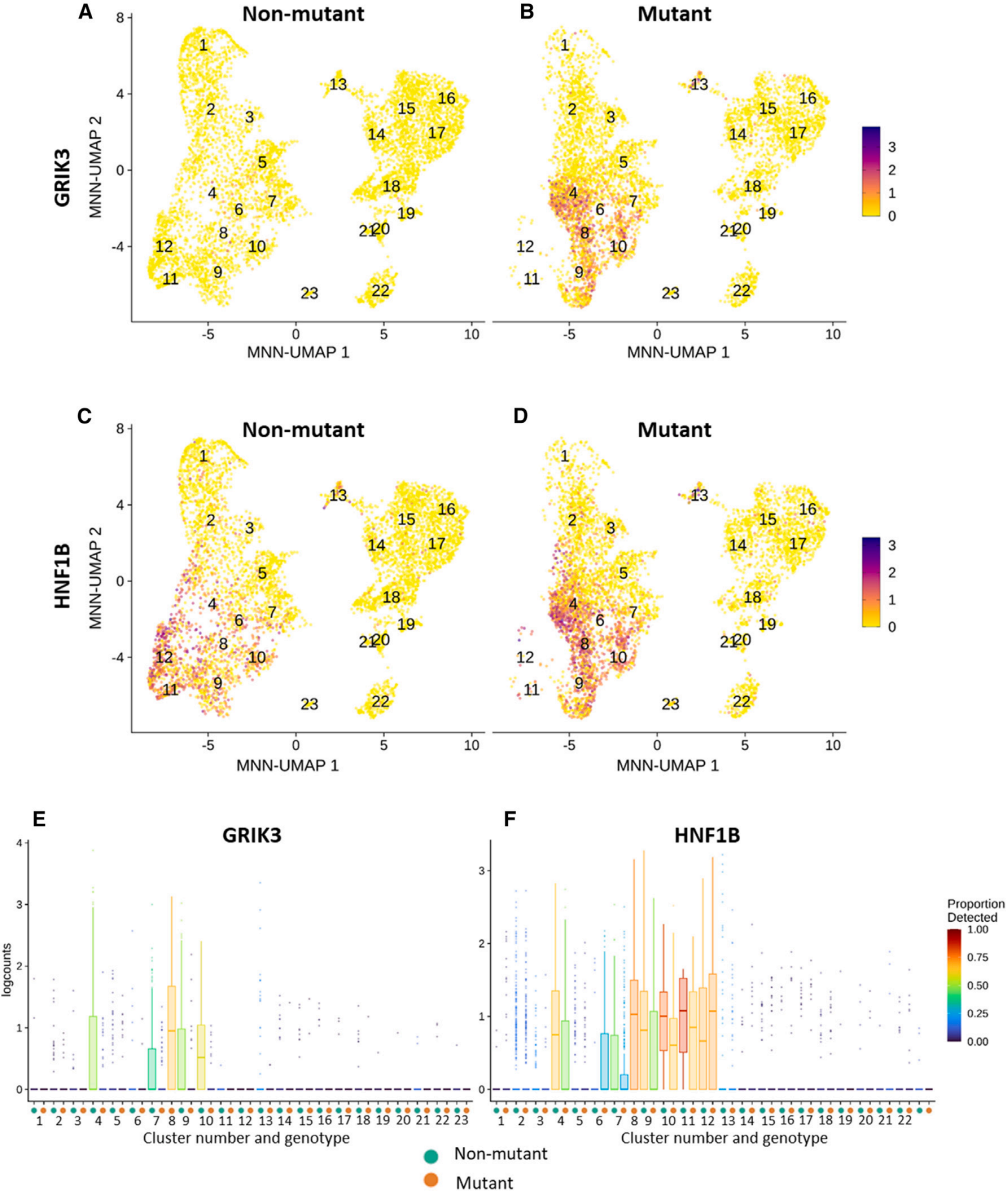
Localization of *GRIK3* and *HNF1B* expression in organoid cells from scRNAseq analyses (A–D) MNN-corrected UMAP of non-mutant and mutant organoid cells, with expression of *HNF1B* and *GRIK3* highlighted, showing extensive co-expression of *GRIK3* (A, B) and *HNF1B* (C, D) in the mutant (B, D). (E and F) Boxplot diagram quantifying the expression of *GRIK3* (E) and *HNF1B* (F) in each cell cluster of non-mutant and mutant organoids. Note that all *GRIK3+* mutant populations also express *HNF1B*.

*GRIK3* levels in human fetal kidneys were similar to those in control organoids (Figure S6A), but *GRIK3* was significantly upregulated on days 12, 19, and 25 in mutant compared with control organoids (Figure S6B). On the final day of culture, *GRIK3* expression was 10 times higher (adjusted *p* value (p(adj)) = 1.95 × 10^−31^) in *HNF1B* mutant than control organoids (Figure 6A). Western blotting showed significantly increased GRIK3 protein in mutant organoids (Figures 6B and 6C). In the scRNA-seq, the mutant cells dominating cluster 4 were characterized by high *GRIK3* expression (Figure 5C). Finally, cluster 8 in the scRNA-seq comprised exclusively mutant cells and represented a unique tubular cell type with high *GRIK3* expression (Figure 5C). Indeed, ISH (Figure 6D) of control organoids detected *GRIK3* in tubules and more sparsely in interstitial cells, while *GRIK3* was prominently expressed in mutant dysplastic tubules. Immunohistochemistry for GRIK3 showed a predominantly tubular pattern in both control and mutant organoids (Figure 6E). On third-trimester fetal kidney sections (Figure S6G–S6P) GRIK3 immunostaining was detected in tubules. Some such tubules had apical periodic acid Schiff (PAS) staining, marking them as PTs. In a fetus carrying a heterozygous *HNF1B* mutation, GRIK3 was prominent in dysplastic tubules, some likely PTs (based on apical PAS). *GRIK3* was highly expressed in nephron progenitors in scRNA-seq and distal nephron populations in mutant organoids (clusters 4, 7, 8, 9, and 10), all also expressing *HNF1B*, supporting association between mutant *HNF1B* and *GRIK3* overexpression (Figures 7A and 7B), although we cannot conclude that *GRIK3* is under direct transcriptional control of *HNF1B* (Figures S3N–S3Q).

iGluR subunits of other subfamilies were also expressed in both fetal kidneys and organoids, with *GRIN2B*, *GRIN3A*, and *GRID1* overexpressed in mutant organoids (Figures S6A–S6E). KEGG enrichment analysis indicated that “glutamatergic synapse signaling” and “calcium signaling” pathways differed between mutant and control organoids (Figure S7, and Table S4), so we examined downstream members of the iGluR signaling pathway: In the mutant, we identified early (day-12) overexpression of glutamate transporters *SLC12A6* and *SLCA11*, followed by day-25 overexpression of Ca^2+^-channel scaffold *HOMER2*, Synaptotagmin-1 (*SYT1*), and *CAMK2A* (Figure S6F). Conversely, the glutamate transporter SLC1A1, the iGluR scaffold *SHANK2*, and the downstream effector of high [Ca^2+^], *PLCB1*, were downregulated in the mutant. Supporting the prevalence of transporter deregulation, GO Cellular Compartment enrichment analysis revealed significant gene enrichment in terms associated with the cell surface (p(adj) < 0.01, Figures S4G–S4I), notably well over 100 genes in the term “integral components of plasma membrane” at day 12, 19, and 25. These included *LRP2*, *PODXL*, *NPHS2*, *PKHD1*, and *MUC1* as well as 11 members of the extensive membrane transporter SLC genes, across a number of solute transport subfamilies, at day 19 and 25.

## Discussion

Our results reveal important roles for HNF1B in human kidney tubule morphogenesis and functional differentiation and suggest druggable targets to ameliorate disease.

### Heterozygous *HNF1B* mutant organoids mimic features of human *HNF1B*-associated DKMs

Human kidneys with DKMs initiate organogenesis, yet their internal organization is deranged (Kohl et al., 2022; Winyard et al., 1996a, 1996b). With regard to *HNF1B*-associated DKMs, histological features include large multi-layered tubules and dysmorphic glomeruli, sometimes with dilated Bowman spaces (Haumaitre et al., 2006; Nakayama et al., 2023). Since dysplastic tubules do not exactly resemble normal structures in the kidney, their nephron segment or CD origin has been unclear. In our study, we used a hPSC differentiation protocol which generates organoids that are rich in nephron components, especially glomeruli and PTs, but lacks differentiated CDs (Bantounas et al., 2018, 2021; Howden et al., 2021; Takasato et al., 2015; Wu et al., 2018). Indeed, this was reflected in RNA-seq of control organoids. Histological examination of non-mutant organoids showed that they contained avascular glomeruli together with LTL-binding tubules, consistent with PT identity (Kishi et al., 2019), or reacted with CDH1 antibody, consistent with DT identity (Nouwen et al., 1993). We showed that heterozygous mutant *HNF1B* hESCs or hiPSCs can form kidney-like organoid structures. This is consistent with the fact that individuals with *HNF1B* mutations do have kidneys, in contrast to certain other human genetic diseases (e.g., *FRAS1* or *FREM2* mutations) where organogenesis fails to initiate (Clissold et al., 2015).

### Roles for *HNF1B* in human kidney tubule morphogenesis and differentiation

The main abnormal morphological feature of heterozygous *HNF1B* mutant organoids was large-diameter multi-layered tubules rather than normal single-layered epithelial walls. This was found in both CRISPR-mutant hESC organoids and those derived from iPSCs from individuals with HNF1B-associated DKMs. Mutant organoids displayed low expression of transcripts characteristic of PTs and DTs. These included genes (e.g., *CLCN5*, *SLC34A1*, *SLC4A4*, *CLCNKA*, and *SLC12A1*) implicated in Mendelian diseases where tubules fail to function, resulting in urinary wasting of electrolytes and low-molecular-weight proteins, together with acid-base aberrations. These deficiencies help to explain the electrolyte wasting reported in *HNF1B*-associated DKMs (Adalat et al., 2019, 2009). It is unlikely that these changes result from simply absence of PTs or DTs because several other PT/DT-characteristic genes were similarly expressed in mutant and non-mutant organoids. Tubule maturation might simply be delayed in the mutant, but histological and scRNA-seq data suggest that heterozygous *HNF1B* mutations lead to the generation of aberrant nephron epithelia with features absent in healthy kidney, indicating an abnormal cell state. These features include enlarged and hyperproliferative tubules with hybrid identities including binding to LTL and expressing CDH1, and disordered polarity evidenced by replacement of apically localized Cubilin by a diffuse cytoplasmic pattern. This is further supported by our scRNA-seq results, which demonstrate that in mutant organoids there is an almost complete lack of cells with typical PT transcriptomes and an overrepresentation of proliferative cell types in both nephron tubules and glomeruli. Our molecular analyses also suggest that *HNF1B* mutant organoids display a modest upregulation of UB/CD lineage genes (e.g., *RET* and *SCNN1G)* consistent with a metaplastic shift of tubule cell identity. It has been reported that nephron-rich hPSC-kidney organoids display a degree of plasticity such that by manipulating them biochemically they can be redirected to a UB/CD identity (Howden et al., 2021). Gene editing of hiPSCs leading to biallelic *HNF1B* deletions was reported to result in organoids that fail to form PTs (Przepiorski et al., 2018). This gross deficit resembles the severely impaired development of nephron tubules in mice carrying biallelic *Hnf1b* deletions within nephron precursor cells (Heliot et al., 2013; Massa et al., 2013). In contrast, our current human heterozygous *HNF1B* mutant model generates a more nuanced phenotype comprising aberrant structural, molecular, and functional differentiation of nephron tubule segments, rather than their absence.

Compared with the morphological and molecular derangements of tubules in our heterozygous *HNF1B* mutant organoids, mutant glomeruli had a milder phenotype with more prominent tufts. Bulk RNA-seq showed generally preserved expression of podocyte genes, while scRNA-seq showed a shift toward a proliferative mutant podocyte population. In the mutant organoids, *HNF1B* transcripts were detected in glomerular tufts but absent in non-mutant glomeruli. The latter is consistent with native human fetal kidneys where *HNF1B* is expressed by tubules but not by glomerular tuft cells.

### Heterozygous *HNF1B* mutant organoids do not form cysts

*HNF1B*-associated DKMs can contain cysts, up to a few centimeters across (Haumaitre et al., 2006; Nakayama et al., 2023). On the other hand, a landmark clinical study reported a more complex situation (Decramer et al., 2007). Here, 18 fetuses, later found to have heterozygous *HNF1B* mutations, presented with ultrasonographically echo-bright kidneys, indicative of widespread abnormality of their internal structure. Despite this, only 11 of the 18 had overt kidney cysts. Notably, however, “… after birth, cysts appeared during the first year (17 of 18), and in patients with antenatal cysts, the number increased ….” This suggests that cystogenesis in *HNF1B* mutant kidneys is a relatively late feature that long postdates a disruption of normal nephron maturation. Indeed, in our study, although mutant organoid tubules contained statistically significantly larger lumina than those of wild-type tubules, overt cysts were not present despite marked downregulation of genes (e.g., *CYS1* and *PKHD1*) that maintain a healthy non-cystic phenotype in kidney epithelia (Nakanishi et al., 2000; Yang et al., 2021). In certain genetic human cystic kidney diseases, such as autosomal dominant PKD (ADPKD) associated with *PKD1* or *PKD2* mutations, *in vivo* kidney cystogenesis is largely driven by cAMP signaling (Richards et al., 2021). Compared with control counterparts, *HNF1B* mutant organoids resisted chemical induction by cAMP which generated dilated nephrons in control organoids consistent with a lack of mature function of mutant tubules. We conclude that the genesis of nephron cysts in *HNF1B*-associated DKMs may be a secondary, and late, feature probably not driven by cAMP. Perhaps the presence of glomerular filtration in conjunction with primary aberrations in tubule biology is needed to generate overt cysts in *HNF1B* disease. Implantation of heterozygous *HNF1B* mutant organoids into mice, as described for non-mutant hPSC-kidney progenitors (Bantounas et al., 2018, 2020), will allow extended development with vascularization of glomeruli such that dilatation may then occur in glomeruli and tubules. It is also possible that, had CDs been present, cysts may have occurred. Another group generated heterozygous *HNF1B* mutant hPSCs and differentiated them into UB/CD-like cells. These cells formed UB/CD organoids, and in mutants “the number of budding regions tends to be reduced” but notably cysts were not observed (Mae et al., 2020).

### Molecular mechanisms of *HNF1B*-associated DKMs

RNA-seq analyses of mutant organoids identified deregulation of many genes containing the canonical HNF binding sequence (Adalat et al., 2009). These included genes expressed by kidney tubule epithelia, e.g., *CLCN5*, *CYS1*, *HNF1A*, *HNF4A*, *KCNJ16*, *MUC1*, *PKHD1*, and *SLC34A1*, likely a direct effect of decreased functional HNF1B protein. Several key transcripts involved in kidney development, e.g., WNT pathway, were also deregulated yet do not contain the canonical HNF1B binding site. These changes may be secondary to direct HNF1B-induced gene regulation, but their altered expression may contribute to the DKM-like phenotype. A prominent example of such genes was the glutamate receptor subunit *GRIK3* that was markedly upregulated in heterozygous *HNF1B* mutant organoid differentiation. Moreover, scRNA-seq analyses showed that this upregulation occurred in cells high in *HNF1B*. In support of a role for glutamate receptors in disease, KEGG pathway analysis predicted deregulation of glutamatergic signaling, while, histologically, *GRIK3* transcripts and protein are noticeably higher in dysmorphic tubules of *HNF1B* mutant organoids and in mutant over control fetal kidney sections. Moreover, several glutamate receptor genes, or components of glutamatergic intracellular signaling machinery, were deregulated in mutant organoids. These observations are highly novel given that glutamate receptors have hitherto not been studied in either normal or abnormal human kidney development.

Glutamatergic signaling and glutamate metabolism have been intensively studied in neuronal tissues where glutamate acts as an excitatory neurotransmitter (Hansen et al., 2021). Roles for glutamatergic signaling, and GRIK3 itself, are emerging outside the nervous system. For instance, GRIK3 expression is prominent in breast cancer tissues, and it increases proliferation and migration of breast cancer cells *in vitro*, driving epithelial-to-mesenchymal transition (Xiao et al., 2019). GRIK3 is implicated in proliferation and migration of intestinal and lung cancer cells (Du et al., 2020). Of note, components of the glutamate signaling system have been identified in mature kidneys *in vivo* and in kidney epithelial cell lines (Hediger, 1999; Valdivielso et al., 2020), e.g., *GRIN2A* and *GRIN2B* in murine kidney tubules (Iwata et al., 2022) with *SLC7A11* and *SLC1A* in PT cells (Shayakul et al., 1997; Wang et al., 2021; Welbourne and Matthews, 1999). Increased monosodium glutamate dietary intake in rats leads to increased glomerular filtration rate, which the NMDA receptor antagonist MK-801 reduced (Mahieu et al., 2016). Glutamate transporters move extracellular glutamate into PTs, facilitating glutamine/glutamate metabolism, urinary acidification, and movement of bicarbonate back into the body (Welbourne and Matthews, 1999). Knockdown of *GRIN1* (NMDAR1) in a PT cell line led to an epithelial-to-mesenchymal transition, while addition of NMDA blunted *in vitro* expression of transforming growth factor β1 (TGF-β1)-induced mesenchymal markers (Bozic et al., 2011). Moreover, NMDA administration ameliorated kidney fibrosis triggered by ureteric obstruction (Bozic et al., 2011). In mice receiving chemical NMDAR blockade, urinary protein levels rise while, *in vitro*, antagonizing NMDAR causes cytoskeletal remodeling in podocytes (Giardino et al., 2009). These observations indicate that glutamate and glutamatergic signaling impact on the health of kidney epithelial cells. They are also consistent with the hypothesis that the deregulation of glutamatergic signaling genes such as *GRIK3* plays roles in the pathobiology of kidney dysplasia associated with *HNF1B* mutation.

An unanswered question is whether developing kidneys are exposed to glutamine/glutamate *in vivo*. Amino acids are present in the milieu of early developing embryos (Van Winkle, 2021), and human cord blood at term contains glutamate, which increases with fetal distress (Perez-Mato et al., 2016). In the central nervous system, astrocytes release glutamine that is taken up by neurons to generate glutamate for use in neurotransmission (Andersen and Schousboe, 2022). Whether a similar glutamate-producing mechanism operates in the kidney is unknown. Alternatively, kidney glutamate receptors may function to sense other amino acids in the proto-urine: for example, d-serine activates NMDARs in the kidney resulting in Ca^2+^-mediated increase in reactive oxygen species, leading to renal insufficiency in mice. Many of the resulting symptoms are reversed by NMDAR inhibitors (Tseng et al., 2021). Finally, drugs that modulate glutamate signaling are being explored as treatments in non-renal (e.g., brain) diseases (Stone, 2011), and this suggests that similar drugs may ameliorate features of kidney organogenesis associated with *HNF1B* mutations.

## Experimental procedures

### Resource availability

#### Lead contact

Susan J. Kimber (sue.kimber@manchester.ac.uk)

#### Materials availability

Materials generated in this study are available from the Kimber lab upon request.

#### Data and code availability

Generated RNA-seq and scRNA-seq datasets can be found at ArrayExpress under the following accession numbers: ArrayExpress: E-MTAB-12824 (RNA-seq of hESC/organoids), ArrayExpress: E-MTAB-12822 (RNA-seq of native kidney), and ArrayExpress: E-MTAB-13500 (scRNA-seq).

### hPSC cell culture

See supplemental experimental procedures.

### CRIPSR-Cas9^n^ editing of hESCs

Two artificially created inserts for gRNAs, targeting *HNF1B* at positions 231 of the coding strand and 171 of the complementary strand, and containing the appropriate overhangs, were cloned into BbsI-digested pX461 plasmid (Addgene, #48140), which also expresses the nickase (D10A) version of Cas9 (Cas9^n^) and a GFP tag. The resultant plasmids were then both transfected into MAN13 hESCs using a Lonza 4D-Nucleofector and the Amaxa P3 Primary Cell 4D-Nucleofector X Kit L according to the manufacturer’s instructions. Transfected cells were identified and sorted by GFP fluorescence at 5-10 × 10^3^ cells per well in 6-well plates for up to 15 days, and then each was transferred into separate wells and expanded. Edited clones were identified by sequencing. (See supplemental experimental procedures).

### iPSC derivation

PBMCs were isolated by gradient centrifugation on Ficoll and expanded before being transduced with the CytoTune-iPS 2.0 Sendai Reprogramming Kit (A16517, Thermo Fisher Scientific), according to the manufacturer’s instructions. See also supplemental experimental procedures.

### 2D and organoid differentiation

2D and organoid differentiation was performed by an adaptation of previously described protocols as described in supplemental experimental procedures.

### Next-generation RNA-seq and scRNA-seq

Both RNA-seq and scRNA-seq were performed by the University of Manchester Genomics Facility. Detailed methods are in supplementary experimental procedures.

### Immunological methods

Western blotting, immunocytochemistry of 2D cultures, and immunohistochemistry of paraffin-embedded, sectioned organoid, and human tissues were performed using standard protocols (see supplemental experimental procedures).

### *In situ* RNA hybridization (BaseScope)

Organoids were fixed in 4% paraformaldehyde, paraffin embedded, and sectioned at 5 μm. BaseScope ISH (ACDBio, Newark, CA, USA) was conducted following manufacturer’s instructions, using the BaseScope detection reagent Kit v2-RED. See also supplemental experimental procedures.

### cAMP-induced tubule dilatation and cell proliferation assay

See supplemental experimental procedures.

### Bioinformatics analyses

GO and KEGG pathway analyses, as well as *in silico* identification of promoters directly bound by HNF1B, are described in supplemental experimental procedures.

### Quantification and statistical analysis

Detailed statistical methods for each experiment are given in supplementary experimental procedures.

## Supplemental information

Document S1. Figures S1–S7, Tables S1–S6, and supplemental experimental procedures

Document S2. Article plus supplemental information
