# Supplementary material for: Human pluripotent stem cell-derived kidney organoids reveal tubular epithelial pathobiology of heterozygous HNF1B-associated dysplastic kidney malformations
Source: Stem Cell Reports. 2024 May 23;19(6):859–76. doi: 10.1016/j.stemcr.2024.04.011 (PMC11297557; doi:10.1016/j.stemcr.2024.04.011)
Supplement: Document S2. Article plus supplemental information [file mmc2.pdf]

Stem Cell Reports

Article

OPEN ACCESS

Human pluripotent stem cell-derived kidney organoids reveal tubular epithelial pathobiology of heterozygous *HNF1B*-associated dysplastic kidney malformations

Ioannis Bantounas,[1,9] Kirsty M. Rooney,[1,9] Filipa M. Lopes,[1] Faris Tengku,[1,7] Steven Woods,[1] Leo A.H. Zeef,[2] I-Hsuan Lin,[2] Shweta Y. Kuba,[1,8] Nicola Bates,[1] Sandra Hummelgaard,[1,3] Katherine A. Hillman,[4] Silvia Cereghini,[5] Adrian S. Woolf,[1,6,10,*] and Susan J. Kimber[1,10,11,*]

[1]Division of Cell Matrix Biology and Regenerative Medicine, Faculty of Biology, Medicine and Health, University of Manchester, and the Manchester Academic Health Science Centre, Manchester, UK

[2]Bioinformatics Core Facility, University of Manchester, Manchester, UK

[3]Department of Biomedicine, Aarhus University, Denmark

[4]Manchester Institute of Nephrology and Transplantation, Manchester University NHS Foundation Trust, Manchester, UK

[5]Sorbonne Université, CNRS, Institut de Biologie Paris Seine, Laboratorial de Biologie du Développement, IBPS, UMR7622, F-75005 Paris, France

[6]Royal Manchester Children's Hospital, Manchester University NHS Foundation Trust, Manchester, UK

[7]Present address: Kulliyyah of Nursing, International Islamic University Malaysia, Kuala Lumpur, Malaysia

[8]Present address: School of Health and Life Sciences, National Horizons Center, Teesside University, Darlington, DL1 1HG, United Kingdom

[9]These authors contributed equally

[10]These authors contributed equally

[11]Lead contact

*Correspondence: adrian.woolf@manchester.ac.uk (A.S.W.), sue.kimber@manchester.ac.uk (S.J.K.)

https://doi.org/10.1016/j.stemcr.2024.04.011

SUMMARY

Hepatocyte nuclear factor 1B (*HNF1B*) encodes a transcription factor expressed in developing human kidney epithelia. Heterozygous *HNF1B* mutations are the commonest monogenic cause of dysplastic kidney malformations (DKMs). To understand their pathobiology, we generated heterozygous *HNF1B* mutant kidney organoids from CRISPR-Cas9 gene-edited human embryonic stem cells (ESCs) and induced pluripotent stem cells (iPSCs) reprogrammed from a family with *HNF1B*-associated DKMs. Mutant organoids contained enlarged malformed tubules displaying deregulated cell turnover. Numerous genes implicated in Mendelian kidney tubulopathies were downregulated, and mutant tubules resisted the cyclic AMP (cAMP)-mediated dilatation seen in controls. Bulk and single-cell RNA sequencing (scRNA-seq) analyses indicated abnormal *Wingless/Integrated* (WNT), calcium, and glutamatergic pathways, the latter hitherto unstudied in developing kidneys. Glutamate ionotropic receptor kainate type subunit 3 *(GRIK3)* was upregulated in malformed mutant nephron tubules and prominent in *HNF1B* mutant fetal human dysplastic kidney epithelia. These results reveal morphological, molecular, and physiological roles for HNF1B in human kidney tubule differentiation and morphogenesis illuminating the developmental origin of mutant-*HNF1B*-causing kidney disease.

INTRODUCTION

Mammalian kidney development is a complex process (Jafree et al., 2019; Wilson and Little, 2021; Woolf, 2019). The human metanephric kidney initiates at 5 weeks gestation (Woolf, 2019) when the metanephric mesenchyme (MM) is penetrated by the ureteric bud (UB). MM/UB crosstalk results in MM undergoing mesenchymal-to-epithelial transition to generate primitive nephrons, each differentiating into a glomerulus, a proximal tubule (PT), and the distal nephron tubule (DT), including the loop of Henle and the distal convoluted tubule (McMahon, 2016). Waves of nephrogenesis forming glomeruli and nephron tubules occur between antenatal weeks 7 and 34, and the UB arborizes into collecting ducts (CDs), each fusing with a DT. Stromal fibroblast-like cells (Wilson and Little, 2021) and blood and lymphatic endothelia (Jafree et al., 2019) are found between developing tubules. Capillaries invade glomerular podocyte tufts delivering blood for filtration, and tubules modify the ultrafiltrate to make definitive urine. Dysplastic kidney malformations (DKMs) are major causes of kidney failure in children and young adults (Kohl et al., 2022). Ultrasonography detects echo-bright organs that, lacking cortical-medullary distinction (Kohl et al., 2022), manifest aberrant nephron morphogenesis and deregulated cell turnover (Winyard et al., 1996a, 1996b).

The commonest genetic DKMs are due to heterozygous mutations of *hepatocyte nuclear factor 1B* (*HNF1B*) (Adalat et al., 2009, 2019). *HNF1B* encodes a homeodomain transcription factor that dimerizes with itself or *HNF1A* (Clissold et al., 2015). *HNF1B*-associated kidney disease can vary in severity, without clear genotype-phenotype correlations (Lim et al., 2020). Some fetuses undergo termination (Haumaitre et al., 2006). Others survive but postnatally exhibit urinary electrolyte wasting, suggesting that HNF1B is required for tubular functional differentiation (Adalat et al., 2019, 2009). *HNF1B* is expressed in developing and mature human nephron epithelia and CDs

This is an open access article under the CC BY license (http://creativecommons.org/licenses/by/4.0/).

(Haumaitre et al., 2006; Kolatsi-Joannou et al., 2001) but not in the MM, stroma, or mature podocytes. *HNF1B*-associated DKMs contain abnormal multi-layered tubules and dysmorphic glomeruli (Haumaitre et al., 2006; Nakayama et al., 2023).

HNF1B has been manipulated in kidney somatic cell lines (Chan et al., 2020; Piedrafita et al., 2021), *Xenopus* (Grand et al., 2023), and mice. Cre-mediated deletion of both alleles in mouse metanephroi deregulates expression of genes including those implicated in WNT signaling and polycystic kidney disease (PKD) (Desgrange et al., 2017; Fiorentino et al., 2020; Gresh et al., 2004; Lokmane et al., 2010). Postnatal biallelic deletion perturbs mouse kidney mitochondrial respiration (Casemayou et al., 2017), enhances fibrosis (Chan et al., 2018), and impairs regeneration (Verdeguer et al., 2010). Germline homozygous mutant mice are early embryonic lethal, and humans with biallelic germline mutations have not been described. Thus, the relevance of biallelic models to understand human heterozygous *HNF1B*-associated DKMs is uncertain. Recently, however, aberrant kidney tubules were described in mice carrying a heterozygous intron 2 splice donor site germline *Hnf1b* mutation (Niborski et al., 2021). Nevertheless, kidney gene expression patterns are not always identical in humans and mice (Lindstrom et al., 2018), so new human experimental models are needed.

Kidney organoids from human pluripotent stem cells (hPSCs) are being used to understand normal and abnormal kidney development (Bantounas et al., 2018; Rooney et al., 2021; Taguchi et al., 2014; Takasato et al., 2015; Woolf, 2019). We hypothesized that such hPSC-derived organoids would model human *HNF1B*-associated DKMs. We therefore undertook morphological, functional, and molecular analyses of heterozygous *HNF1B* mutant organoids derived from CRISPR-Cas9 gene-edited wild-type human embryonic stem cells (hESCs) or from human induced pluripotent stem cells (iPSCs) (hiPSCs) reprogrammed from peripheral blood mononuclear cells (PBMNs) of siblings with *HNF1B*-associated DKMs. Mutant organoids displayed malformed nephrons and deregulated cell turnover. Genes implicated in Mendelian tubulopathies were downregulated in mutant organoids which resisted cyclic AMP (cAMP)-mediated tubule dilatation, seen in unaffected controls. Bioinformatic analyses predicted abnormal pathways, including WNT, and glutamatergic signaling, the latter hitherto unstudied in kidney development. Glutamate ionotropic receptor (iGluR) kainate type subunit 3 (GRIK3) was markedly upregulated in mutant organoids and detected in human fetal *HNF1B*-associated DKM tubules. Our results illuminate important roles for HNF1B in human kidney development and identify potentially druggable targets.

RESULTS

Generating heterozygous *HNF1B* mutant hESCs

CRISPR-Cas9 gene editing was used to mutate *HNF1B* in MAN13 hESCs (hPSCreg-ID: UMANe002-A) and create the IBM13-19 heterozygous line (hPSCreg-ID: UMANe002-A-4; hereafter called "mutant") (Figure S1A), which harbors a frameshift in exon 1 (Figure S1B), resulting in a premature stop codon 51 nucleotides downstream and a protein lacking the DNA binding and transactivation domains. A non-mutated clone (IBM13-08; hPSCreg-ID: UMANe002-A-5) was used as isogenic control line (Figure S1B). Using a 2D kidney differentiation protocol (Bantounas et al., 2018, 2021), we observed an attenuated immunohistochemical HNF1B signal in the mutant with an antibody against a 111 amino acid epitope that would be disrupted by the mutation (Figure S1C). We next generated mutant and unaffected kidney organoids (Bantounas et al., 2018, 2021; Takasato et al., 2015) (Figure S1D) of similar size (Figures S1E and S1F), but phase contrast suggested larger internal structures in mutant organoids (Figure S1G). Quantitative reverse-transcription PCR (RT-qPCR) using primers that would detect both non-mutant and mutant *HNF1B* mRNAs showed similar non-mutant and mutant levels (Figure 1A), while HNF1B protein was, as expected, significantly decreased in the mutant (Figures 1B and 1C). RNA *in situ* hybridization (ISH) with a probe detecting non-mutant and mutant HNF1B transcripts showed tubular expression, including in aberrant mutant tubules (Figure 1D). HNF1B immunohistochemistry followed this pattern, but HNF1B was attenuated in the mutant (Figure 1E).

Histology and functionality of hESC-derived organoids

Analysis of the organoids revealed bulkier tubules (Figures 2A and 2B) in mutant organoids. In control organoids *Lotus tetragonolobus* lectin (LTL), a PT marker (Kishi et al., 2019), bound a subset of slender tubules in a uniform manner (Figure 2C), whereas mutant dysmorphic tubules exhibited patchy staining (Figure 2D). In native human kidneys, DTs and CDs are rich in CDH1 (Nouwen et al., 1993). In control organoids, a subset of tubules was CDH1+ (Figure 2E), and mutant dysmorphic tubules showed patchy immunostaining (Figure 2F). For both LTL+ and CDH1+ tubules, the cross-sectional areas of mutant tubules were significantly larger than those of wild-type tubules (Figures 2G–2I). *In vivo*, Megalin and Cubilin form a receptor complex on the apical (luminal) plasma membrane of PTs (Nielsen et al., 2016). Slender control organoid tubules displayed this apical pattern (Figures 2J and 2L), but bulky mutant tubules showed

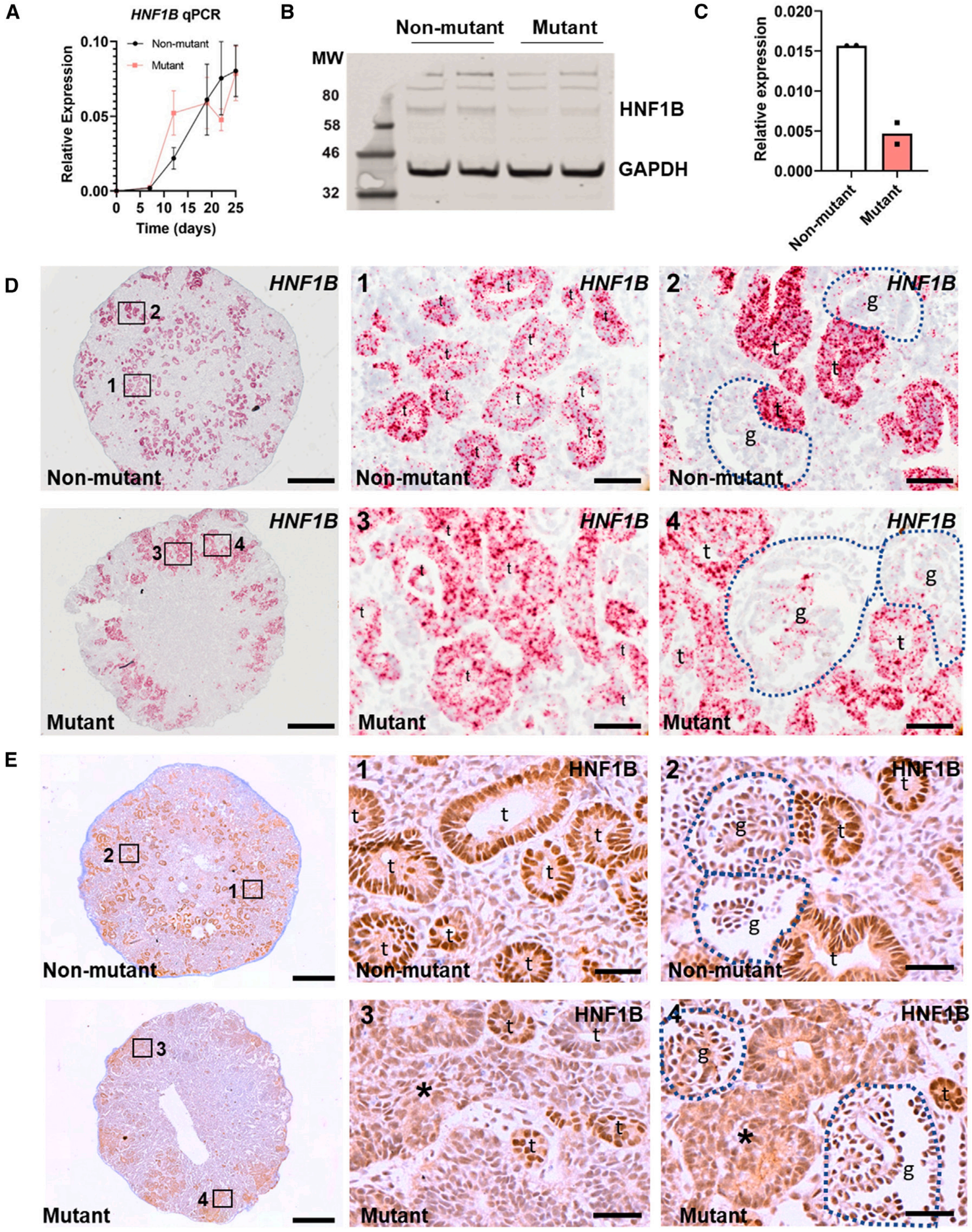

Figure 1. HNF1B in hESC-derived organoids

(A) RT-qPCR using *HNF1B* exon 2 primers showed similar upregulation in *mutant* and isogenic *non-mutant* lines over 25 days; bars denote standard error of the mean (SEM). (B) Western blot of day 25 organoids (two organoid pools/genotype) detected HNF1B doublets at 63 kDa.

(C) Decreased HNF1B/GAPDH values in mutant organoids (mean and individual values shown).

(legend continued on next page)

predominantly cellular Cubilin immunostaining (Figure 2K), while Megalin was absent (Figure 2M). Synaptopodin+ (a podocyte, actin-associated protein) glomeruli appeared larger with less compact podocyte tufts in the mutant (Figures S1H, and S1I). Control and mutant organoids contained PECAM1+ endothelia between tubules, but neither featured capillaries within glomerular tufts (Figures S1J and S1K). We measured bromodeoxyuridine (BrdU) incorporation (proliferation) and activated Caspase-3 immunostaining (apoptosis) (Figures S1L–S1Q). BrdU+ tubule nuclei and apoptotic figures were significantly increased across the whole organoid in mutants. Forskolin (FSK) is an adenylate cyclase activator that increases intracellular 3′,5′-cAMP. When wild-type metanephroi are exposed to FSK in organ culture, nephron lumina dilate, reflecting fluid transport and tubule functionality (Anders et al., 2013). Adding FSK to organoids (Figure 3A) generated numerous translucent areas in controls, whereas few were evident in mutants (Figure 3B). Both the numbers and the area occupied by these dilatations were significantly less in mutants (Figures 3C–3H). Probing with LTL or for CDH1 or Synaptopodin (Figures 3I–3N) suggested that dilatations in non-mutant organoids affected PTs, DTs, and glomeruli. The smaller dilated structures in mutants were harder to categorize, although some affected glomerular tufts (Figure 3N).

Organoid differentiation from *HNF1B* mutant patient-derived hiPSCs

To determine whether the phenotype of CRISPR-Cas9-mutated hESC-derived organoids was representative of *HNF1B*-associated kidney disease, we evaluated organoids generated from hiPSCs derived from a family carrying a heterozygous deletion of exon 9 ($HNF1B^{+/\Delta Exon9}$) (Figures S2A–S2C). HiPSCs from two brothers with DKMs (TF171A; hPSCreg-ID: UMANCi003-A and TF172D; hPSCreg-ID: UMANCi002-A) and their unaffected $HNF1B^{+/+}$ mother (TF173B; hPSCreg-ID: UMANCi001-A) were generated, and the mutation was confirmed by genomic qPCR (Figure S2D). *HNF1B* transcripts, assessed using exon 2 primers, were similar in control and mutant organoids (Figure S2E), but, using mutation-specific (exon 9) primers, transcripts were lower in mutant organoids (Figure S2E). Similarly to the mutant hESC-derived organoids, $HNF1B^{+/\Delta Exon9}$ organoids exhibited dysmorphic tubules with multi-layered epithelia and bulky glomeruli (Figure S2F) and were unable to form cysts after 8-Br-cAMP (Figures S2G–S2I).

Overview of hESC-derived organoid transcriptomes

For model validity, we compared bulk RNA sequencing (RNA-seq) profiles of differentiating control hESCs with human kidneys at 10–12 weeks gestation when they contain a nephrogenic cortex of MM and branching UB tips, with deeper maturing nephrons, CDs, and stromal cells. Principal-component analysis (PCA) showed that organoid profiles approached those of native fetal kidneys as differentiation progressed (Figure S3A), with key glomerular and PT marker genes, but fewer DT and CD transcripts (Figures S3B–S3G).

Moreover, the transcriptional profiles of control and mutant organoids had similar temporal progression (Figure S3J) during differentiation. When the levels of *HNF1B* rose markedly at day 12 (Figure S3K), the number of significantly differentially expressed genes (DEGs) also increased, up to 631 on day 25 (Figures S3L and S3M). Among these were genes containing the canonical *HNF1B* binding motif upstream of their transcription start site (Figure S3N-RI). Gene ontology (GO) enrichment analysis and Kyoto Encyclopedia of Genes and Genomes (KEGG) pathway analysis, comparing mutant and isogenic control organoids (Figure S4 and Table S4), showed that calcium ion binding, cadherin binding, collagen containing extracellular matrix, and integral component of plasma membrane were dysregulated, along with WNT signaling, a pathway essential for kidney development (with *WNT5A*, *WNT10B*, *FZD4*, and *DKK1* among DEGs).

Characteristic kidney transcripts in *HNF1B* mutant hESC-derived organoids

We examined organoids for cell type-specific or cell type-enriched transcripts in native kidneys. Heatmaps in Figures 4A–4H show transcripts detectable in control and mutant organoids during differentiation (further validating the organoid model), while genes differentially expressed in the mutant are shown in volcano plots (Figures 4I–4K). Strikingly, numerous genes differentially expressed between mutant and non-mutant are implicated in Mendelian kidney diseases (especially tubulopathies), including various channels/transporters (Table S5). Of

(D) BaseScope ISH *HNF1B* (red dots) with nuclei counterstained blue with hematoxylin. Left-hand frames show low power overviews with enlarged areas in boxes 1–4. Non-mutant tubules (*t* in 1) expressed *HNF1B*, but signal was scarce in glomeruli (*g* in 2). In mutants, *HNF1B* was expressed in bulky, aberrant tubules (*t* in 3) and in tufts of aberrant-looking glomeruli (*g* in 4).
(E) HNF1B immunostaining (brown) in day-25 organoids, with hematoxylin counterstain. Left-hand frames; overviews with boxes 1–4 enlarged in the other frames. HNF1B was detected in nuclei of wild-type tubules (*t* in 1 and 2). In mutants, HNF1B was in small-caliber tubules (*t* in 3 and 4), but signals were attenuated and diffuse in bulky, aberrant tubules (*asterisks* in 3 and 4). Bars: (D) 200 μM (overview) and 20 μM (enlargements); and (E) 500 μM (overview) and 40 μM (enlargements).

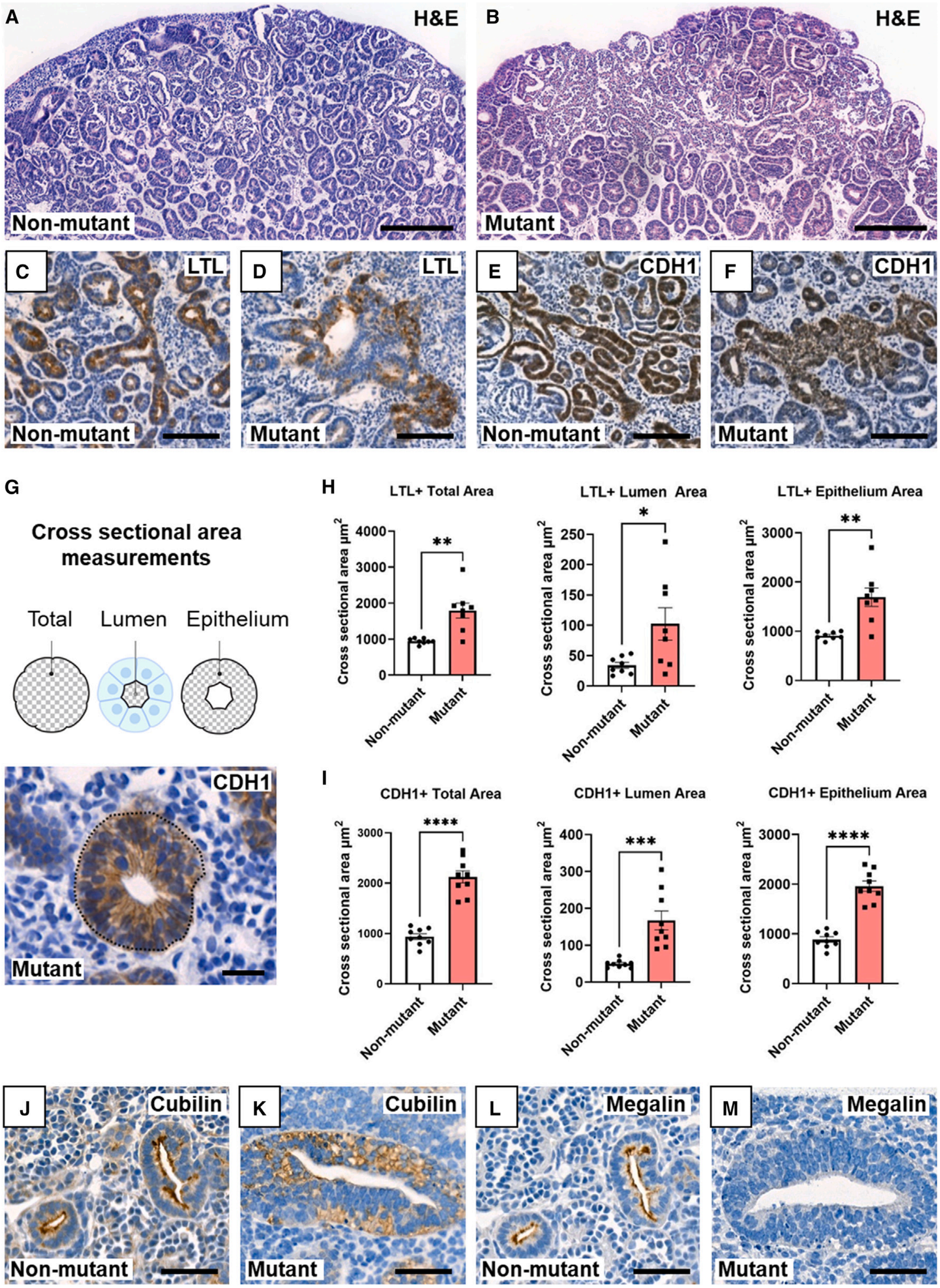

(legend on next page)

those, *HNF1A*, a transcription factor binding partner of HNF1B causing PT dysfunction when mutated in mice, was significantly reduced in the mutant, and ISH experiments (Figure S5) detected transcripts in control organoid tubules but a lower signal in mutants.

Additionally, in the glomerulus, basement membrane gene *LAMB1* was significantly lower in day-25 mutants, while podocyte gene *PODXL* was up at day 19 (Figures 4J and 4K). In PTs (additional to genes in Table S5), *VIL1*, encoding brush border Villin, was significantly lower in mutants (Figures 4J and 4K); DT and CD gene *SGK1* (serum/glucocorticoid-regulated kinase 1) and RET (in branching UB branch tips) were upregulated in mutants (Figures 4J and 4K). Genes associated broadly with tubule epithelia were downregulated in mutants (Figures 4I–4K) including *MET* (HGF receptor), autosomal recessive PKD-associated gene *CYS1* (Cystin), and *PKHD1* (Fibrocystin) (Table S5). Considering MM and primitive nephron-expressed transcripts (Figure 4F), *NCAM1* and *PAX8* were higher in mutants (Figures 4I–4K), while *GREM1*, a BMP4-antagonist, was lower (Figure 4K). Among transcripts implicated in differentiation of endothelia and vascular smooth muscle cells (SMCs), *ANGPT1* was significantly higher in mutant organoids, while *PROX1* was lower (Figures 4J and 4K).

scRNA-seq analysis reveals aberrant cell populations in nephrons of mutant organoids

To better understand the molecular and cellular basis of the developmental aberrations, we performed single-cell RNA-seq (scRNA-seq) comparing day-25 non-mutant and mutant organoids, analyzing 6,168 non-mutant and 5,783 mutant cells. Clustering analysis identified 23 distinct cell populations (Figures 5A and 5B). These were annotated by significant known key marker gene expression in each cluster (Table S6). Mutant organoids almost completely lacked molecularly typical PT and thick ascending limb loop of Henle populations (clusters 12 and 11 in Figures 5A–5D). This was consistent with the mutant organoid downregulation of transcripts normally expressed by these tubules, revealed in the bulk RNA-seq analysis. Concordant with histological glomerular aberrations, we identified differences in the podocyte populations between non-mutant and mutant organoids (clusters 1, 2, and 3, in Figures 5A–5D). Cluster 1 was extensively depleted of mutant cells while proliferating podocytes (cluster 3) were more abundant in mutant organoids (~72% mutant cells) (Figure 5D), and gene expression indicated they were in G2M or S phase.

We also observed differences in three clusters representing nephron progenitor cells between non-mutant and mutant organoids (clusters 4, 5, and 6, in Figures 5A and 5B). Cluster 6 comprised mostly (~82%) non-mutant cells, while cluster 5 contained non-mutant and mutant cells approximately equally (Figure 5D). Strikingly, cluster 4 contained 84% mutant cells and was highly proliferative with 97% of cells in G2M or S phase from their expression.

Deregulated glutamate receptor and pathway genes in mutant organoids

GRIK3, encoding iGluR kainate type subunit 3, was among the ten most deregulated transcripts in mutants in the bulk RNA-seq. This prompted us to examine iGluR expression (known for its role in neurotransmission and neuronal plasticity (Hansen et al., 2021) in human kidney. In addition to *GRIK3*, several iGluR subunits from all receptor subfamilies (N-methyl-D-Aspartate [NMDA], α-amino-3-hydroxy-5-methyl-4-isoxazole-propionic acid [AMPA], Kainate, Delta) were expressed in fetal human kidneys and organoids (Figure S6A).

GRIK3 levels in human fetal kidneys were similar to those in control organoids (Figure S6A), but *GRIK3* was significantly upregulated on days 12, 19, and 25 in mutant compared with control organoids (Figure S6B). On the final day of culture, *GRIK3* expression was 10 times higher (adjusted p value (p(adj)) = 1.95×10^{-31}) in *HNF1B* mutant than control organoids (Figure 6A). Western blotting

Figure 2. Aberrant tubules in *HNF1B* mutant organoids

(A) Non-mutant and (B) mutant organoids counterstained with hematoxylin and eosin. Internal structures appeared larger in mutants.

(C) Non-mutant and (D) mutant organoids stained with LTL (brown) with hematoxylin counterstain, showing slender LTL+ non-mutant tubules and bulky mutant tubules with patchy staining.

(E and F) (E) Non-mutant and (F) mutant organoids immunostained for CDH1 (brown) with hematoxylin counterstain, showing slender CDH1+ wild-type tubules and bulky mutant tubules with patchy staining.

(G) Above: cartoon showing the total, lumen, and epithelium areas measured from perpendicularly cross-sectioned tubules. Below: example tubule immunostained (brown) for CDH1 in mutant.

(H) Area of LTL+ profiles.

(I) Areas of CDH1+ profiles (in H and I: mean ± SEM; $n = 9$ organoids from three independent differentiation experiments; *$p < 0.05$, **$p < 0.005$, ***$p < 0.0005$, ****$p < 0.00005$, t test).

(J and K) Cubilin immunostaining (brown): apical pattern in non-mutant tubules but a diffuse pattern in mutants.

(L and M) Megalin immunostaining: apical pattern in non-mutant tubules (L) but not detected in mutant tubules (M). Bars: (A and B) 200 μM; (C–F) 100 μM; (G) 20 μM; and (J–M) 50 μM.

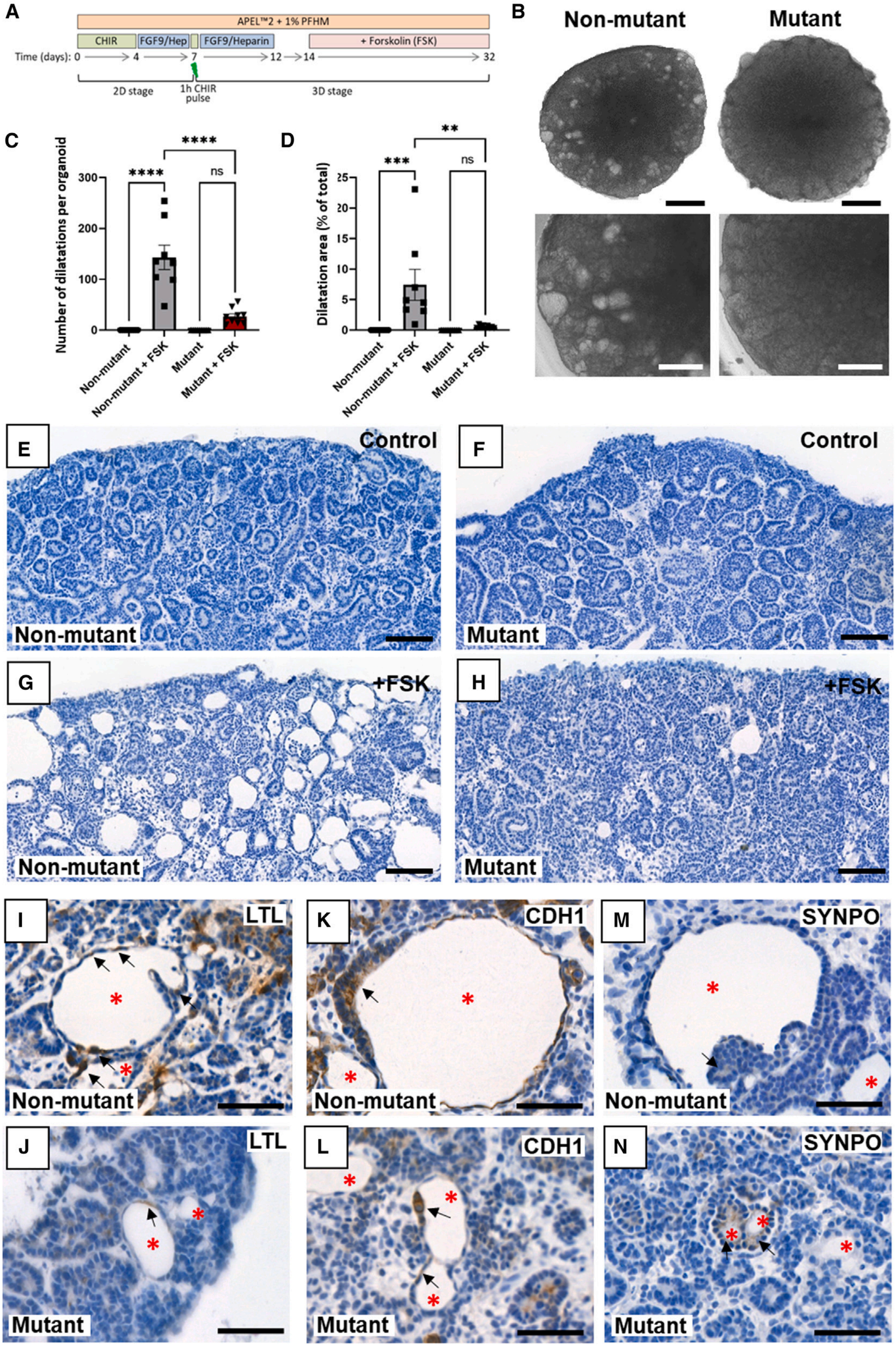

(legend on next page)

showed significantly increased GRIK3 protein in mutant organoids (Figures 6B and 6C). In the scRNA-seq, the mutant cells dominating cluster 4 were characterized by high *GRIK3* expression (Figure 5C). Finally, cluster 8 in the scRNA-seq comprised exclusively mutant cells and represented a unique tubular cell type with high *GRIK3* expression (Figure 5C). Indeed, ISH (Figure 6D) of control organoids detected *GRIK3* in tubules and more sparsely in interstitial cells, while *GRIK3* was prominently expressed in mutant dysplastic tubules. Immunohistochemistry for GRIK3 showed a predominantly tubular pattern in both control and mutant organoids (Figure 6E). On third-trimester fetal kidney sections (Figure S6G–S6P) GRIK3 immunostaining was detected in tubules. Some such tubules had apical periodic acid Schiff (PAS) staining, marking them as PTs. In a fetus carrying a heterozygous *HNF1B* mutation, GRIK3 was prominent in dysplastic tubules, some likely PTs (based on apical PAS). *GRIK3* was highly expressed in nephron progenitors in scRNA-seq and distal nephron populations in mutant organoids (clusters 4, 7, 8, 9, and 10), all also expressing *HNF1B*, supporting association between mutant *HNF1B* and *GRIK3* overexpression (Figures 7A and 7B), although we cannot conclude that *GRIK3* is under direct transcriptional control of *HNF1B* (Figures S3N–S3Q).

iGluR subunits of other subfamilies were also expressed in both fetal kidneys and organoids, with *GRIN2B*, *GRIN3A*, and *GRID1* overexpressed in mutant organoids (Figures S6A–S6E). KEGG enrichment analysis indicated that "glutamatergic synapse signaling" and "calcium signaling" pathways differed between mutant and control organoids (Figure S7, and Table S4), so we examined downstream members of the iGluR signaling pathway: In the mutant, we identified early (day-12) overexpression of glutamate transporters *SLC12A6* and *SLCA11*, followed by day-25 overexpression of Ca^{2+}-channel scaffold *HOMER2*, Synaptotagmin-1 (*SYT1*), and *CAMK2A* (Figure S6F). Conversely, the glutamate transporter SLC1A1, the iGluR scaffold *SHANK2*, and the downstream effector of high [Ca^{2+}], *PLCB1*, were downregulated in the mutant. Supporting the prevalence of transporter deregulation, GO Cellular Compartment enrichment analysis revealed significant gene enrichment in terms associated with the cell surface (p(adj) < 0.01, Figures S4G–S4I), notably well over 100 genes in the term "integral components of plasma membrane" at day 12, 19, and 25. These included *LRP2*, *PODXL*, *NPHS2*, *PKHD1*, and *MUC1* as well as 11 members of the extensive membrane transporter SLC genes, across a number of solute transport subfamilies, at day 19 and 25.

DISCUSSION

Our results reveal important roles for HNF1B in human kidney tubule morphogenesis and functional differentiation and suggest druggable targets to ameliorate disease.

Heterozygous *HNF1B* mutant organoids mimic features of human *HNF1B*-associated DKMs

Human kidneys with DKMs initiate organogenesis, yet their internal organization is deranged (Kohl et al., 2022; Winyard et al., 1996a, 1996b). With regard to *HNF1B*-associated DKMs, histological features include large multi-layered tubules and dysmorphic glomeruli, sometimes with dilated Bowman spaces (Haumaitre et al., 2006; Nakayama et al., 2023). Since dysplastic tubules do not exactly resemble normal structures in the kidney, their nephron segment or CD origin has been unclear. In our study, we used a hPSC differentiation protocol which generates organoids that are rich in nephron components, especially glomeruli and PTs, but lacks differentiated CDs (Bantounas et al., 2018, 2021; Howden et al., 2021; Takasato et al., 2015; Wu et al., 2018). Indeed, this was reflected in RNA-seq of control organoids. Histological examination of non-mutant organoids showed that they contained avascular glomeruli together with LTL-binding tubules, consistent with PT identity (Kishi et al., 2019), or reacted with CDH1 antibody, consistent with DT identity (Nouwen et al., 1993). We showed that heterozygous mutant *HNF1B* hESCs or hiPSCs can form kidney-like organoid structures. This is consistent with the fact that individuals with *HNF1B* mutations do have kidneys, in contrast to certain other human genetic diseases (e.g., *FRAS1* or

Figure 3. Deficient cAMP-induced lumen dilatation in *HNF1B* mutant organoids
(A) Organoids were exposed to forskolin (FSK) between 14 and 32 days.
(B) Phase contrast images at day 32 showed that FSK had induced numerous dilated structures in non-mutant organoids but few in mutants.
(C) Numbers of dilatations per organoid on histology.
(D) Quantification of total dilated percentage area per organoid (C and D: mean ± SEM; $n = 9$ organoids across 3 independent experiments; $****p < 0.00005$, $***p < 0.0005$, $**p < 0.005$, one-way ANOVA with multiple comparisons).
(E–H) Hematoxylin stained sections of non-mutant and mutant organoids, without (control) or with added FSK (+FSK).
(I–N) Organoid sections reacted with LTL or immunoprobed for CDH1 or SYNPO and counterstained with haematoxylin. Red asterisks indicate dilated structures; black arrows indicate associated cells. Bars: (B) 1 mm (upper panels) and 500 μM (lower panels); (E–H) 200 μM; and (I–N) 50 μM.

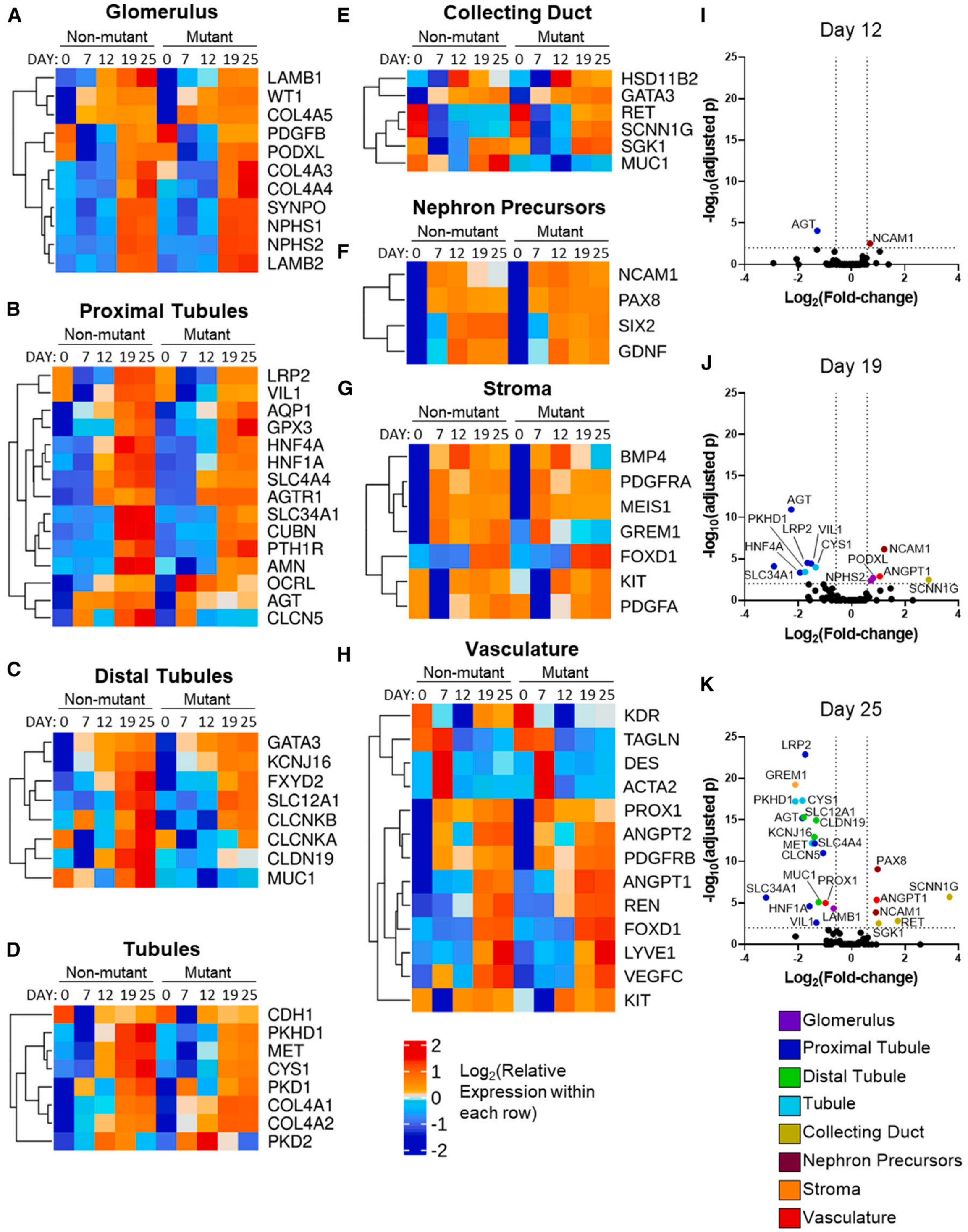

Figure 4. Profiles of established kidney transcripts in hESC-derived organoids

(A–H) Heatmaps through the differentiation protocol, with days 12, 19, and 25 being the organoid phase. Each element in the heatmaps represents the mean of three independent differentiation experiments. Genes were included if their average read count >50 on at least one day of organoid differentiation.

(I–K) Volcano plots showing significantly deregulated transcripts at days 12 (I), 19 (J), and 25 (K) with cut-offs of a $\log_2$(fold change) of 0.5 and $\log_{10}$(p-adjusted) significance value of 2 with expected lineage color coded; key below.

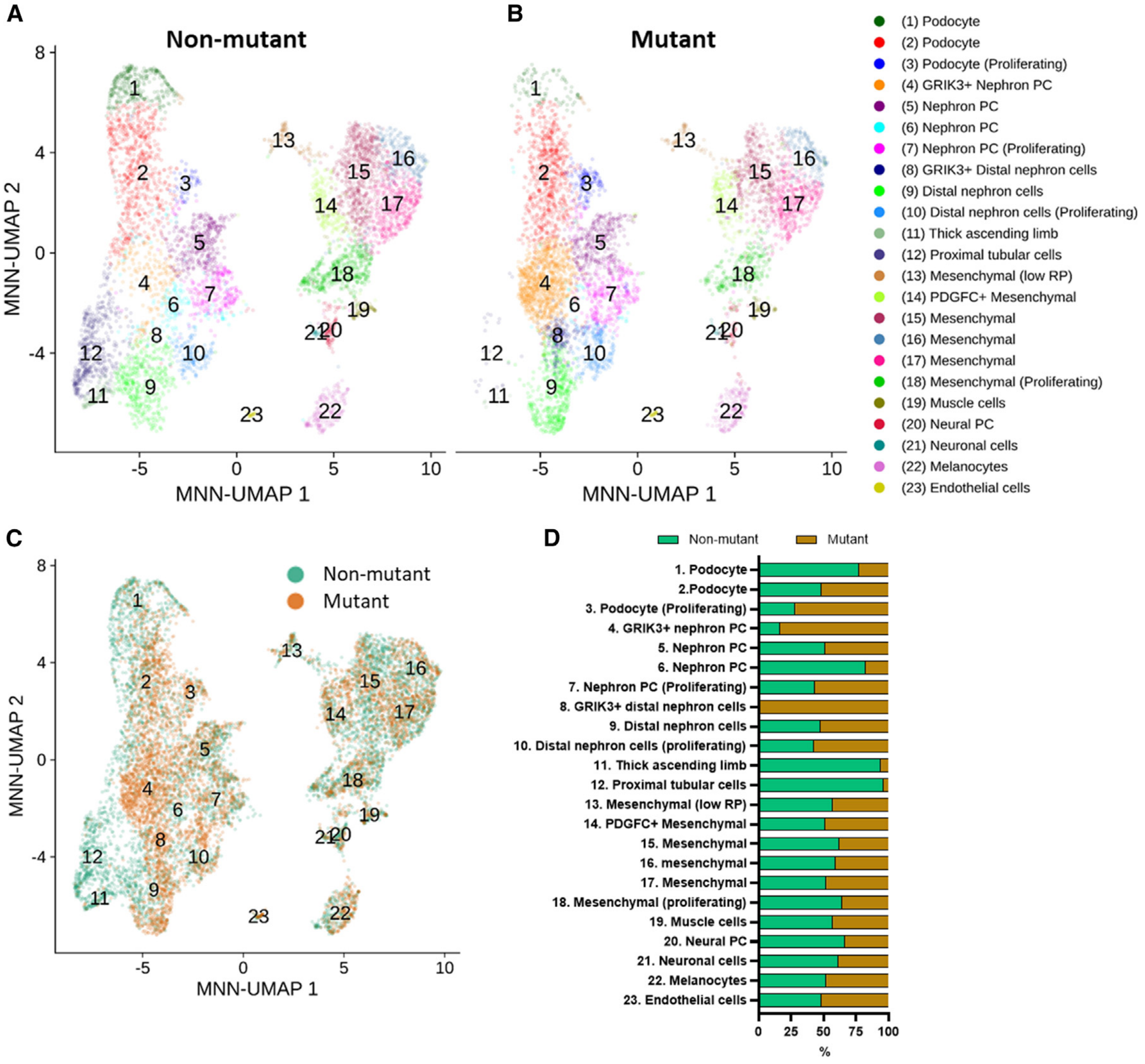

Figure 5. Single-cell RNA-seq comparison of non-mutant and mutant organoids

(A and B) MNN-corrected UMAP of cells in non-mutant (A) and mutant (B) organoids, with cell clusters numbered and highlighted in distinct colors.

(C) MNN-corrected UMAP of non-mutant and mutant cells in the same space, highlighted in different colors.

(D) The percentage of non-mutant and mutant cells in each cluster.

FREM2 mutations) where organogenesis fails to initiate (Clissold et al., 2015).

Roles for *HNF1B* in human kidney tubule morphogenesis and differentiation

The main abnormal morphological feature of heterozygous *HNF1B* mutant organoids was large-diameter multi-layered tubules rather than normal single-layered epithelial walls. This was found in both CRISPR-mutant hESC organoids and those derived from iPSCs from individuals with HNF1B-associated DKMs. Mutant organoids displayed low expression of transcripts characteristic of PTs and DTs. These included genes (e.g., *CLCN5*, *SLC34A1*, *SLC4A4*, *CLCNKA*, and *SLC12A1*) implicated in Mendelian diseases where tubules fail to function, resulting in urinary wasting of electrolytes and low-molecular-weight proteins, together with acid-base aberrations. These deficiencies help to explain the electrolyte wasting reported in *HNF1B*-associated DKMs (Adalat et al., 2019, 2009). It is unlikely that these changes result from simply absence of PTs or DTs because several other PT/DT-characteristic genes were similarly expressed in mutant and non-mutant organoids. Tubule maturation might simply be delayed in the mutant, but histological and scRNA-seq data suggest that heterozygous *HNF1B* mutations lead to the generation of aberrant nephron epithelia with features absent in healthy kidney, indicating an

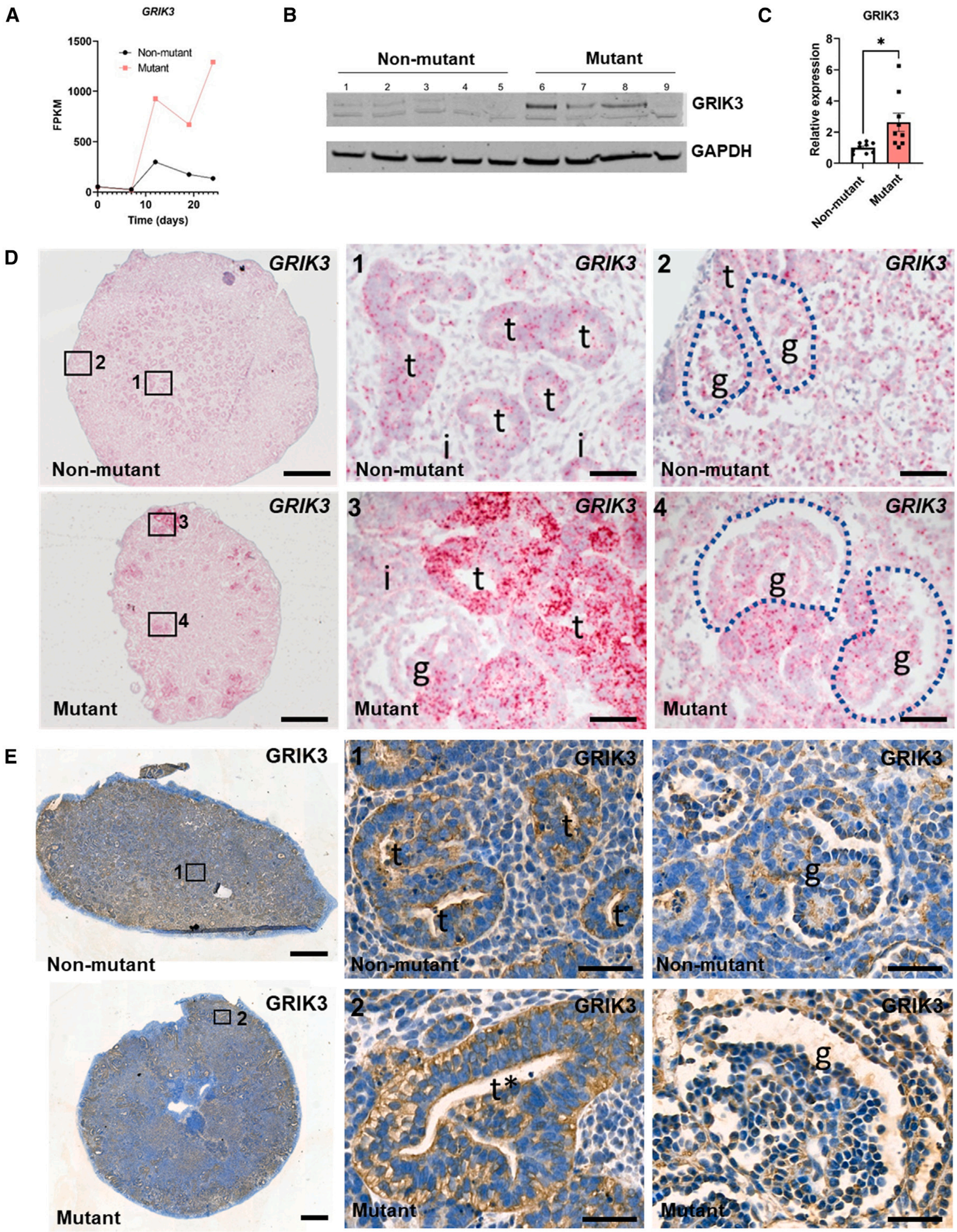

Figure 6. GRIK3 in kidney organoids

(A) RNA-seq average read counts of *GRIK3* during differentiation of non-mutant and *HNF1B* mutant hESCs, showing increased levels in mutant organoids (days 12, 19, and 25).

(B) GRIK3 western blot (5 non-mutant and 4 mutant samples).

(legend continued on next page)

abnormal cell state. These features include enlarged and hyperproliferative tubules with hybrid identities including binding to LTL and expressing CDH1, and disordered polarity evidenced by replacement of apically localized Cubilin by a diffuse cytoplasmic pattern. This is further supported by our scRNA-seq results, which demonstrate that in mutant organoids there is an almost complete lack of cells with typical PT transcriptomes and an overrepresentation of proliferative cell types in both nephron tubules and glomeruli. Our molecular analyses also suggest that *HNF1B* mutant organoids display a modest upregulation of UB/CD lineage genes (e.g., *RET* and *SCNN1G)* consistent with a metaplastic shift of tubule cell identity. It has been reported that nephron-rich hPSC-kidney organoids display a degree of plasticity such that by manipulating them biochemically they can be redirected to a UB/CD identity (Howden et al., 2021). Gene editing of hiPSCs leading to biallelic *HNF1B* deletions was reported to result in organoids that fail to form PTs (Przepiorski et al., 2018). This gross deficit resembles the severely impaired development of nephron tubules in mice carrying biallelic *Hnf1b* deletions within nephron precursor cells (Heliot et al., 2013; Massa et al., 2013). In contrast, our current human heterozygous *HNF1B* mutant model generates a more nuanced phenotype comprising aberrant structural, molecular, and functional differentiation of nephron tubule segments, rather than their absence.

Compared with the morphological and molecular derangements of tubules in our heterozygous *HNF1B* mutant organoids, mutant glomeruli had a milder phenotype with more prominent tufts. Bulk RNA-seq showed generally preserved expression of podocyte genes, while scRNA-seq showed a shift toward a proliferative mutant podocyte population. In the mutant organoids, *HNF1B* transcripts were detected in glomerular tufts but absent in non-mutant glomeruli. The latter is consistent with native human fetal kidneys where *HNF1B* is expressed by tubules but not by glomerular tuft cells.

Heterozygous *HNF1B* mutant organoids do not form cysts

HNF1B-associated DKMs can contain cysts, up to a few centimeters across (Haumaitre et al., 2006; Nakayama et al., 2023). On the other hand, a landmark clinical study reported a more complex situation (Decramer et al., 2007). Here, 18 fetuses, later found to have heterozygous *HNF1B* mutations, presented with ultrasonographically echo-bright kidneys, indicative of widespread abnormality of their internal structure. Despite this, only 11 of the 18 had overt kidney cysts. Notably, however, "… after birth, cysts appeared during the first year (17 of 18), and in patients with antenatal cysts, the number increased …." This suggests that cystogenesis in *HNF1B* mutant kidneys is a relatively late feature that long postdates a disruption of normal nephron maturation. Indeed, in our study, although mutant organoid tubules contained statistically significantly larger lumina than those of wild-type tubules, overt cysts were not present despite marked downregulation of genes (e.g., *CYS1* and *PKHD1*) that maintain a healthy non-cystic phenotype in kidney epithelia (Nakanishi et al., 2000; Yang et al., 2021). In certain genetic human cystic kidney diseases, such as autosomal dominant PKD (ADPKD) associated with *PKD1* or *PKD2* mutations, *in vivo* kidney cystogenesis is largely driven by cAMP signaling (Richards et al., 2021). Compared with control counterparts, *HNF1B* mutant organoids resisted chemical induction by cAMP which generated dilated nephrons in control organoids consistent with a lack of mature function of mutant tubules. We conclude that the genesis of nephron cysts in *HNF1B*-associated DKMs may be a secondary, and late, feature probably not driven by cAMP. Perhaps the presence of glomerular filtration in conjunction with primary aberrations in tubule biology is needed to generate overt cysts in *HNF1B* disease. Implantation of heterozygous *HNF1B* mutant organoids into mice, as described for non-mutant hPSC-kidney progenitors (Bantounas et al., 2018, 2020), will allow extended development with vascularization of glomeruli such that dilatation may then occur in glomeruli and tubules. It is also possible that, had CDs been present, cysts may have occurred. Another group generated heterozygous *HNF1B* mutant hPSCs and differentiated them into UB/CD-like cells. These cells formed UB/CD organoids, and in mutants "the number of budding regions tends to be reduced" but notably cysts were not observed (Mae et al., 2020).

(C) Quantification of B confirmed increased GRIK3/GAPDH in mutant organoids (mean ± SEM; $n = 9$, across four independent differentiation experiments; *$p < 0.05$, t test).
(D) BaseScope for *GRIK3*, signal-red dots; nuclei counterstained with hematoxylin. Left-hand images, low power overviews of day 25 organoids; other frames show high power images (1–4). In non-mutant organoids *GRIK3* was expressed in tubules (*t*) with sparser signals in interstitial cells (*i* in 1) and glomeruli (*g* in 2). In mutant organoids, *GRIK3* was highly expressed in large dysmorphic tubules (*t* in 3), with transcripts also in aberrant glomeruli (*g* in 4).
(E) GRIK3 immunostaining (brown). Left-hand images: overviews of day 25 organoids; other frames (1 and 2) are high power images. In non-mutant organoids GRIK3 was immunodetected in tubules (*t* in 1). In mutant organoids, GRIK3 was prominent in multi-layered dysplastic tubules (*t* and *asterisk* in 2). A low level of immunostaining was noted in glomeruli (*g*) of both genotypes. Bars: (D) 200 μM (left frames) and 20 μM (other frames); (E) 500 μM (left frames) and 40 μM (other frames).

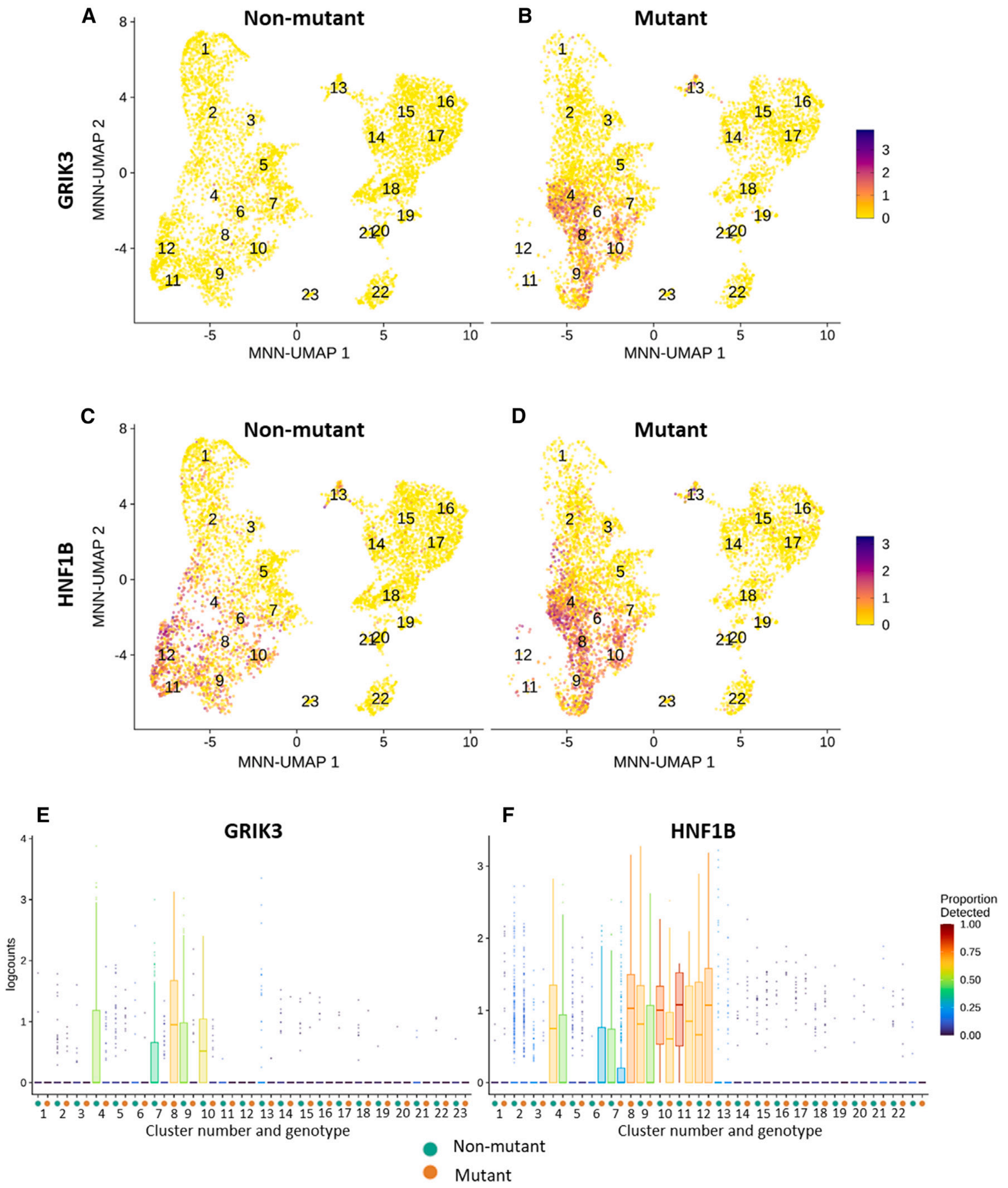

Figure 7. Localization of *GRIK3* and *HNF1B* expression in organoid cells from scRNAseq analyses
(A–D) MNN-corrected UMAP of non-mutant and mutant organoid cells, with expression of *HNF1B* and *GRIK3* highlighted, showing extensive co-expression of *GRIK3* (A, B) and *HNF1B* (C, D) in the mutant (B, D).
(E and F) Boxplot diagram quantifying the expression of *GRIK3* (E) and *HNF1B* (F) in each cell cluster of non-mutant and mutant organoids. Note that all *GRIK3*+ mutant populations also express *HNF1B*.

Molecular mechanisms of *HNF1B*-associated DKMs

RNA-seq analyses of mutant organoids identified deregulation of many genes containing the canonical HNF binding sequence (Adalat et al., 2009). These included genes expressed by kidney tubule epithelia, e.g., *CLCN5*, *CYS1*, *HNF1A*, *HNF4A*, *KCNJ16*, *MUC1*, *PKHD1*, and *SLC34A1*, likely a direct effect of decreased functional HNF1B protein. Several key transcripts involved in kidney development,

e.g., WNT pathway, were also deregulated yet do not contain the canonical HNF1B binding site. These changes may be secondary to direct HNF1B-induced gene regulation, but their altered expression may contribute to the DKM-like phenotype. A prominent example of such genes was the glutamate receptor subunit *GRIK3* that was markedly upregulated in heterozygous *HNF1B* mutant organoid differentiation. Moreover, scRNA-seq analyses showed that this upregulation occurred in cells high in *HNF1B*. In support of a role for glutamate receptors in disease, KEGG pathway analysis predicted deregulation of glutamatergic signaling, while, histologically, *GRIK3* transcripts and protein are noticeably higher in dysmorphic tubules of *HNF1B* mutant organoids and in mutant over control fetal kidney sections. Moreover, several glutamate receptor genes, or components of glutamatergic intracellular signaling machinery, were deregulated in mutant organoids. These observations are highly novel given that glutamate receptors have hitherto not been studied in either normal or abnormal human kidney development.

Glutamatergic signaling and glutamate metabolism have been intensively studied in neuronal tissues where glutamate acts as an excitatory neurotransmitter (Hansen et al., 2021). Roles for glutamatergic signaling, and GRIK3 itself, are emerging outside the nervous system. For instance, GRIK3 expression is prominent in breast cancer tissues, and it increases proliferation and migration of breast cancer cells *in vitro*, driving epithelial-to-mesenchymal transition (Xiao et al., 2019). GRIK3 is implicated in proliferation and migration of intestinal and lung cancer cells (Du et al., 2020). Of note, components of the glutamate signaling system have been identified in mature kidneys *in vivo* and in kidney epithelial cell lines (Hediger, 1999; Valdivielso et al., 2020), e.g., *GRIN2A* and *GRIN2B* in murine kidney tubules (Iwata et al., 2022) with *SLC7A11* and *SLC1A* in PT cells (Shayakul et al., 1997; Wang et al., 2021; Welbourne and Matthews, 1999). Increased monosodium glutamate dietary intake in rats leads to increased glomerular filtration rate, which the NMDA receptor antagonist MK-801 reduced (Mahieu et al., 2016). Glutamate transporters move extracellular glutamate into PTs, facilitating glutamine/glutamate metabolism, urinary acidification, and movement of bicarbonate back into the body (Welbourne and Matthews, 1999). Knockdown of *GRIN1* (NMDAR1) in a PT cell line led to an epithelial-to-mesenchymal transition, while addition of NMDA blunted *in vitro* expression of transforming growth factor β1 (TGF-β1)-induced mesenchymal markers (Bozic et al., 2011). Moreover, NMDA administration ameliorated kidney fibrosis triggered by ureteric obstruction (Bozic et al., 2011). In mice receiving chemical NMDAR blockade, urinary protein levels rise while, *in vitro*, antagonizing NMDAR causes cytoskeletal remodeling in podocytes (Giardino et al., 2009). These observations indicate that glutamate and glutamatergic signaling impact on the health of kidney epithelial cells. They are also consistent with the hypothesis that the deregulation of glutamatergic signaling genes such as *GRIK3* plays roles in the pathobiology of kidney dysplasia associated with *HNF1B* mutation.

An unanswered question is whether developing kidneys are exposed to glutamine/glutamate *in vivo*. Amino acids are present in the milieu of early developing embryos (Van Winkle, 2021), and human cord blood at term contains glutamate, which increases with fetal distress (Perez-Mato et al., 2016). In the central nervous system, astrocytes release glutamine that is taken up by neurons to generate glutamate for use in neurotransmission (Andersen and Schousboe, 2022). Whether a similar glutamate-producing mechanism operates in the kidney is unknown. Alternatively, kidney glutamate receptors may function to sense other amino acids in the proto-urine: for example, D-serine activates NMDARs in the kidney resulting in Ca^{2+}-mediated increase in reactive oxygen species, leading to renal insufficiency in mice. Many of the resulting symptoms are reversed by NMDAR inhibitors (Tseng et al., 2021). Finally, drugs that modulate glutamate signaling are being explored as treatments in non-renal (e.g., brain) diseases (Stone, 2011), and this suggests that similar drugs may ameliorate features of kidney organogenesis associated with *HNF1B* mutations.

EXPERIMENTAL PROCEDURES

Resource availability

Lead contact

Susan J. Kimber (sue.kimber@manchester.ac.uk)

Materials availability

Materials generated in this study are available from the Kimber lab upon request.

Data and code availability

Generated RNA-seq and scRNA-seq datasets can be found at ArrayExpress under the following accession numbers: ArrayExpress: E-MTAB-12824 (RNA-seq of hESC/organoids), ArrayExpress: E-MTAB-12822 (RNA-seq of native kidney), and ArrayExpress: E-MTAB-13500 (scRNA-seq).

hPSC cell culture

See supplemental experimental procedures.

CRIPSR-Cas9^n editing of hESCs

Two artificially created inserts for gRNAs, targeting *HNF1B* at positions 231 of the coding strand and 171 of the complementary strand, and containing the appropriate overhangs, were cloned into BbsI-digested pX461 plasmid (Addgene, #48140), which also expresses the nickase (D10A) version of Cas9 (Cas9^n) and a GFP tag. The resultant plasmids were then both transfected into MAN13 hESCs using a Lonza 4D-Nucleofector and the Amaxa P3 Primary Cell 4D-Nucleofector X Kit L according to the manufacturer's instructions. Transfected cells were identified and sorted

by GFP fluorescence at 5-10 × 10^3 cells per well in 6-well plates for up to 15 days, and then each was transferred into separate wells and expanded. Edited clones were identified by sequencing. (See supplemental experimental procedures).

iPSC derivation

PBMCs were isolated by gradient centrifugation on Ficoll and expanded before being transduced with the CytoTune-iPS 2.0 Sendai Reprogramming Kit (A16517, Thermo Fisher Scientific), according to the manufacturer's instructions. See also supplemental experimental procedures.

2D and organoid differentiation

2D and organoid differentiation was performed by an adaptation of previously described protocols as described in supplemental experimental procedures.

Next-generation RNA-seq and scRNA-seq

Both RNA-seq and scRNA-seq were performed by the University of Manchester Genomics Facility. Detailed methods are in supplementary experimental procedures.

Immunological methods

Western blotting, immunocytochemistry of 2D cultures, and immunohistochemistry of paraffin-embedded, sectioned organoid, and human tissues were performed using standard protocols (see supplemental experimental procedures).

In situ RNA hybridization (BaseScope)

Organoids were fixed in 4% paraformaldehyde, paraffin embedded, and sectioned at 5 μm. BaseScope ISH (ACDBio, Newark, CA, USA) was conducted following manufacturer's instructions, using the BaseScope detection reagent Kit v2-RED. See also supplemental experimental procedures.

cAMP-induced tubule dilatation and cell proliferation assay

See supplemental experimental procedures.

Bioinformatics analyses

GO and KEGG pathway analyses, as well as *in silico* identification of promoters directly bound by HNF1B, are described in supplemental experimental procedures.

Quantification and statistical analysis

Detailed statistical methods for each experiment are given in supplementary experimental procedures.

SUPPLEMENTAL INFORMATION

Supplemental information can be found online at https://doi.org/10.1016/j.stemcr.2024.04.011.

ACKNOWLEDGMENTS

We acknowledge research support from: Kidney Research UK project grant JFS/RP/008/20160916 (S.J.K., A.S.W., and I.B.); Medical Research Council project grant MR/T016809/1 (A.S.W. and F.M.L.); Engineering and Physical Sciences Research Council (EPSRC)/Medical Research Council (MRC) Centre for Doctoral Training grant EP/L014904/1(K.M.R.); European Union (SYBIL European Community's Seventh Framework Program, FP7/2007-2013, 602300 [S.J.K., S.W.]); Wellcome Leap Human Organs Physiology and Engineering (HOPE) Initiative (A.S.W., S.J.K., and I.B.); and Kidneys for Life pump priming projects 2021 (A.S.W., S.J.K., I.B., and K.M.R.). Support is also acknowledged from the Malaysian Ministry of Higher Education, International Islamic University Malaysia (F.T.), and an Erasmus Scholarship (S.H.).

AUTHOR CONTRIBUTIONS

SJK, ASW, IB and KMR designed the studies, supervised the project and drafted the paper. IB, KMR, FT, FML, SW, NB, LW and SH undertook laboratory experiments. SC provided kidneys for histology. KAH and ASW assessed the *HNF1B* family and sourced samples to generate hiPSCs. LAHZ, I-HL and SYK undertook bioinformatic analyses. All authors discussed the results and commented on the manuscript.

DECLARATION OF INTERESTS

The authors declare no conflicts of interest.

Received: February 14, 2024
Revised: April 23, 2024
Accepted: April 24, 2024
Published: May 23, 2024

REFERENCES

Adalat, S., Hayes, W.N., Bryant, W.A., Booth, J., Woolf, A.S., Kleta, R., Subtil, S., Clissold, R., Colclough, K., Ellard, S., and Bockenhauer, D. (2019). HNF1B Mutations Are Associated With a Gitelman-like Tubulopathy That Develops During Childhood. Kidney Int. Rep. *4*, 1304–1311. https://doi.org/10.1016/j.ekir.2019.05.019.

Adalat, S., Woolf, A.S., Johnstone, K.A., Wirsing, A., Harries, L.W., Long, D.A., Hennekam, R.C., Ledermann, S.E., Rees, L., van't Hoff, W., et al. (2009). HNF1B mutations associate with hypomagnesemia and renal magnesium wasting. J. Am. Soc. Nephrol. *20*, 1123–1131. https://doi.org/10.1681/ASN.2008060633.

Anders, C., Ashton, N., Ranjzad, P., Dilworth, M.R., and Woolf, A.S. (2013). Ex vivo modeling of chemical synergy in prenatal kidney cystogenesis. PLoS One *8*, e57797. https://doi.org/10.1371/journal.pone.0057797.

Andersen, J.V., and Schousboe, A. (2023). Glial Glutamine Homeostasis in Health and Disease. Neurochem. Res. *48*, 1100–1128. https://doi.org/10.1007/s11064-022-03771-1.

Bantounas, I., Lopes, F.M., Rooney, K.M., Woolf, A.S., and Kimber, S.J. (2021). The miR-199a/214 Cluster Controls Nephrogenesis and Vascularization in a Human Embryonic Stem Cell Model. Stem Cell Rep. *16*, 134–148. https://doi.org/10.1016/j.stemcr.2020.11.007.

Bantounas, I., Ranjzad, P., Tengku, F., Silajdžić, E., Forster, D., Asselin, M.C., Lewis, P., Lennon, R., Plagge, A., Wang, Q., et al. (2018).

Generation of Functioning Nephrons by Implanting Human Pluripotent Stem Cell-Derived Kidney Progenitors. Stem Cell Rep. *10*, 766–779. https://doi.org/10.1016/j.stemcr.2018.01.008.

Bantounas, I., Silajdžić, E., Woolf, A.S., and Kimber, S.J. (2020). Formation of Mature Nephrons by Implantation of Human Pluripotent Stem Cell-Derived Progenitors into Mice. Methods Mol. Biol. *2067*, 309–322. https://doi.org/10.1007/978-1-4939-9841-8_19.

Bozic, M., de Rooij, J., Parisi, E., Ortega, M.R., Fernandez, E., and Valdivielso, J.M. (2011). Glutamatergic signaling maintains the epithelial phenotype of proximal tubular cells. J. Am. Soc. Nephrol. *22*, 1099–1111. https://doi.org/10.1681/ASN.2010070701.

Casemayou, A., Fournel, A., Bagattin, A., Schanstra, J., Belliere, J., Decramer, S., Marsal, D., Gillet, M., Chassaing, N., Huart, A., et al. (2017). Hepatocyte Nuclear Factor-1beta Controls Mitochondrial Respiration in Renal Tubular Cells. J. Am. Soc. Nephrol. *28*, 3205–3217. https://doi.org/10.1681/ASN.2016050508.

Chan, S.C., Hajarnis, S.S., Vrba, S.M., Patel, V., and Igarashi, P. (2020). Hepatocyte nuclear factor 1β suppresses canonical Wnt signaling through transcriptional repression of lymphoid enhancer-binding factor 1. J. Biol. Chem. *295*, 17560–17572. https://doi.org/10.1074/jbc.RA120.015592.

Chan, S.C., Zhang, Y., Shao, A., Avdulov, S., Herrera, J., Aboudehen, K., Pontoglio, M., and Igarashi, P. (2018). Mechanism of Fibrosis in HNF1B-Related Autosomal Dominant Tubulointerstitial Kidney Disease. J. Am. Soc. Nephrol. *29*, 2493–2509. https://doi.org/10.1681/ASN.2018040437.

Clissold, R.L., Hamilton, A.J., Hattersley, A.T., Ellard, S., and Bingham, C. (2015). HNF1B-associated renal and extra-renal disease-an expanding clinical spectrum. Nat. Rev. Nephrol. *11*, 102–112. https://doi.org/10.1038/nrneph.2014.232.

Decramer, S., Parant, O., Beaufils, S., Clauin, S., Guillou, C., Kessler, S., Aziza, J., Bandin, F., Schanstra, J.P., and Bellanné-Chantelot, C. (2007). Anomalies of the TCF2 gene are the main cause of fetal bilateral hyperechogenic kidneys. J. Am. Soc. Nephrol. *18*, 923–933. https://doi.org/10.1681/ASN.2006091057.

Desgrange, A., Heliot, C., Skovorodkin, I., Akram, S.U., Heikkilä, J., Ronkainen, V.P., Miinalainen, I., Vainio, S.J., and Cereghini, S. (2017). HNF1B controls epithelial organization and cell polarity during ureteric bud branching and collecting duct morphogenesis. Development *144*, 4704–4719. https://doi.org/10.1242/dev.154336.

Du, H., He, Z., Feng, F., Chen, D., Zhang, L., Bai, J., Wu, H., Han, E., and Zhang, J. (2020). Hsa_circ_0038646 promotes cell proliferation and migration in colorectal cancer via miR-331-3p/GRIK3. Oncol. Lett. *20*, 266–274. https://doi.org/10.3892/ol.2020.11547.

Fiorentino, A., Christophorou, A., Massa, F., Garbay, S., Chiral, M., Ramsing, M., Rasmussen, M., Gubler, M.C., Bessieres, B., Heidet, L., et al. (2020). Developmental Renal Glomerular Defects at the Origin of Glomerulocystic Disease. Cell Rep. *33*, 108304. https://doi.org/10.1016/j.celrep.2020.108304.

Giardino, L., Armelloni, S., Corbelli, A., Mattinzoli, D., Zennaro, C., Guerrot, D., Tourrel, F., Ikehata, M., Li, M., Berra, S., et al. (2009). Podocyte glutamatergic signaling contributes to the function of the glomerular filtration barrier. J. Am. Soc. Nephrol. *20*, 1929–1940. https://doi.org/10.1681/ASN.2008121286.

Grand, K., Stoltz, M., Rizzo, L., Röck, R., Kaminski, M.M., Salinas, G., Getwan, M., Naert, T., Pichler, R., and Lienkamp, S.S. (2023). HNF1B Alters an Evolutionarily Conserved Nephrogenic Program of Target Genes. J. Am. Soc. Nephrol. *34*, 412–432. https://doi.org/10.1681/ASN.2022010076.

Gresh, L., Fischer, E., Reimann, A., Tanguy, M., Garbay, S., Shao, X., Hiesberger, T., Fiette, L., Igarashi, P., Yaniv, M., and Pontoglio, M. (2004). A transcriptional network in polycystic kidney disease. EMBO J. *23*, 1657–1668. https://doi.org/10.1038/sj.emboj.7600160.

Hansen, K.B., Wollmuth, L.P., Bowie, D., Furukawa, H., Menniti, F.S., Sobolevsky, A.I., Swanson, G.T., Swanger, S.A., Greger, I.H., Nakagawa, T., et al. (2021). Structure, Function, and Pharmacology of Glutamate Receptor Ion Channels. Pharmacol. Rev. *73*, 1469–1658. https://doi.org/10.1124/pharmrev.120.000131.

Haumaitre, C., Fabre, M., Cormier, S., Baumann, C., Delezoide, A.L., and Cereghini, S. (2006). Severe pancreas hypoplasia and multicystic renal dysplasia in two human fetuses carrying novel HNF1beta/MODY5 mutations. Hum. Mol. Genet. *15*, 2363–2375. https://doi.org/10.1093/hmg/ddl161.

Hediger, M.A. (1999). Glutamate transporters in kidney and brain. Am. J. Physiol. *277*, F487–F492. https://doi.org/10.1152/ajprenal.1999.277.4.F487.

Heliot, C., Desgrange, A., Buisson, I., Prunskaite-Hyyryläinen, R., Shan, J., Vainio, S., Umbhauer, M., and Cereghini, S. (2013). HNF1B controls proximal-intermediate nephron segment identity in vertebrates by regulating Notch signalling components and Irx1/2. Development *140*, 873–885. https://doi.org/10.1242/dev.086538.

Howden, S.E., Wilson, S.B., Groenewegen, E., Starks, L., Forbes, T.A., Tan, K.S., Vanslambrouck, J.M., Holloway, E.M., Chen, Y.H., Jain, S., et al. (2021). Plasticity of distal nephron epithelia from human kidney organoids enables the induction of ureteric tip and stalk. Cell Stem Cell *28*, 671–684.e6. https://doi.org/10.1016/j.stem.2020.12.001.

Iwata, Y., Nakade, Y., Kitajima, S., Yoneda-Nakagawa, S., Oshima, M., Sakai, N., Ogura, H., Sato, K., Toyama, T., Yamamura, Y., et al. (2022). Protective effect of d-alanine against acute kidney injury. Am. J. Physiol. Renal Physiol. *322*, F667–F679. https://doi.org/10.1152/ajprenal.00198.2021.

Jafree, D.J., Moulding, D., Kolatsi-Joannou, M., Perretta Tejedor, N., Price, K.L., Milmoe, N.J., Walsh, C.L., Correra, R.M., Winyard, P.J., Harris, P.C., et al. (2019). Spatiotemporal dynamics and heterogeneity of renal lymphatics in mammalian development and cystic kidney disease. Elife *8*, e48183. https://doi.org/10.7554/eLife.48183.

Kishi, S., Brooks, C.R., Taguchi, K., Ichimura, T., Mori, Y., Akinfolarin, A., Gupta, N., Galichon, P., Elias, B.C., Suzuki, T., et al. (2019). Proximal tubule ATR regulates DNA repair to prevent maladaptive renal injury responses. J. Clin. Invest. *129*, 4797–4816. https://doi.org/10.1172/JCI122313.

Kohl, S., Avni, F.E., Boor, P., Capone, V., Clapp, W.L., De Palma, D., Harris, T., Heidet, L., Hilger, A.C., Liapis, H., et al. (2022). Definition, diagnosis and clinical management of non-obstructive kidney dysplasia: a consensus statement by the ERKNet Working

Group on Kidney Malformations. Nephrol. Dial. Transplant. *37*, 2351–2362. https://doi.org/10.1093/ndt/gfac207.

Kolatsi-Joannou, M., Bingham, C., Ellard, S., Bulman, M.P., Allen, L.I.S., Hattersley, A.T., and Woolf, A.S. (2001). Hepatocyte nuclear factor-1beta: a new kindred with renal cysts and diabetes and gene expression in normal human development. J. Am. Soc. Nephrol. *12*, 2175–2180. https://doi.org/10.1681/ASN.V12102175.

Lim, S.H., Kim, J.H., Han, K.H., Ahn, Y.H., Kang, H.G., Ha, I.S., and Cheong, H.I. (2020). Genotype and Phenotype Analyses in Pediatric Patients with HNF1B Mutations. J. Clin. Med. *9*, 2320. https://doi.org/10.3390/jcm9072320.

Lindstrom, N.O., Tran, T., Guo, J., Rutledge, E., Parvez, R.K., Thornton, M.E., Grubbs, B., McMahon, J.A., and McMahon, A.P. (2018). Conserved and Divergent Molecular and Anatomic Features of Human and Mouse Nephron Patterning. J. Am. Soc. Nephrol. *29*, 825–840. https://doi.org/10.1681/ASN.2017091036.

Lokmane, L., Heliot, C., Garcia-Villalba, P., Fabre, M., and Cereghini, S. (2010). vHNF1 functions in distinct regulatory circuits to control ureteric bud branching and early nephrogenesis. Development *137*, 347–357. https://doi.org/10.1242/dev.042226.

Mae, S.I., Ryosaka, M., Sakamoto, S., Matsuse, K., Nozaki, A., Igami, M., Kabai, R., Watanabe, A., and Osafune, K. (2020). Expansion of Human iPSC-Derived Ureteric Bud Organoids with Repeated Branching Potential. Cell Rep. *32*, 107963. https://doi.org/10.1016/j.celrep.2020.107963.

Mahieu, S., Klug, M., Millen, N., Fabro, A., Benmelej, A., and Contini, M.D.C. (2016). Monosodium glutamate intake affect the function of the kidney through NMDA receptor. Life Sci. *149*, 114–119. https://doi.org/10.1016/j.lfs.2016.02.023.

Massa, F., Garbay, S., Bouvier, R., Sugitani, Y., Noda, T., Gubler, M.C., Heidet, L., Pontoglio, M., and Fischer, E. (2013). Hepatocyte nuclear factor 1beta controls nephron tubular development. Development *140*, 886–896. https://doi.org/10.1242/dev.086546.

McMahon, A.P. (2016). Development of the Mammalian Kidney. Curr. Top. Dev. Biol. *117*, 31–64. https://doi.org/10.1016/bs.ctdb.2015.10.010.

Nakanishi, K., Sweeney, W.E., Jr., Zerres, K., Guay-Woodford, L.M., and Avner, E.D. (2000). Proximal tubular cysts in fetal human autosomal recessive polycystic kidney disease. J. Am. Soc. Nephrol. *11*, 760–763. https://doi.org/10.1681/ASN.V114760.

Nakayama, Y., Sawa, N., Suwabe, T., Yamanouchi, M., Ikuma, D., Mizuno, H., Hasegawa, E., Hoshino, J., Sekine, A., Oba, Y., et al. (2023). Kidney Histology Findings in a Patient with Autosomal Dominant Tubulointerstitial Kidney Disease Subtype Hepatocyte Nuclear Factor 1beta. Intern. Med. *62*, 419–422. https://doi.org/10.2169/internalmedicine.9364-22.

Niborski, L.L., Paces-Fessy, M., Ricci, P., Bourgeois, A., Magalhães, P., Kuzma-Kuzniarska, M., Lesaulnier, C., Reczko, M., Declercq, E., Zürbig, P., et al. (2021). Hnf1b haploinsufficiency differentially affects developmental target genes in a new renal cysts and diabetes mouse model. Dis. Model. Mech. *14*, dmm047498. https://doi.org/10.1242/dmm.047498.

Nielsen, R., Christensen, E.I., and Birn, H. (2016). Megalin and cubilin in proximal tubule protein reabsorption: from experimental models to human disease. Kidney Int. *89*, 58–67. https://doi.org/10.1016/j.kint.2015.11.007.

Nouwen, E.J., Dauwe, S., van der Biest, I., and De Broe, M.E. (1993). Stage- and segment-specific expression of cell-adhesion molecules N-CAM, A-CAM, and L-CAM in the kidney. Kidney Int. *44*, 147–158. https://doi.org/10.1038/ki.1993.225.

Perez-Mato, M., Iglesias-Deus, A., Rujido, S., da Silva-Candal, A., Sobrino, T., Couce, M.L., Fraga, J.M., Castillo, J., and Campos, F. (2016). Potential protective role of endogenous glutamate-oxaloacetate transaminase against glutamate excitotoxicity in fetal hypoxic-ischaemic asphyxia. Dev. Med. Child Neurol. *58*, 57–62. https://doi.org/10.1111/dmcn.12851.

Piedrafita, A., Balayssac, S., Casemayou, A., Saulnier-Blache, J.S., Lucas, A., Iacovoni, J.S., Breuil, B., Chauveau, D., Decramer, S., Malet-Martino, M., et al. (2021). Hepatocyte nuclear factor-1beta shapes the energetic homeostasis of kidney tubule cells. FASEB J. *35*, e21931. https://doi.org/10.1096/fj.202100782RR.

Przepiorski, A., Sander, V., Tran, T., Hollywood, J.A., Sorrenson, B., Shih, J.H., Wolvetang, E.J., McMahon, A.P., Holm, T.M., and Davidson, A.J. (2018). A Simple Bioreactor-Based Method to Generate Kidney Organoids from Pluripotent Stem Cells. Stem Cell Rep. *11*, 470–484. https://doi.org/10.1016/j.stemcr.2018.06.018.

Richards, T., Modarage, K., Malik, S.A., and Goggolidou, P. (2021). The cellular pathways and potential therapeutics of Polycystic Kidney Disease. Biochem. Soc. Trans. *49*, 1171–1188. https://doi.org/10.1042/BST20200757.

Rooney, K.M., Woolf, A.S., and Kimber, S.J. (2021). Towards Modelling Genetic Kidney Diseases with Human Pluripotent Stem Cells. Nephron *145*, 285–296. https://doi.org/10.1159/000514018.

Shayakul, C., Kanai, Y., Lee, W.S., Brown, D., Rothstein, J.D., and Hediger, M.A. (1997). Localization of the high-affinity glutamate transporter EAAC1 in rat kidney. Am. J. Physiol. *273*, F1023–F1029. https://doi.org/10.1152/ajprenal.1997.273.6.F1023.

Stone, J.M. (2011). Glutamatergic antipsychotic drugs: a new dawn in the treatment of schizophrenia? Ther. Adv. Psychopharmacol. *1*, 5–18. https://doi.org/10.1177/2045125311400779.

Taguchi, A., Kaku, Y., Ohmori, T., Sharmin, S., Ogawa, M., Sasaki, H., and Nishinakamura, R. (2014). Redefining the in vivo origin of metanephric nephron progenitors enables generation of complex kidney structures from pluripotent stem cells. Cell Stem Cell *14*, 53–67. https://doi.org/10.1016/j.stem.2013.11.010.

Takasato, M., Er, P.X., Chiu, H.S., Maier, B., Baillie, G.J., Ferguson, C., Parton, R.G., Wolvetang, E.J., Roost, M.S., Chuva de Sousa Lopes, S.M., and Little, M.H. (2015). Kidney organoids from human iPS cells contain multiple lineages and model human nephrogenesis. Nature *526*, 564–568. https://doi.org/10.1038/nature15695.

Tseng, Y.S., Liao, C.H., Wu, W.B., and Ma, M.C. (2021). N-methyl-d-aspartate receptor hyperfunction contributes to d-serine-mediated renal insufficiency. Am. J. Physiol. Renal Physiol. *320*, F799–F813. https://doi.org/10.1152/ajprenal.00461.2020.

Valdivielso, J.M., Eritja, À., Caus, M., and Bozic, M. (2020). Glutamate-Gated NMDA Receptors: Insights into the Function and Signaling in the Kidney. Biomolecules *10*, 1051. https://doi.org/10.3390/biom10071051.

Van Winkle, L.J. (2021). Amino Acid Transport and Metabolism Regulate Early Embryo Development: Species Differences, Clinical Significance, and Evolutionary Implications. Cells *10*, 3154. https://doi.org/10.3390/cells10113154.

Verdeguer, F., Le Corre, S., Fischer, E., Callens, C., Garbay, S., Doyen, A., Igarashi, P., Terzi, F., and Pontoglio, M. (2010). A mitotic transcriptional switch in polycystic kidney disease. Nat. Med. *16*, 106–110. https://doi.org/10.1038/nm.2068.

Wang, Y., Quan, F., Cao, Q., Lin, Y., Yue, C., Bi, R., Cui, X., Yang, H., Yang, Y., Birnbaumer, L., et al. (2021). Quercetin alleviates acute kidney injury by inhibiting ferroptosis. J. Adv. Res. *28*, 231–243. https://doi.org/10.1016/j.jare.2020.07.007.

Welbourne, T.C., and Matthews, J.C. (1999). Glutamate transport and renal function. Am. J. Physiol. *277*, F501–F505. https://doi.org/10.1152/ajprenal.1999.277.4.F501.

Wilson, S.B., and Little, M.H. (2021). The origin and role of the renal stroma. Development *148*, dev199886. https://doi.org/10.1242/dev.199886.

Winyard, P.J., Nauta, J., Lirenman, D.S., Hardman, P., Sams, V.R., Risdon, R.A., and Woolf, A.S. (1996a). Deregulation of cell survival in cystic and dysplastic renal development. Kidney Int. *49*, 135–146. https://doi.org/10.1038/ki.1996.18.

Winyard, P.J., Risdon, R.A., Sams, V.R., Dressler, G.R., and Woolf, A.S. (1996b). The PAX2 tanscription factor is expressed in cystic and hyperproliferative dysplastic epithelia in human kidney malformations. J. Clin. Invest. *98*, 451–459. https://doi.org/10.1172/jci118811.

Woolf, A.S. (2019). Growing a new human kidney. Kidney Int. *96*, 871–882. https://doi.org/10.1016/j.kint.2019.04.040.

Wu, H., Uchimura, K., Donnelly, E.L., Kirita, Y., Morris, S.A., and Humphreys, B.D. (2018). Comparative Analysis and Refinement of Human PSC-Derived Kidney Organoid Differentiation with Single-Cell Transcriptomics. Cell Stem Cell *23*, 869–881.e8. https://doi.org/10.1016/j.stem.2018.10.010.

Xiao, B., Kuang, Z., Zhang, W., Hang, J., Chen, L., Lei, T., He, Y., Deng, C., Li, W., Lu, J., et al. (2019). Glutamate Ionotropic Receptor Kainate Type Subunit 3 (GRIK3) promotes epithelial-mesenchymal transition in breast cancer cells by regulating SPDEF/CDH1 signaling. Mol. Carcinog. *58*, 1314–1323. https://doi.org/10.1002/mc.23014.

Yang, C., Harafuji, N., O'Connor, A.K., Kesterson, R.A., Watts, J.A., Majmundar, A.J., Braun, D.A., Lek, M., Laricchia, K.M., Fathy, H.M., et al. (2021). Cystin genetic variants cause autosomal recessive polycystic kidney disease associated with altered Myc expression. Sci. Rep. *11*, 18274. https://doi.org/10.1038/s41598-021-97046-4.

Stem Cell Reports, Volume *19*

Supplemental Information

Human pluripotent stem cell-derived kidney organoids reveal tubular epithelial pathobiology of heterozygous *HNF1B*-associated dysplastic kidney malformations

Ioannis Bantounas, Kirsty M. Rooney, Filipa M. Lopes, Faris Tengku, Steven Woods, Leo A.H. Zeef, I-Hsuan Lin, Shweta Y. Kuba, Nicola Bates, Sandra Hummelgaard, Katherine A. Hillman, Silvia Cereghini, Adrian S. Woolf, and Susan J. Kimber

Figure S1

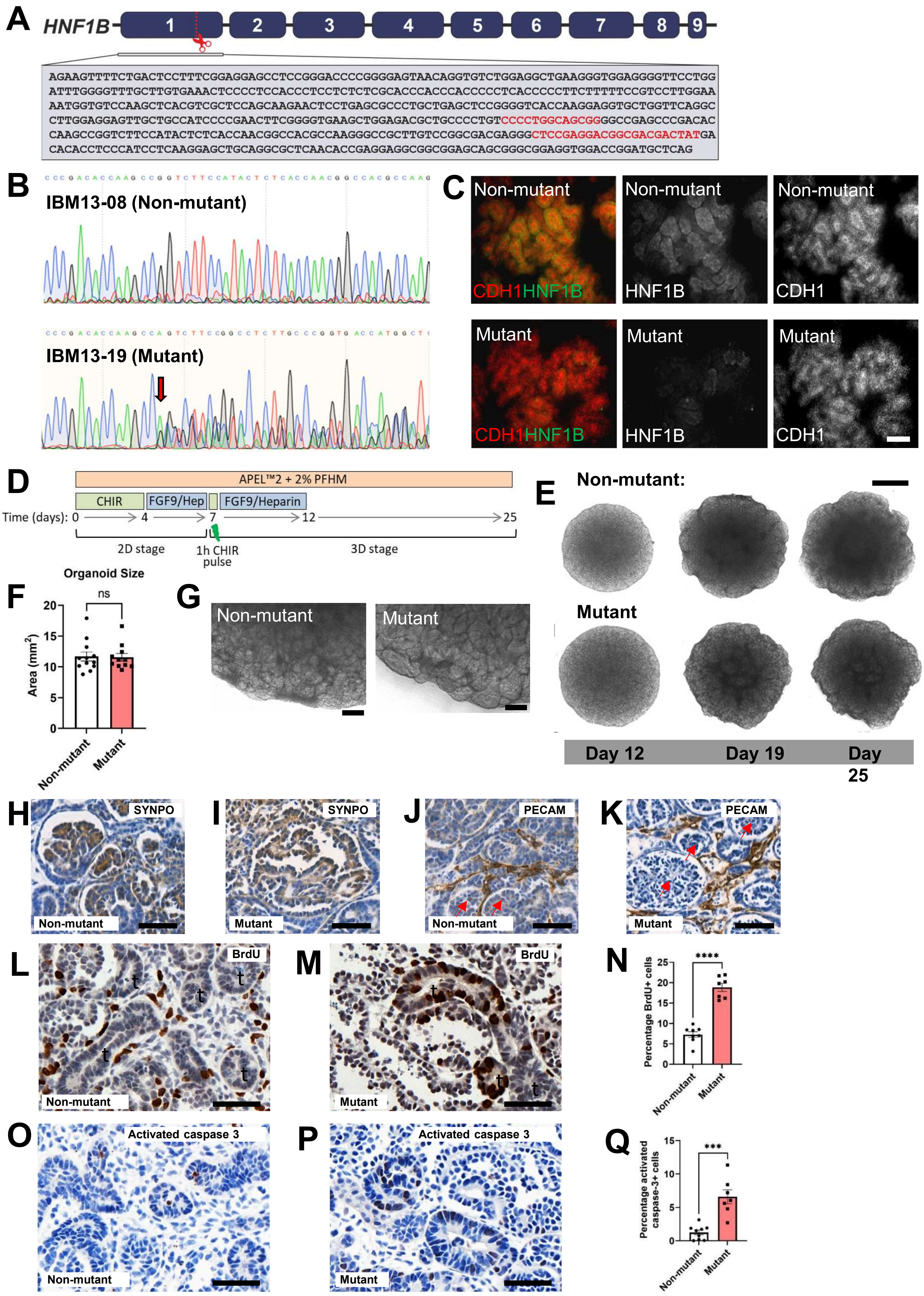

A
HNF1B
1
2
3
4
5
6
7
8
9
AGAAGTTTTCTGACTCCTTTCGGAGGAGCCTCCGGGACCCCGGGGAGTAACAGGTGTCTGGAGGCTGAAGGGTGGAGGGGTTCCTGG
ATTTGGGGTTTGCTTGTGAAACTCCCCTCCACCCTCCTCTCTCGCACCCACCCACCCCCTCACCCCCTTCTTTTTCCGTCCTTGGAA
AATGGTGTCCAAGCTCACGTCGCTCCAGCAAGAACTCCTGAGCGCCCTGCTGAGCTCCGGGGTCACCAAGGAGGTGCTGGTTCAGGC
CTTGGAGGAGTTGCTGCCATCCCCGAACTTCGGGGTGAAGCTGGAGACGCTGCCCCTGTCCCCTGGCAGCGGGGCCGAGCCCGACAC
CAAGCCGGTCTTCCATACTCTCACCAACGGCCACGCCAAGGGCCGCTTGTCCGGCGACGAGGGCTCCGAGGACGGCGACGACTATGA
CACACCTCCCATCCTCAAGGAGCTGCAGGCGCTCAACACCGAGGAGGCGGCGGAGCAGCGGGCGGAGGTGGACCGGATGCTCAG
B
IBM13-08 (Non-mutant)
IBM13-19 (Mutant)
C
Non-mutant
CDH1HNF1B
HNF1B
CDH1
Mutant
D
APEL™2 + 2% PFHM
CHIR
FGF9/Hep
FGF9/Heparin
Time (days): 0
4
7
12
25
2D stage
1h CHIR pulse
3D stage
E
Non-mutant:
Mutant
Day 12
Day 19
Day 25
F
Organoid Size
ns
Area (mm2)
0
5
10
15
20
Non-mutant
Mutant
G
Non-mutant
Mutant
H
SYNPO
Non-mutant
I
SYNPO
Mutant
J
PECAM
Non-mutant
K
PECAM
Mutant
L
BrdU
Non-mutant
t
M
BrdU
Mutant
N
Percentage BrdU+ cells
****
0
5
10
15
20
25
Non-mutant
Mutant
O
Activated caspase 3
Non-mutant
P
Activated caspase 3
Mutant
Q
Percentage activated caspase-3+ cells
***
0
5
10
15
Non-mutant
Mutant

Figure S2

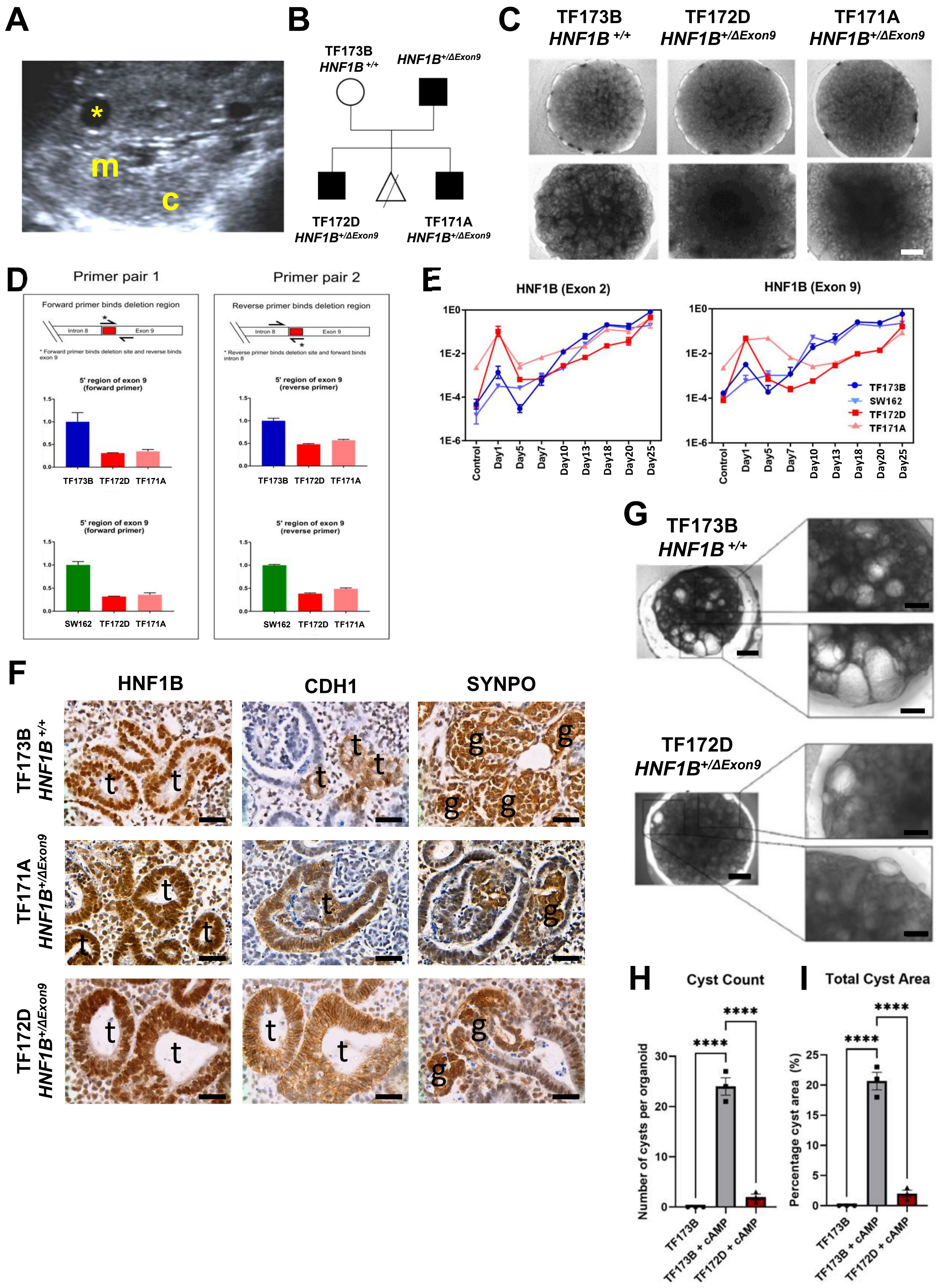

A
B
TF173B
HNF1B +/+
HNF1B+/ΔExon9
TF172D
HNF1B+/ΔExon9
TF171A
HNF1B+/ΔExon9
C
TF173B
HNF1B +/+
TF172D
HNF1B+/ΔExon9
TF171A
HNF1B+/ΔExon9
D
Primer pair 1
Primer pair 2
Forward primer binds deletion region
Reverse primer binds deletion region
5' region of exon 9 (forward primer)
5' region of exon 9 (reverse primer)
TF173B TF172D TF171A
SW162 TF172D TF171A
E
HNF1B (Exon 2)
HNF1B (Exon 9)
Control Day1 Day5 Day7 Day10 Day13 Day18 Day20 Day25
TF173B
SW162
TF172D
TF171A
F
HNF1B
CDH1
SYNPO
TF173B HNF1B +/+
TF171A HNF1B+/ΔExon9
TF172D HNF1B+/ΔExon9
G
TF173B
HNF1B +/+
TF172D
HNF1B+/ΔExon9
H
Cyst Count
Number of cysts per organoid
I
Total Cyst Area
Percentage cyst area (%)
TF173B
TF173B + cAMP
TF172D + cAMP

Figure S3

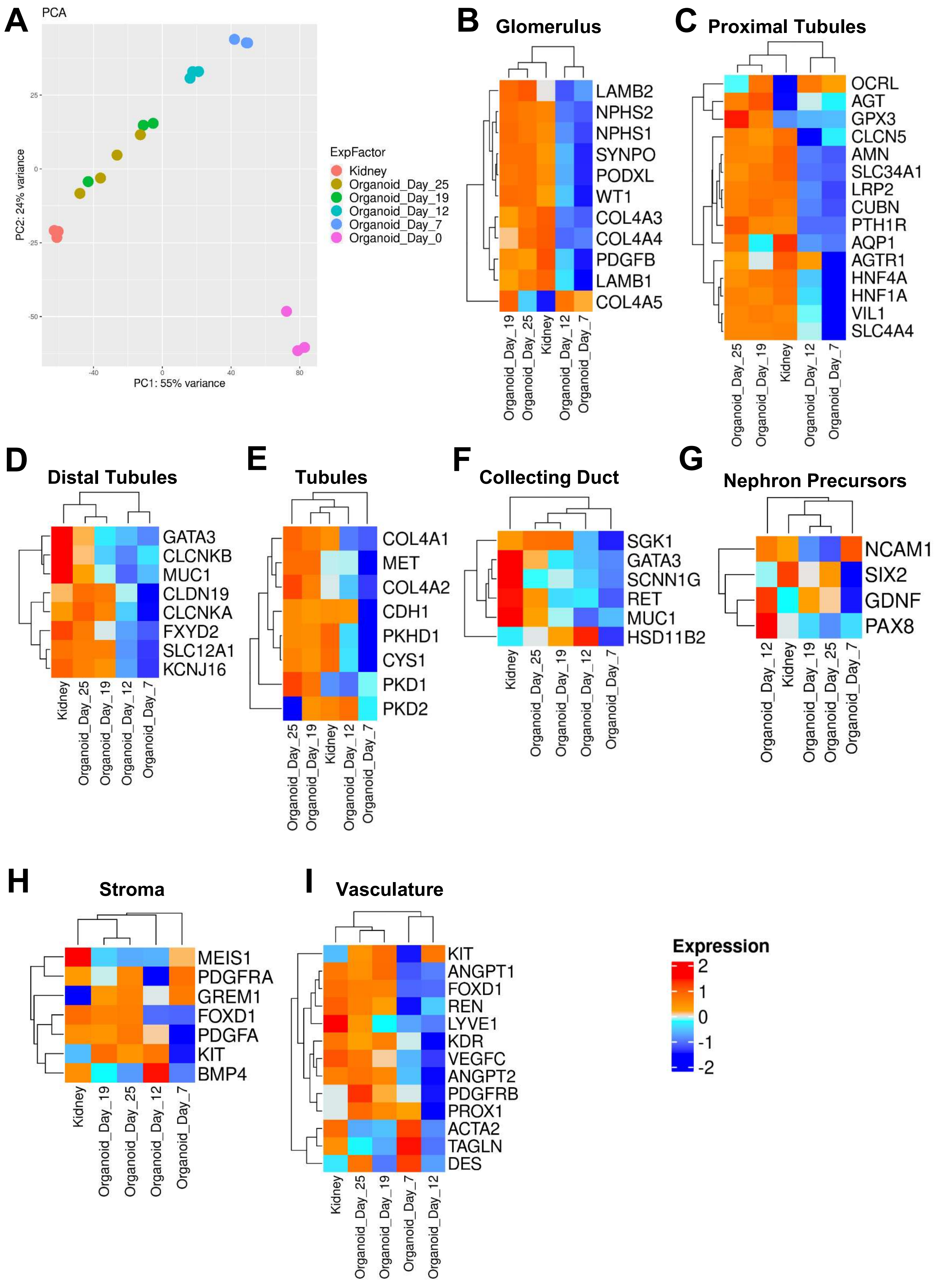
A
PCA
PC2: 24% variance
PC1: 55% variance
ExpFactor
Kidney
Organoid_Day_25
Organoid_Day_19
Organoid_Day_12
Organoid_Day_7
Organoid_Day_0
B
Glomerulus
LAMB2
NPHS2
NPHS1
SYNPO
PODXL
WT1
COL4A3
COL4A4
PDGFB
LAMB1
COL4A5
C
Proximal Tubules
OCRL
AGT
GPX3
CLCN5
AMN
SLC34A1
LRP2
CUBN
PTH1R
AQP1
AGTR1
HNF4A
HNF1A
VIL1
SLC4A4
D
Distal Tubules
GATA3
CLCNKB
MUC1
CLDN19
CLCNKA
FXYD2
SLC12A1
KCNJ16
E
Tubules
COL4A1
MET
COL4A2
CDH1
PKHD1
CYS1
PKD1
PKD2
F
Collecting Duct
SGK1
GATA3
SCNN1G
RET
MUC1
HSD11B2
G
Nephron Precursors
NCAM1
SIX2
GDNF
PAX8
H
Stroma
MEIS1
PDGFRA
GREM1
FOXD1
PDGFA
KIT
BMP4
I
Vasculature
KIT
ANGPT1
FOXD1
REN
LYVE1
KDR
VEGFC
ANGPT2
PDGFRB
PROX1
ACTA2
TAGLN
DES
Expression

Figure S3 (Continued)

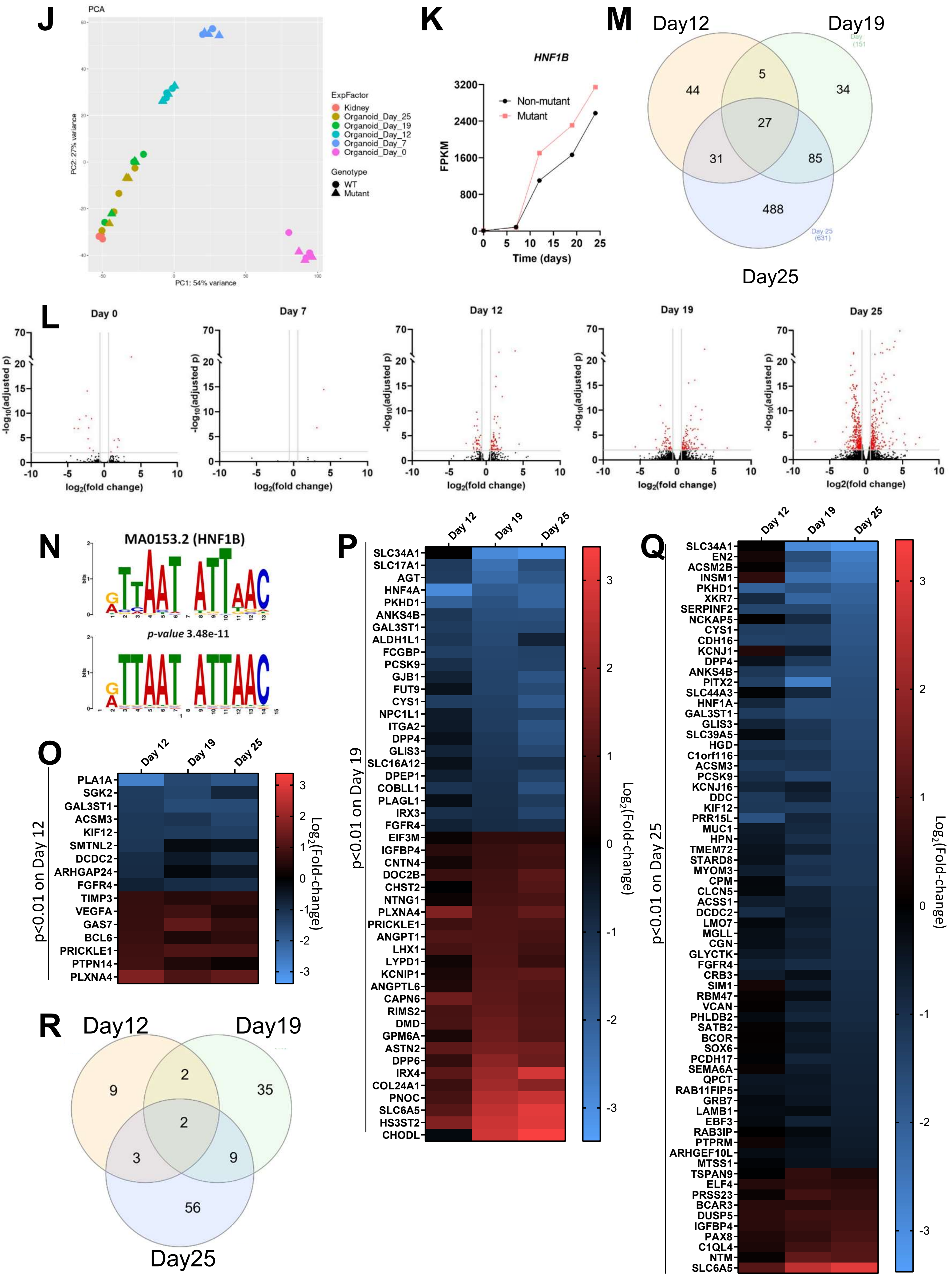

Figure S4

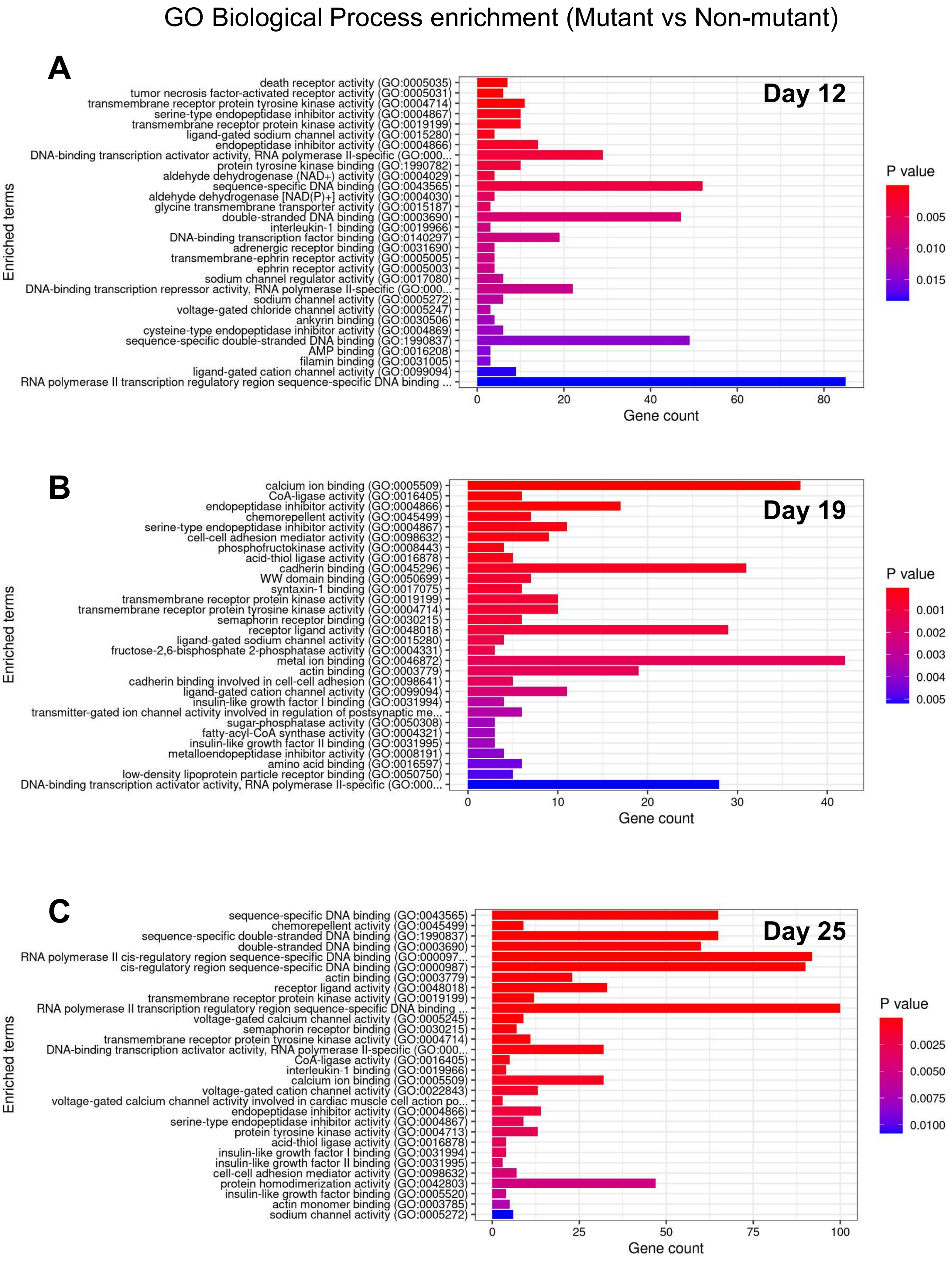
GO Biological Process enrichment (Mutant vs Non-mutant)
A
Day 12
death receptor activity (GO:0005035)
tumor necrosis factor-activated receptor activity (GO:0005031)
transmembrane receptor protein tyrosine kinase activity (GO:0004714)
serine-type endopeptidase inhibitor activity (GO:0004867)
transmembrane receptor protein kinase activity (GO:0019199)
ligand-gated sodium channel activity (GO:0015280)
endopeptidase inhibitor activity (GO:0004866)
DNA-binding transcription activator activity, RNA polymerase II-specific (GO:000...
protein tyrosine kinase binding (GO:1990782)
aldehyde dehydrogenase (NAD+) activity (GO:0004029)
sequence-specific DNA binding (GO:0043565)
aldehyde dehydrogenase [NAD(P)+] activity (GO:0004030)
glycine transmembrane transporter activity (GO:0015187)
double-stranded DNA binding (GO:0003690)
interleukin-1 binding (GO:0019966)
DNA-binding transcription factor binding (GO:0140297)
adrenergic receptor binding (GO:0031690)
transmembrane-ephrin receptor activity (GO:0005005)
ephrin receptor activity (GO:0005003)
sodium channel regulator activity (GO:0017080)
DNA-binding transcription repressor activity, RNA polymerase II-specific (GO:000...
sodium channel activity (GO:0005272)
voltage-gated chloride channel activity (GO:0005247)
ankyrin binding (GO:0030506)
cysteine-type endopeptidase inhibitor activity (GO:0004869)
sequence-specific double-stranded DNA binding (GO:1990837)
AMP binding (GO:0016208)
filamin binding (GO:0031005)
ligand-gated cation channel activity (GO:0099094)
RNA polymerase II transcription regulatory region sequence-specific DNA binding ...
Enriched terms
Gene count
0
20
40
60
80
P value
0.005
0.010
0.015
B
Day 19
calcium ion binding (GO:0005509)
CoA-ligase activity (GO:0016405)
endopeptidase inhibitor activity (GO:0004866)
chemorepellent activity (GO:0045499)
serine-type endopeptidase inhibitor activity (GO:0004867)
cell-cell adhesion mediator activity (GO:0098632)
phosphofructokinase activity (GO:0008443)
acid-thiol ligase activity (GO:0016878)
cadherin binding (GO:0045296)
WW domain binding (GO:0050699)
syntaxin-1 binding (GO:0017075)
transmembrane receptor protein kinase activity (GO:0019199)
transmembrane receptor protein tyrosine kinase activity (GO:0004714)
semaphorin receptor binding (GO:0030215)
receptor ligand activity (GO:0048018)
ligand-gated sodium channel activity (GO:0015280)
fructose-2,6-bisphosphate 2-phosphatase activity (GO:0004331)
metal ion binding (GO:0046872)
actin binding (GO:0003779)
cadherin binding involved in cell-cell adhesion (GO:0098641)
ligand-gated cation channel activity (GO:0099094)
insulin-like growth factor I binding (GO:0031994)
transmitter-gated ion channel activity involved in regulation of postsynaptic me...
sugar-phosphatase activity (GO:0050308)
fatty-acyl-CoA synthase activity (GO:0004321)
insulin-like growth factor II binding (GO:0031995)
metalloendopeptidase inhibitor activity (GO:0008191)
amino acid binding (GO:0016597)
low-density lipoprotein particle receptor binding (GO:0050750)
DNA-binding transcription activator activity, RNA polymerase II-specific (GO:000...
Enriched terms
Gene count
0
10
20
30
40
P value
0.001
0.002
0.003
0.004
0.005
C
Day 25
sequence-specific DNA binding (GO:0043565)
chemorepellent activity (GO:0045499)
sequence-specific double-stranded DNA binding (GO:1990837)
double-stranded DNA binding (GO:0003690)
RNA polymerase II cis-regulatory region sequence-specific DNA binding (GO:000097...
cis-regulatory region sequence-specific DNA binding (GO:0000987)
actin binding (GO:0003779)
receptor ligand activity (GO:0048018)
transmembrane receptor protein kinase activity (GO:0019199)
RNA polymerase II transcription regulatory region sequence-specific DNA binding ...
voltage-gated calcium channel activity (GO:0005245)
semaphorin receptor binding (GO:0030215)
transmembrane receptor protein tyrosine kinase activity (GO:0004714)
DNA-binding transcription activator activity, RNA polymerase II-specific (GO:000...
CoA-ligase activity (GO:0016405)
interleukin-1 binding (GO:0019966)
calcium ion binding (GO:0005509)
voltage-gated cation channel activity (GO:0022843)
voltage-gated calcium channel activity involved in cardiac muscle cell action po...
endopeptidase inhibitor activity (GO:0004866)
serine-type endopeptidase inhibitor activity (GO:0004867)
protein tyrosine kinase activity (GO:0004713)
acid-thiol ligase activity (GO:0016878)
insulin-like growth factor I binding (GO:0031994)
insulin-like growth factor II binding (GO:0031995)
cell-cell adhesion mediator activity (GO:0098632)
protein homodimerization activity (GO:0042803)
insulin-like growth factor binding (GO:0005520)
actin monomer binding (GO:0003785)
sodium channel activity (GO:0005272)
Enriched terms
Gene count
0
25
50
75
100
P value
0.0025
0.0050
0.0075
0.0100

Figure S4 (continued)

GO Molecular Function enrichment (Mutant vs Non-mutant)

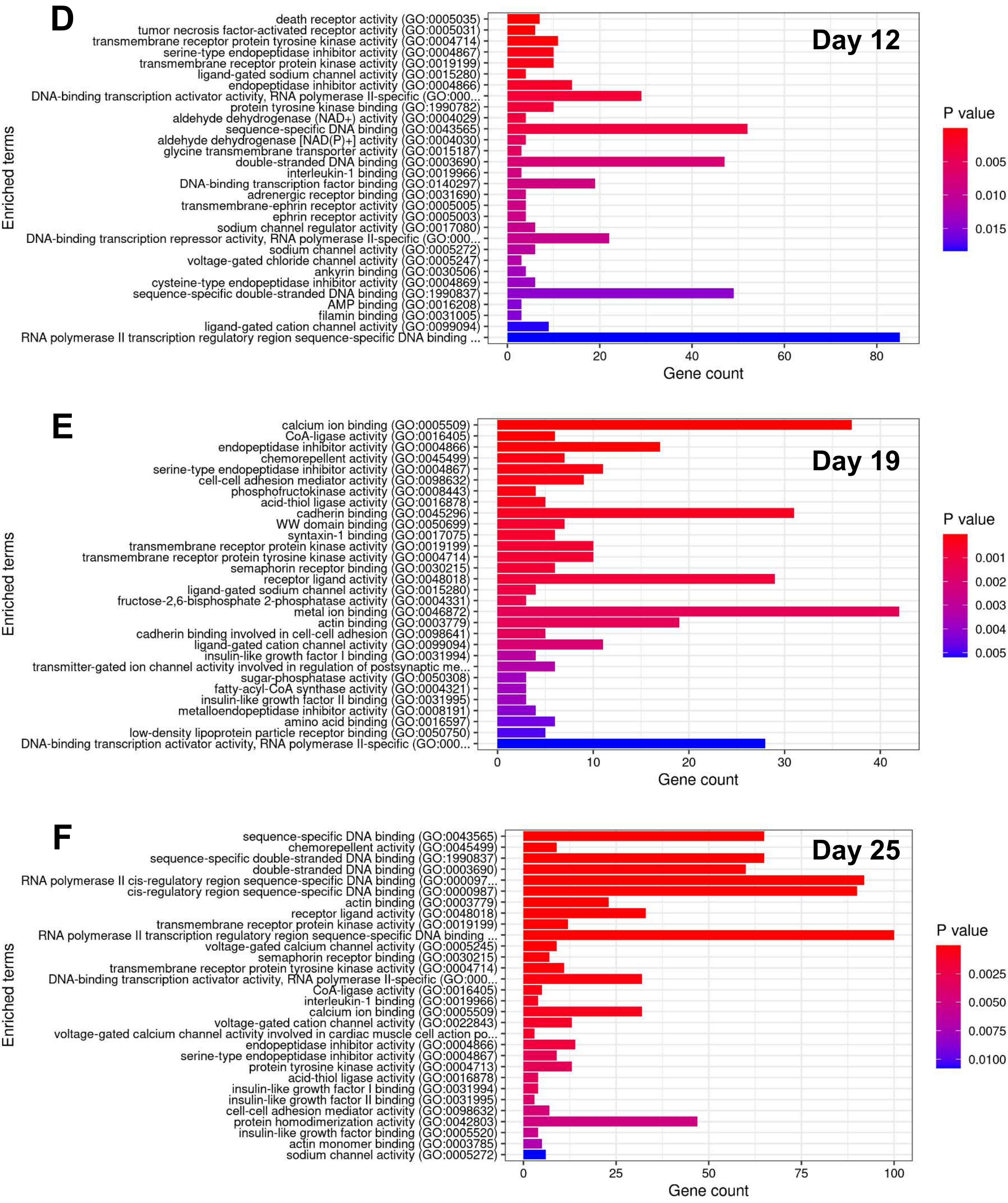

Figure S4 (continued)

GO Cellular Compartment enrichment (Mutant vs Non-mutant)

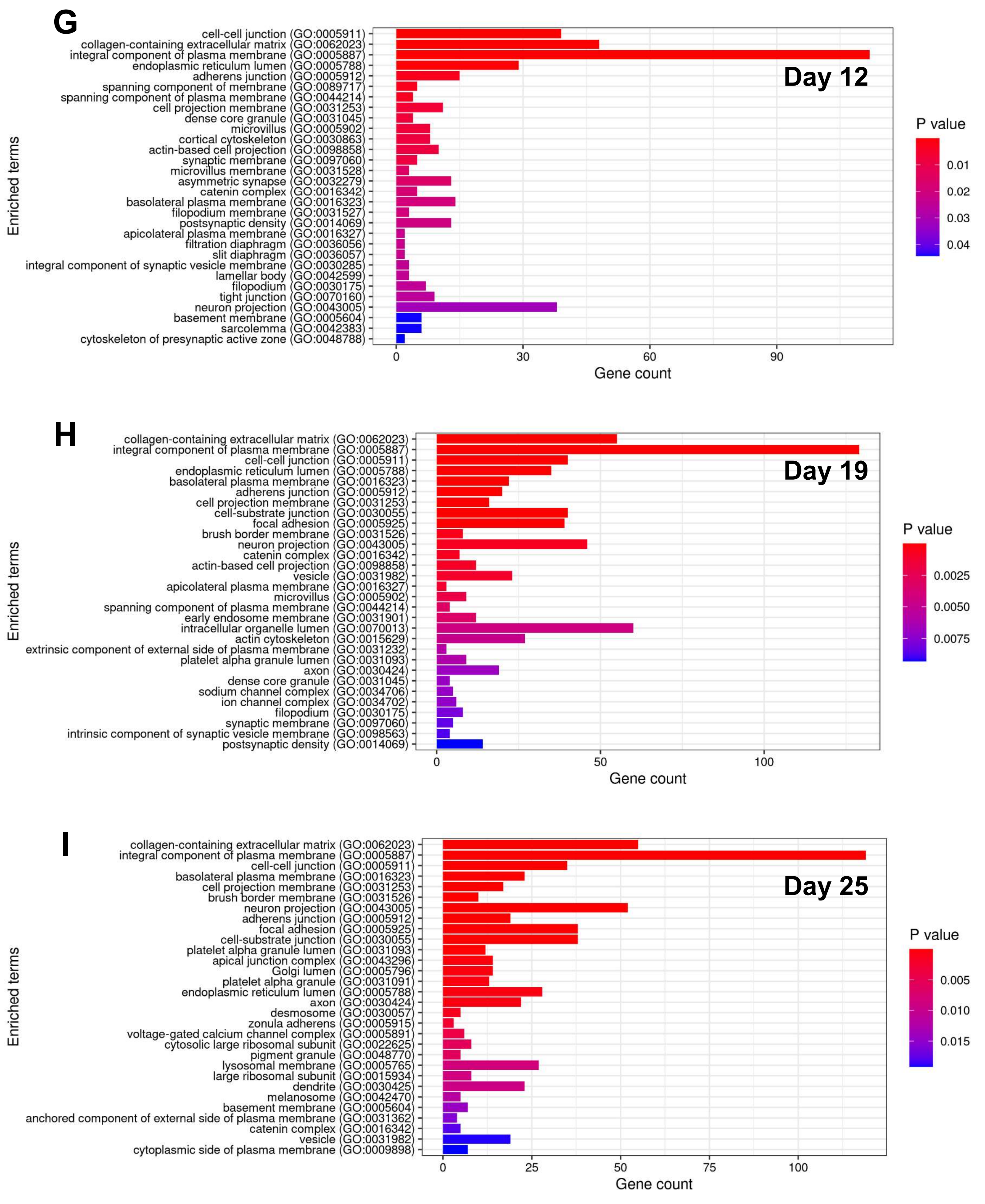

Figure S5

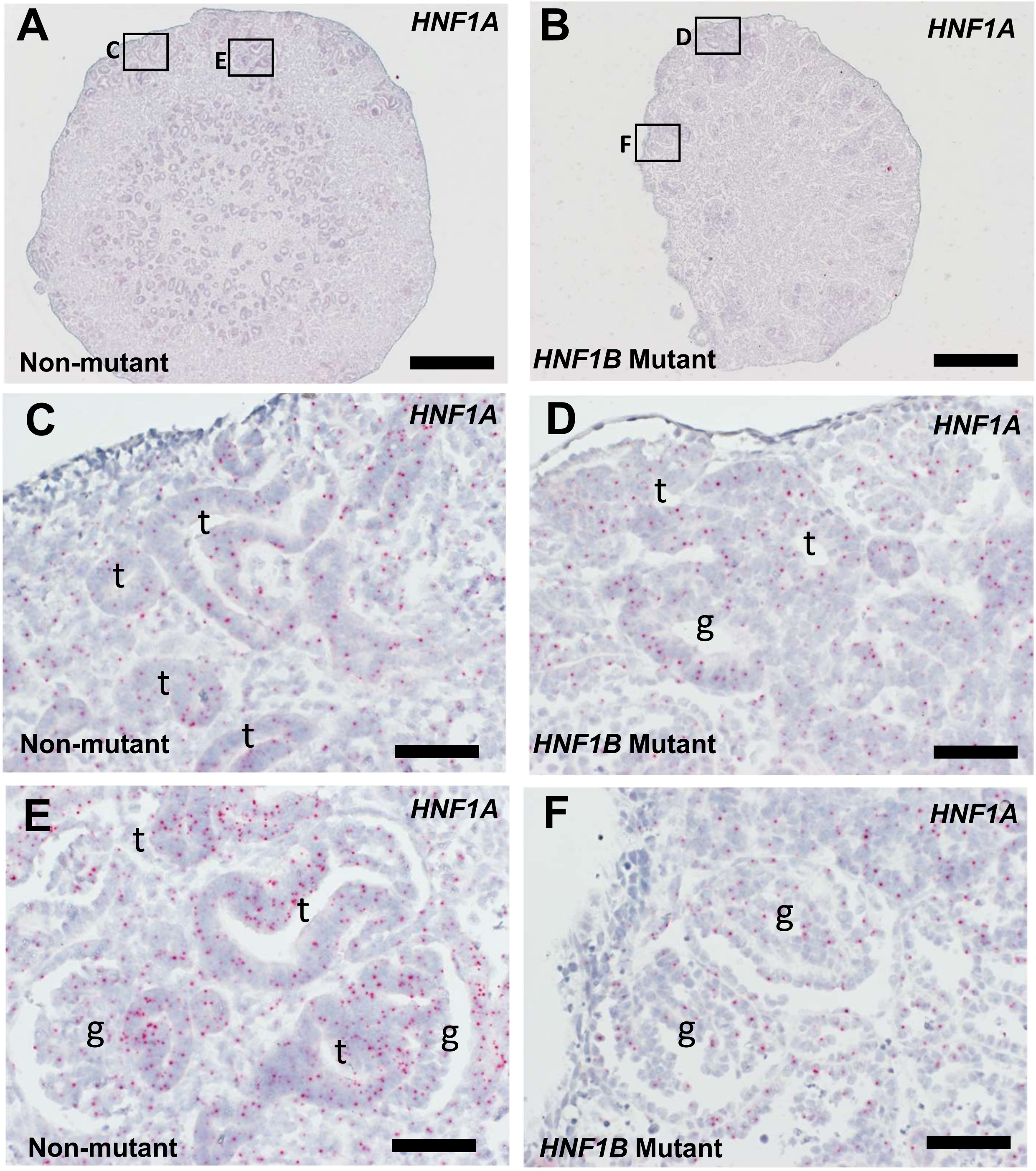
A
HNF1A
C
E
Non-mutant
B
HNF1A
D
F
HNF1B Mutant
C
HNF1A
t
t
t
t
Non-mutant
D
HNF1A
t
t
g
HNF1B Mutant
E
HNF1A
t
t
g
t
g
Non-mutant
F
HNF1A
g
g
HNF1B Mutant

Figure S6

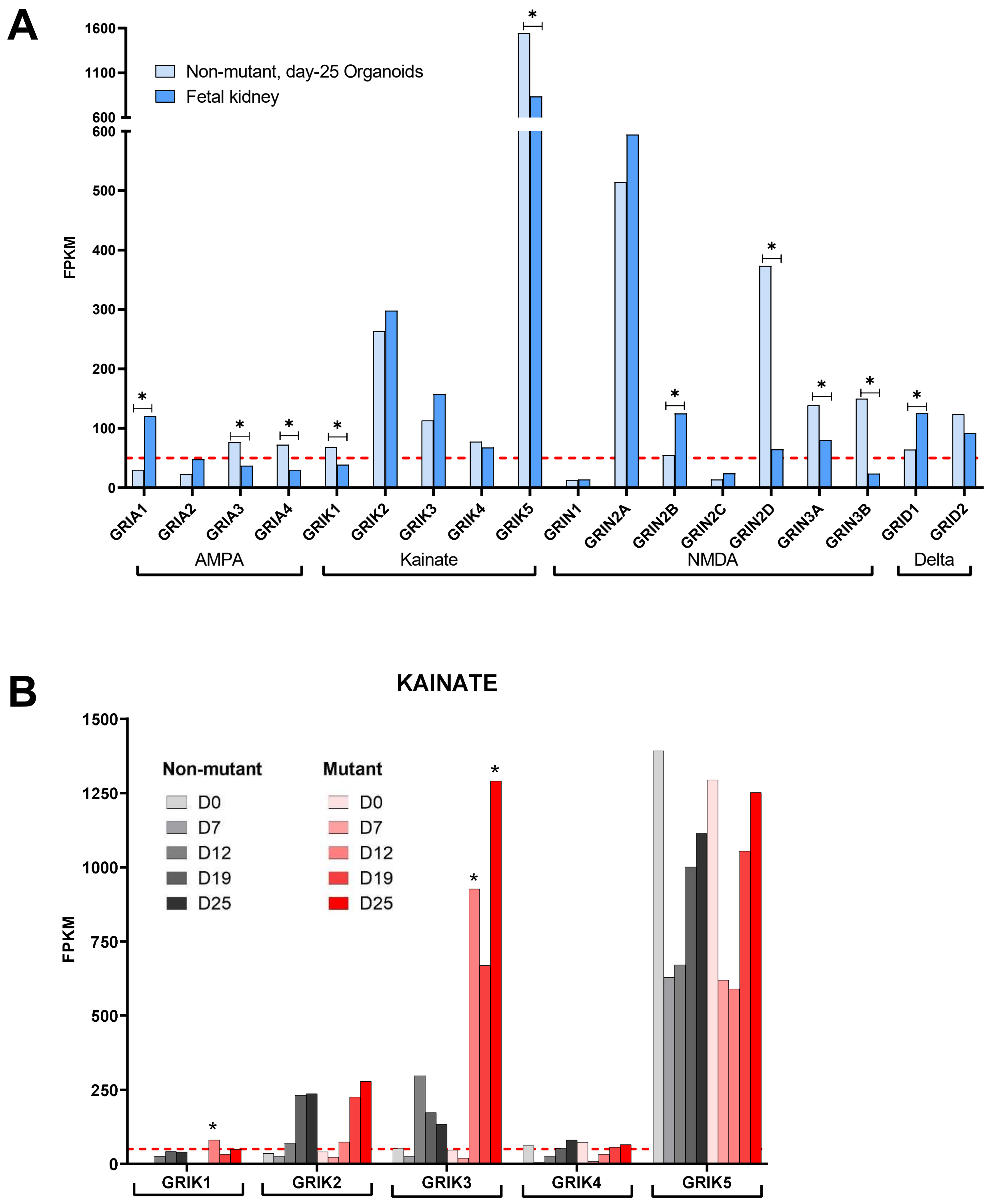
A
1600
1100
600
600
500
400
300
200
100
0
FPKM
Non-mutant, day-25 Organoids
Fetal kidney
GRIA1
GRIA2
GRIA3
GRIA4
GRIK1
GRIK2
GRIK3
GRIK4
GRIK5
GRIN1
GRIN2A
GRIN2B
GRIN2C
GRIN2D
GRIN3A
GRIN3B
GRID1
GRID2
AMPA
Kainate
NMDA
Delta
B
KAINATE
1500
1250
1000
750
500
250
0
FPKM
Non-mutant
D0
D7
D12
D19
D25
Mutant
D0
D7
D12
D19
D25
GRIK1
GRIK2
GRIK3
GRIK4
GRIK5

Figure S6 (Continued)

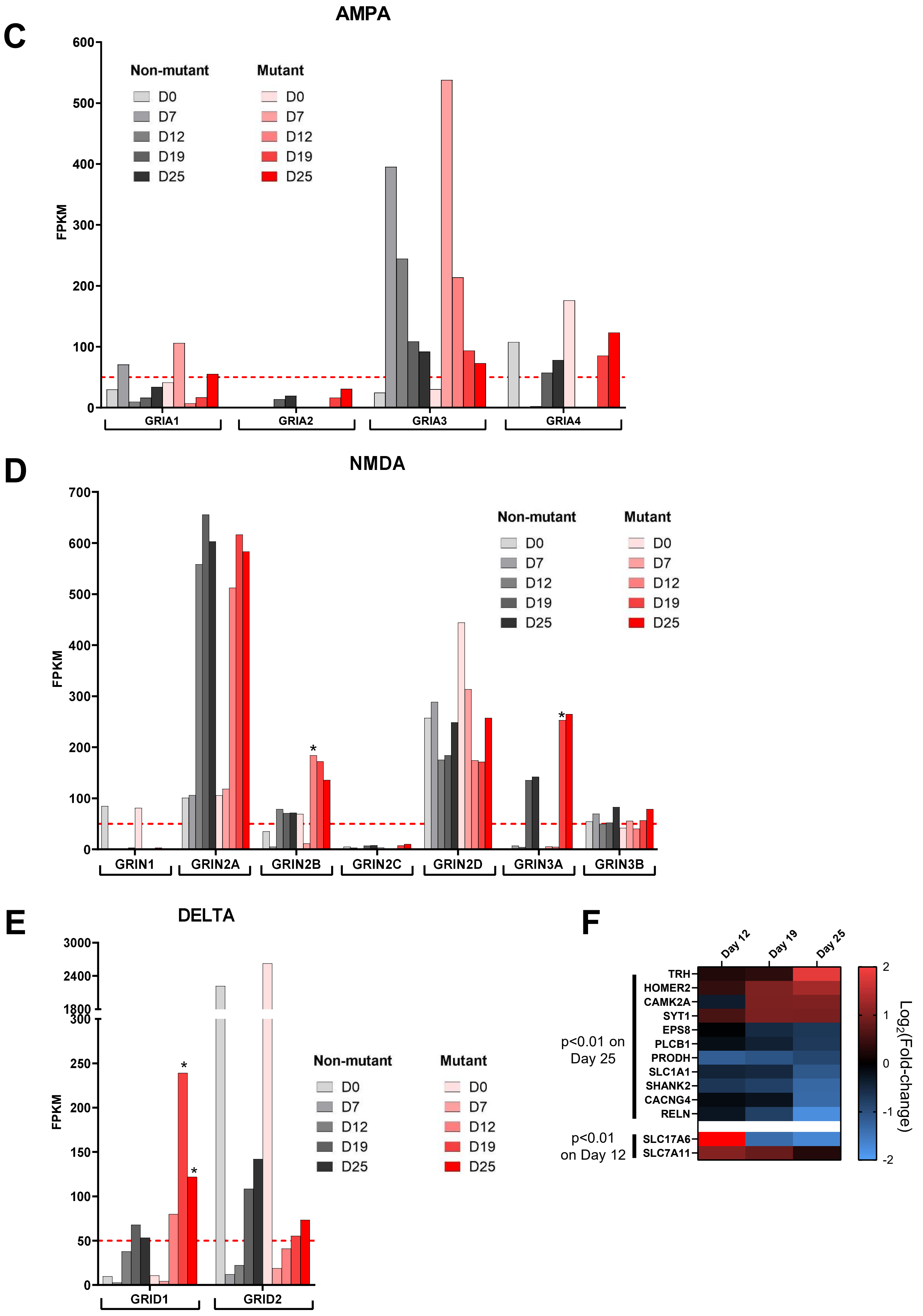

Figure S6 (Continued)

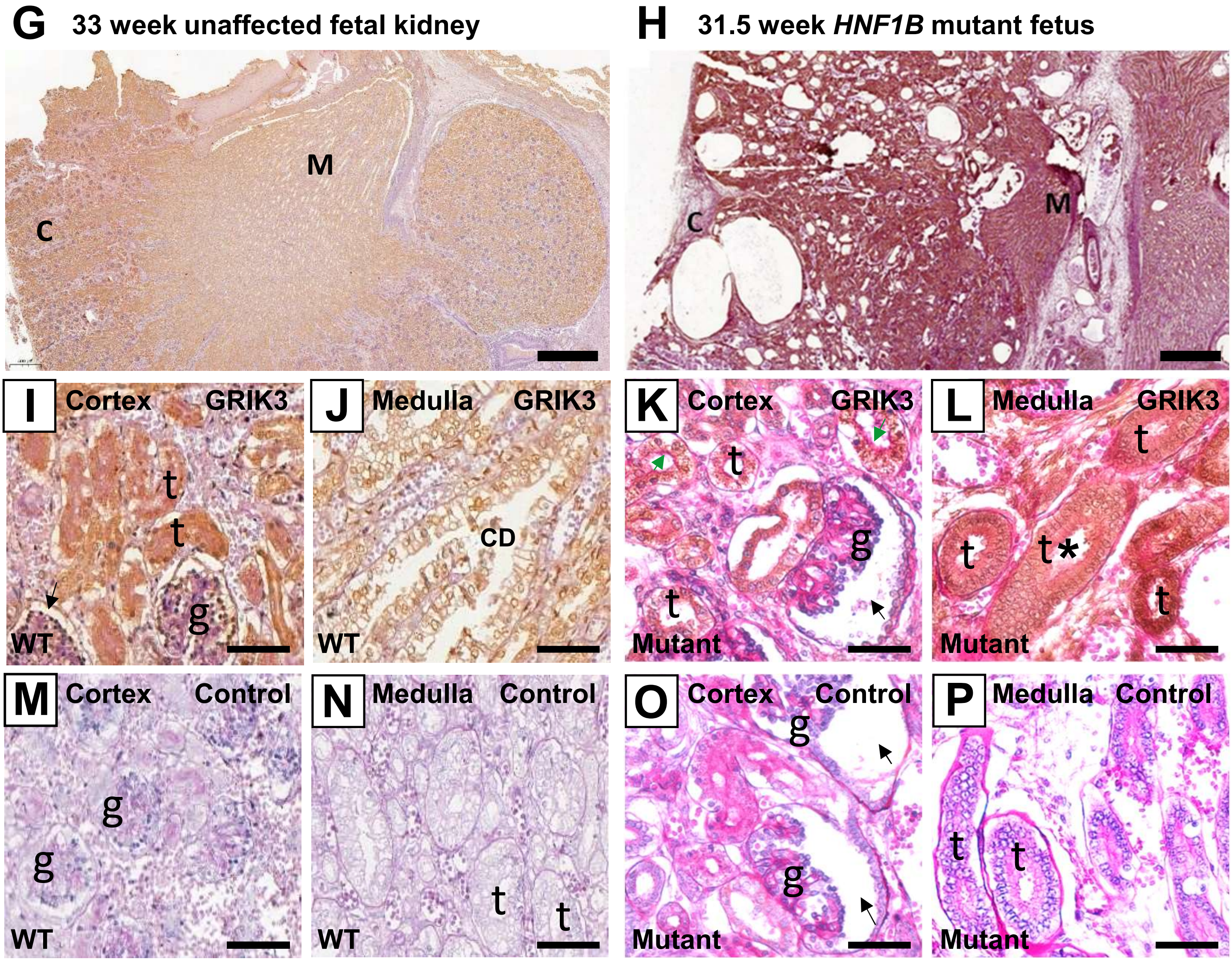

Figure S7

A

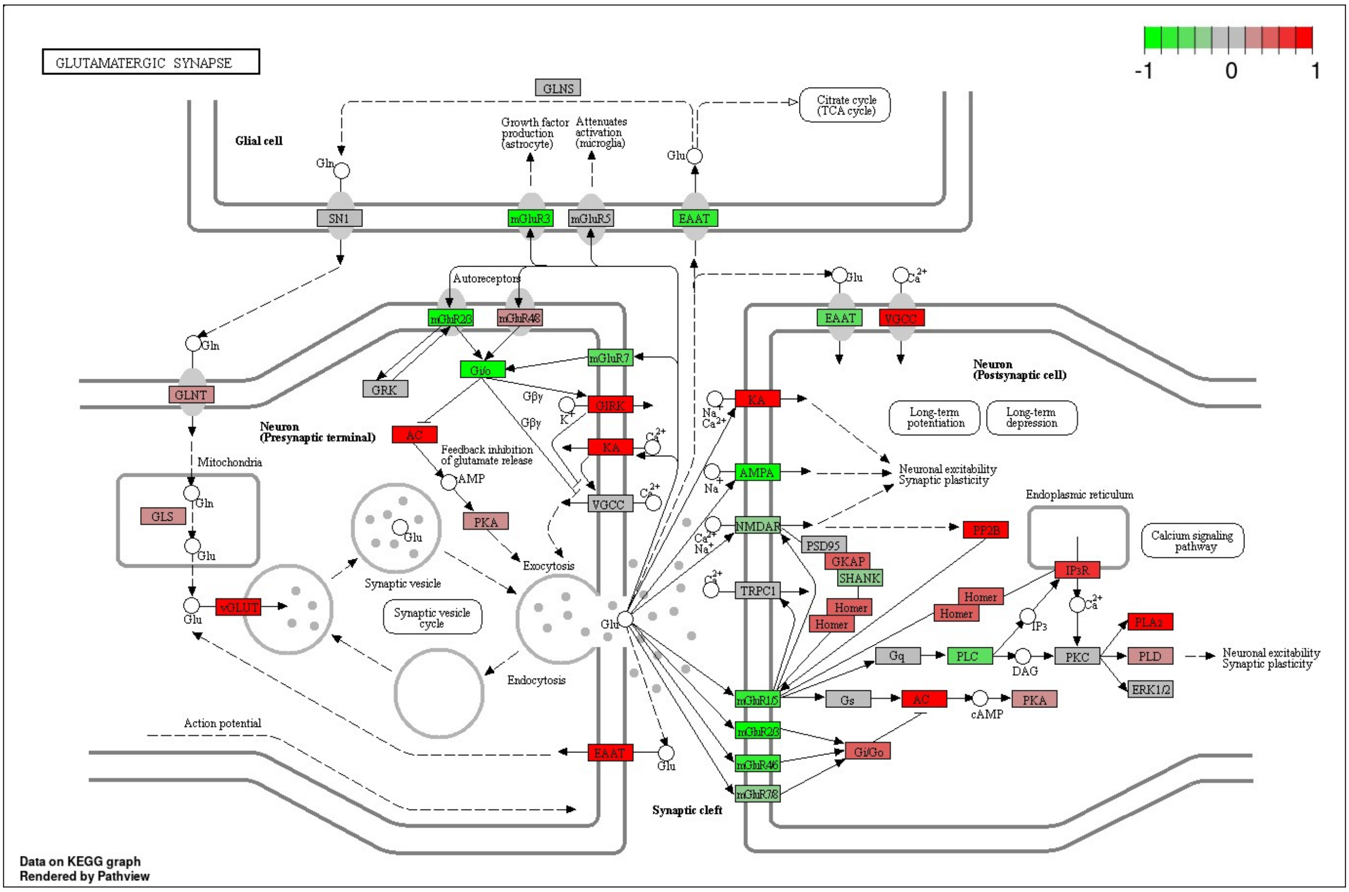
GLUTAMATERGIC SYNAPSE
-1
0
1
Glial cell
Neuron (Presynaptic terminal)
Neuron (Postsynaptic cell)
Synaptic cleft
Mitochondria
Synaptic vesicle
Synaptic vesicle cycle
Exocytosis
Endocytosis
Action potential
Autoreceptors
Feedback inhibition of glutamate release
Growth factor production (astrocyte)
Attenuates activation (microglia)
Citrate cycle (TCA cycle)
Long-term potentiation
Long-term depression
Neuronal excitability Synaptic plasticity
Endoplasmic reticulum
Calcium signaling pathway
GLNS
SN1
mGluR3
mGluR5
EAAT
GLNT
GLS
vGLUT
mGluR2/3
mGluR4/8
mGluR7
GRK
Gi/o
GIRK
AC
KA
PKA
VGCC
AMPA
NMDAR
TRPC1
PSD95
GKAP
SHANK
Homer
PP2B
IP3R
PLA2
PLD
PKC
ERK1/2
Gq
PLC
DAG
IP3
mGluR1/5
mGluR2/3
mGluR4/6
mGluR7/8
Gs
cAMP
Gi/Go
Data on KEGG graph
Rendered by Pathview

B

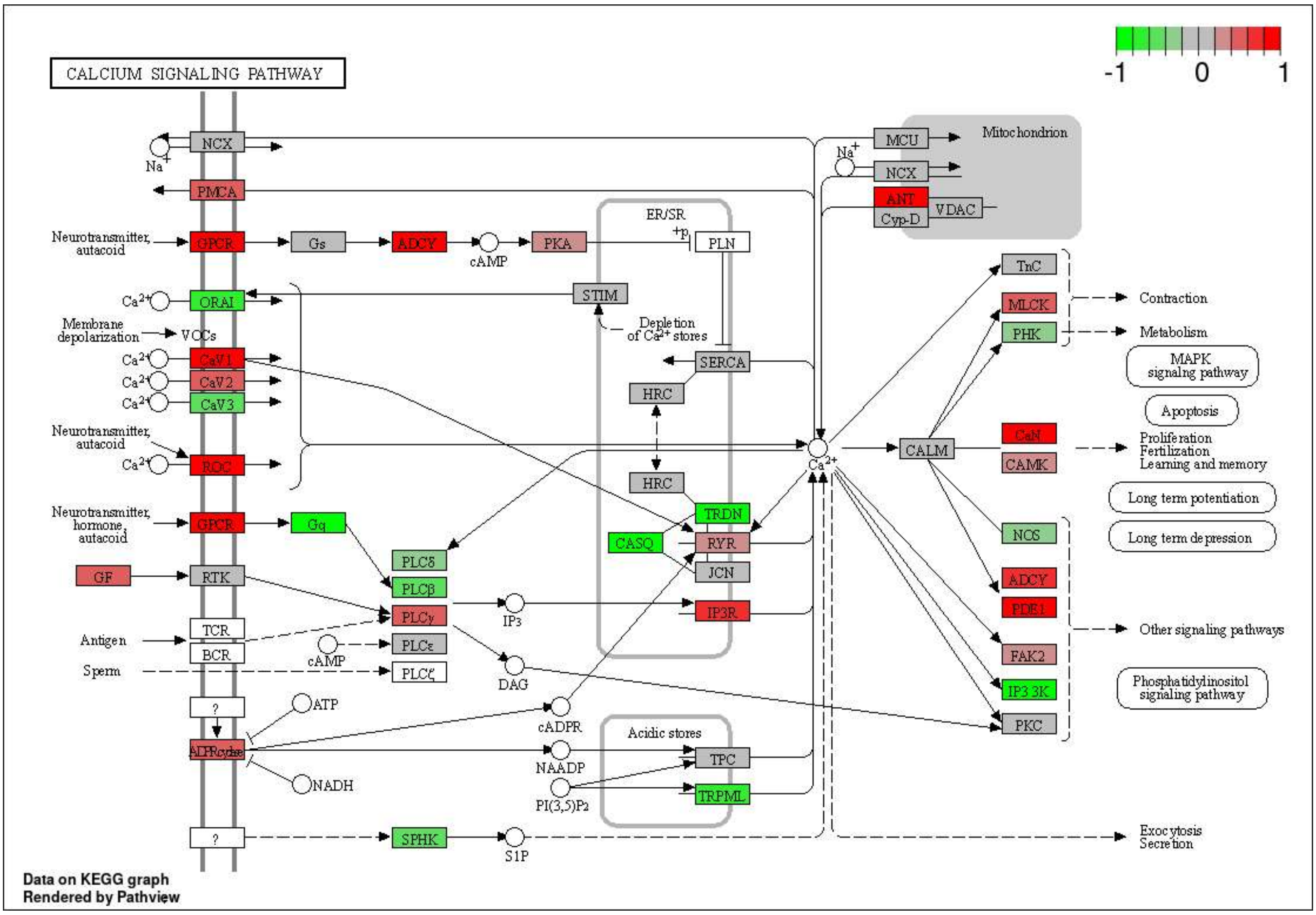
CALCIUM SIGNALING PATHWAY
-1
0
1
Mitochondrion
ER/SR
Acidic stores
NCX
PMCA
GPCR
Gs
ADCY
cAMP
PKA
PLN
ORAI
STIM
VOCs
CaV1
CaV2
CaV3
ROC
Gq
RTK
GF
TCR
BCR
PLCδ
PLCβ
PLCγ
PLCε
PLCζ
SERCA
HRC
TRDN
CASQ
RYR
JCN
IP3R
IP3
DAG
cADPR
NAADP
PI(3,5)P2
ADPRcyclase
TPC
TRPML
SPHK
S1P
MCU
ANT
Cyp-D
VDAC
CALM
TnC
MLCK
PHK
CaN
CAMK
NOS
PDE1
FAK2
IP3 3K
PKC
Neurotransmitter, autacoid
Membrane depolarization
Neurotransmitter, hormone, autacoid
Antigen
Sperm
Depletion of Ca2+ stores
Contraction
Metabolism
MAPK signaling pathway
Apoptosis
Proliferation Fertilization Learning and memory
Long term potentiation
Long term depression
Other signaling pathways
Phosphatidylinositol signaling pathway
Exocytosis Secretion
Data on KEGG graph
Rendered by Pathview

SUPPLEMENTAL FIGURE LEGENDS

Figure S1 (Related to Figure 1 and 2). Generation of heterozygous *HNF1B* mutant hESCs and their differentiation towards kidney tissues. (A) Sequence in exon 1 of *HNF1B*. Red dotted line and scissors mark the editing site, with gRNA binding sequences in red. **(B)** Sequencing chromatograms of exon 1 confirming the wild-type sequence in IBM13-08 (*non-mutant*) hESCs and the heterozygous frameshift starting (*red arrow*) at the CRISPR-targeted site in IBM13-19 (*mutant*) hESCs. **(C)** 2D kidney differentiation cultures (day 12) confirmed both lines formed CDH1+ aggregates but HNF1B immunoreactivity appeared less in mutant cells. **(D)** 25-day organoid protocol. **(E)** Phase contrast images of organoids. **(F)** Day 25 wild-type and mutant organoids had similar areas. **(G)** Mutant organoids contained bulkier internal structures than wild-type organoids. Synaptopodin (SYNPO) immunostaining (brown) of glomeruli in non-mutant (**H**) and *HNF1B* heterozygous mutant (**I**) organoids with haematoxylin counterstain (blue). Mutant glomeruli contained less compact podocyte tufts. PECAM1 immunostaining (brown) in non-mutant (**J**) and mutant (**K**) organoids with haematoxylin counterstain (blue) showed capillary-like structures between tubules, while glomerular tufts (examples denoted by red arrows) were largely PECAM1-negative. BrdU immunostaining (brown) marking proliferative cells in non-mutant (**L**) and mutant (**M**) organoids with haematoxylin counterstain (blue). Tubules indicated by *t*. **(N)** Quantification showed a significantly increased percentage of BrdU+ nuclei in mutant compared with non-mutant tubules (mean±SEM; n=8 non-mutant and n=7 mutant organoids across three independent differentiation experiments; ****p<0.00005, t-test). Activated caspase 3 immunostaining (brown) marking cells undergoing apoptosis in non-mutant (**O**) and mutant (**P**) organoids with haematoxylin counterstain (blue). **(Q)** Significantly increased percentage of activated caspase 3 immunostained cells in mutant compared with non-mutant organoids (mean±SEM; n=9 non-mutant and n=7 mutant organoids across three independent differentiation experiments; ***p<0.0005, t-test). Bars: (C) and (G) 100 µM; (E) 1 mm; (H-M and O-P) 50 µM.

Figure S2 (Related to Figures 2 and 3). Derivation of patient-derived iPSC lines and characterisation of the resultant organoids. (**A**) Kidney ultrasonography of affected *HNF1B*$^{+/\Delta Exon9}$ male (TF172). Note lack of distinction between the echogenic cortex (c) and medulla (m); asterisk indicates a dilated structure. **(B)** Kindred from which iPSCs were derived. Black icons indicate individuals with DKMs. The triangle indicates the affected fetus who underwent elective termination. **(C)** Phase contrast images of organoids at day 15 (top images) and day 18 (bottom images). **(D)** qPCR of genomic DNA from *HNF1B*$^{+/\Delta Exon9}$ iPSCs (TF171A and TF172D) and unaffected control *HNF1B*$^{+/+}$ iPSCs (TF173B and SW160) with two primer pairs binding to the predicted deletion site in exon 9. A halving of gene dosage in TF171A and TF172D was detected compared with the *HNF1B*$^{+/+}$ mother (TF173B) and an unrelated control (SW160), confirming the heterozygous nature of the mutation (mean±SEM from 3 technical replicates). **(E)** qPCR time course of *HNF1B* mRNA levels, detected using either exon 2 (top) or exon 9-specific (bottom) primers. The former would detect *HNF1B* mRNA generated by wild-type and mutant alleles, whereas the latter detects only wild-type mRNA. Two *HNF1B*$^{+/+}$ iPSC lines were used for comparison, TF173B (mother) and SW160 (an unrelated control). Transcript levels detected by exon 9 primers were lower in mutants than in controls during the organoid phase (*day 7+3* onwards). **(F)** Immunohistochemical comparison of *HNF1B*$^{+/\Delta Exon9}$ with *HNF1B*$^{+/+}$ control organoids. Note aberrant multi-layered CDH1+ and HNF1B+ tubules (t) and dysmorphic SYNPO+ glomeruli (g) in mutant tissues. Positive immunostaining (brown) with haematoxylin counterstain (blue). **(G)** 8-Br-cAMP-induced dilated structures appeared fewer in *HNF1B*$^{+/\Delta Exon9}$ organoids. **(H)** Quantification of numbers of dilated structures per organoid in *HNF1B*$^{+/+}$ (TF173B) and *HNF1B*$^{+/\Delta Exon9}$ (TF172D) organoids following 8-Br-cAMP exposure showing markedly lower numbers of dilated structures in *HNF1B*$^{+/\Delta Exon9}$ organoids (n=3 organoids across three differentiation experiments; p****<0.00005, one-way ANOVA with multiple comparisons). **(I)** Quantification of percentage dilatation area per organoid in *HNF1B*$^{+/+}$ (TF173B) and *HNF1B*$^{+/\Delta Exon9}$ (TF172D) organoids following 8-Br-cAMP exposure. Note significantly decreased percentage area of dilated structures in *HNF1B*$^{+/\Delta Exon9}$ organoids (n=3 organoids across 3 independent differentiation experiments: p****<0.00005, one-way ANOVA with multiple comparisons). Bars: (C) 500 µM; (F) 20 µM; (G) 500 µm (left frames) and 20 µm (right frames).

Figure S3 (Related to Figure 4). Transcriptomic profiling of differentiating non-mutant kidney organoids in comparison to fetal kidney (A-I), and transcriptional profiles of heterozygous *HNF1B* mutant cells compared with isogenic wild-type controls during differentiation to organoids (J-R). (A) PCA of RNAseq of differentiating wild-type hESCs. As differentiation proceeded

from PSCs (*Organoid day 0*), profiles approached those of 8-10 week human fetal kidneys. Profiles of day 19 and 25 organoids were similar, suggesting that this period represents a plateau of *in vitro* differentiation when the whole transcriptome is considered. **(C-G)** Heat maps of gene expression marking specific cellular kidney compartments. Glomerular and PT transcripts rose during organoid maturation so that by days 19 and 25 they resembled those in fetal kidneys. These genes include *CUBN* and *LRP2*, whose protein products Cubilin and Megalin, were studied in organoids in Fig. 3. While some DT and CD genes were expressed, they tended to remain lower than in the fetal kidney. The *Tubule* panel showed several kidney epithelial genes whose expression was not limited to particular nephron segments or CDs. Several *Nephron precursor* genes are expressed in organoids and tended to be downregulated by day 25. Organoids also expressed several genes characteristic of blood and lymphatic vessels (*Vasculature*) and stromal cells. Each point of the heat map represents the mean of three (days 7-19) or four ('Kidney' and day-25) independent differentiation experiments. **(J)** PCA showing that progression of differentiation over the 25 day protocol was similar between non-mutant and *HNF1B*-mutant organoids**. (K)** *HNF1B* transcripts in the bulk RNAseq increased similarly in each genotype. **(L)** Volcano plots demonstrating the progressive increase in numbers of significantly (red) differentially expressed genes during the organoid phase (*Day 12* onwards). **(M)** Venn diagram of significantly differentially expressed transcripts in the organoid phase of culture. **(N)** Top: Sequence motif logo of HNF1B (MA0153.2; p-value 3.48×10^{-11}) transcription factor, within significant differentially expressed genes (padj≤0.05), created by TOMTOM from JASPAR2018_CORE_vertebrates_non-redundant database. Bottom: canonical *HNF1B* binding site used in this analysis. **(O-Q)** Heatmaps showing the difference between non-mutant and *HNF1B* mutant organoids, in expression levels of genes that contain the *HNF1B*-binding consensus sequence in their promoters, on day (O) 12, (P) 19 and (Q) 25 of differentiation. Note that on days 12 and 19 an approximate equal number of genes were down- (blue) or up- (red) regulated, whereas on day 25 the majority of differentially expressed genes were downregulated. **(R)** Venn diagram quantifying the numbers of significantly differentially expressed genes at three stages of organoid culture.

Figure S4 (Related to Figure 4). GO terms enrichment analysis comparing isogenic non-mutant and *HNF1B* mutant organoids across organoid development. (A-I) Bar charts of terms enriched in mutant organoids compared with non-mutant on days 12, 19 and 25 of differentiation. Graphs depict *biological process* (A-C), *molecular function* (D-F) and *cellular compartment* (G-I). Red bars indicate upregulated in mutant organoids, and blue indicate downregulated in mutant organoids.

Figure S5 (Related to Figure 4). *HNF1A* transcript localisation in organoids. *HNF1A* Basescope ISH was performed on histological sections of kidney organoids at day 25. Signals appear as red dots; nuclei were counterstained blue with haematoxylin. **(A-B)** Low power overview of non-mutant and *HNF1B* mutant organoid. **(C-F)** High power frames of boxes indicated in (A-B). Note generally decreased *HNF1A* expression in mutant tubules (D) compared with non-mutant tubules (C, E). "t", tubules; "g", glomeruli. Scale bars: (A-B) 200 µM; (C-F) 20 µM.

Figure S6 (Related to Figure 5). Expression of glutamate receptors (GluR) in human fetal kidney and hESC-derived kidney organoids. (A) GluR subunit expression (average reads from bulk RNAseq) in non-mutant day 25 organoids (light blue) and human fetal kidneys (dark blue). In general, expression levels were of similar magnitude between organoids and native kidneys, with some differences indicated (n=4 independent differentiation experiments, *adjusted p<0.05). **(B-E)** Time course of expression of members of different families of GluR subunits in non-mutant (*grey/black)* and *HNF1B* mutant (*pink/red*) organoids. Differences between time-matched mutant and non-mutant organoids are indicated (n=3 independent differentiation experiments, *adjusted p<0.05). **(F)** Heatmap of genes belonging to the GO pathways term Glutamate Signalling (excluding GluR themselves), with significantly different (adjusted p<0.01) expression in *HNF1B* mutant organoids, in our RNAseq dataset. **(G-P)** GRIK3 immunostaining of human fetal kidneys: G, I, J, M and N are sections of a control third trimester kidney, and H, K, L, O and P are from a third trimester DKM from a fetus with a heterozygous frameshift in exon 7 of *HNF1B*. All panels were counterstained with haematoxylin (blue nuclei) and PAS (pink basement membrane, tubule brush border and interstitial scarring). G-L were also immunostained for GRIK3 (brown) and in M-P the primary antibody was omitted. **(G)** Low power overview of the control kidney; note the normal arrangement of the cortex ("C") and medulla ("M"). **(H)** Low power overview of

the *HNF1B* mutant kidney; note the disorganised arrangement of cortex (“C”) and medulla (“M”) and cysts. **(I)** GRIK3 immunostaining in cortical tubules (t) and Bowman capsule (arrowhead) in the control kidney. **(J)** GRIK3 immunostaining in a branched collecting duct (“CD”) in the control kidney. **(K)** Mutant kidney contained glomeruli with dilated Bowman spaces (black arrow) and GRIK3 immunostaining in large cortical tubules (t) that contained PAS+ brush border (green arrows), suggesting they are PT-like tubules. **(L)** Other areas of the mutant DKM were rich in multi-layered dysplastic tubules (t with asterisk in lumen) and that immunostained for GRIK3. **(M-P)** are similar views to I-L but with primary antibody omitted. Scale bars: (G-H) 500 μM (I-P) 40 μM.

Figure S7 (Related to Figure 5). KEGG pathway enrichment analysis for glutamatergic synapse and calcium signalling pathways. Red icons indicate upregulated and green icons indicate downregulated in mutant compared to control organoids with respect to (A) glutamatergic synapse and (B) calcium signalling.

SUPPLEMENTAL TABLES

Table S1. (Related to Experimental Procedures and Figure S2). Summary of iPSC clones isolated after reprogramming of patient PBMCs from a family with a heterozygous deletion of exon 9 of *HNF1B*.

Line design-ation	Mutation	Clinical presentation	Relationship		No. of Clones isolated
TF171	HNF1B Mutation: c.1654-?_1674+?del which causes a deletion of exon 9.	Dysplastic kidneys detected antenatally	Family of 3	son	6
TF172	HNF1B Mutation: c.1654-?_1674+?del which causes a deletion of exon 9.	Dysplastic kidneys detected antenatally		son	8
TF173	No mutation	Healthy		mother	8

Table S2. (Related to Experimental Procedures). Primers used in the study

**Primer Description**	**Application**	**Sequence**
DLG4 Forward	qPCR	GAACACGTATGATGTTGTCTACC
Reverse		TGCTGGGAATAAGAGGTTGTG
GAPDH Forward	qPCR	AGCCACATCGCTCAGACAC
Reverse		GCCCAATACGACCAAATCC
GRID1 Forward	qPCR	TGAATGAGGAAATCAGTGACCC
Reverse		CTCGTGCATTTCTGATTGTCC
GRID2 Forward	qPCR	CCTTCATTACTGAGGTTGTGGA
Reverse		GAACTGGAAATGTCTGCCGA
GRIK1 Forward	qPCR	ACAGCACAGGTCTAATTCGTC
Reverse		CATCTTTATTCCCAGAGGGCA
GRIK3 Forward	qPCR	CACCACTCTGGATCTCTACG
Reverse		GGTTGTCCACATTGAGAATCC
GRIK5 Forward	qPCR	GACCTTCTTCCAGAATTCACG
Reverse		CTCTTCTGTGCTCTTGACGA
GRIN2A Forward	qPCR	CTGTGAAGTTTACTTACGACCTC
Reverse		GTTGATAGACCACTTCACCGA
GRIN2B Forward	qPCR	TGCCTTCTTAGAGCCATTCAG
Reverse		GTACTCAAAGACAAAGACAGCC
GRIN3A Forward	qPCR	CATTTGCCATAGAAGGATACGG
Reverse		CCCATGTGACTTGTATTGACTG
HNF1B (Exon 2) Forward	qPCR	CATCACCTGTGGGCTCTTCAA
Reverse		CCTCCGACAATTCAACCAGAC
HNF1B (Exon 9) Forward	qPCR	TTCCATCTGCAATGGTGGTC
Reverse		CAGGCTTGTAGAGGACACTG
sgHNF1B-231(+) coding	Insert creation	CACCGGCCGCTTGTCCGGCGACGA
complementary		AAACTCGTCGCCGGACAAGCGGCC
sgHNF1B-171(-) coding	Insert creation	CACCGAGAGTATGGAAGACCGGCT
complementary		AAACAGCCGGTCTTCCATACTCTC
Genotyping *HNF1B* Exon-1 deletion Forward	PCR	TTTCTGACTCCTTTCGGAGGA
Reverse		AAGTCGCAGCGGTTTCACTG
Sequencing of Exon-1 deletion PCR fragment	Sequencing	GGGTTTGCTTGTGAAACTCC
Genomic PCR *HNF1B*-exon 9 (5’ end of exon) Forward	qPCR	GTGTCCTCTACAAGCCTGGT
Reverse	qPCR	CAGAGGGTGATGGTGTGGA
Genomic PCR *HNF1B*-exon 9 (3’ end of exon) Forward	qPCR	GTTGAGTTGGGCATCATCTCC
Reverse	qPCR	ATCACCAGGCTTGTAGAGGAC

Table S3. (Related to Experimental Procedures). Antibodies and lectins used in immunohistochemistry (IHC), immunocytochemistry (ICC) and western blot (WB) experiments.

**Primary Antibody**	**Host**	**Source**	**Catalogue #**	**Application**	**Dilution**
Activated Caspase-3	Rabbit	Abcam	ab2302	IHC	1:250
BrdU	Rabbit	BioRad	AHP2405	IHC	1:100
CUBN	Goat	Santa Cruz	sc-20607	IHC	1:100
CDH1	Mouse	Abcam	ab76055	IHC	1:1000
				ICC	1:200
GAPDH	Rabbit	Cell Signalling	5174	WB	1:1000
GRIK3	Rabbit	Thermofisher	PA5-98452	IHC	1:800
				WB	1:1000
HNF1B	Rabbit	Atlas Antibodies	HPA002083	IHC	1:2000
				ICC	1:200
				WB	1:1000
Megalin	Mouse	Novus Biologics	NB110-96417	IHC	1:200
PECAM1 (CD31)	Mouse	Cell Signalling	3528	IHC	1:100
SYNPO	Mouse	Santa Cruz	sc-50459	IHC	1:200

**Secondary Antibody**	**Host**	**Source**	**Catalogue #**		**Dilution**
Anti-Mouse IgG AlexaFluor-594	Donkey	Thermo Fisher Scientific	A21203	ICC	1:300
Anti-Rabbit IgG AlexaFluor-488	Donkey	Thermo Fisher Scientific	A21206	ICC	1:300
Biotinylated anti-Mouse IgG	Horse	Vector Laboratories	BA-2000	IHC	1:400
Biotinylated anti-Rabbit IgG	Goat	Vector Laboratories	BA-1000	IHC	1:400
Biotinylated anti-Goat IgG	Horse	Vector Laboratories	BA-9500	IHC	1:400
IRDye® 800CW Anti-Rabbit	Donkey	LI-COR Biosciences	926-32213	WB	1:15000

**Lectins**		**Source**	**Catalogue #**	**Application**	**Dilution**
Biotinylated LTL		2B Scientific	B-1325-2	IHC	1:400

Table S4. (Related to Figure 4). KEGG pathway enrichment analysis at different points of organoid differentiation, comparing non-mutant and mutant organoids.

	KEGG ID	Description	p-adjusted	Gene count (fraction of total in pathway)
Day 12	hsa05016	Huntington disease	0.004474	6/49
	hsa00480	Glutathione metabolism	0.00578	2/10
	hsa04723	Retrograde endocannabinoid signaling	0.009009	5/29
	hsa04022	cGMP-PKG signaling pathway	0.011086	6/30
	hsa04550	Signaling pathways regulating pluripotency of stem	0.018149	11/33
	hsa04915	Estrogen signaling pathway	0.02521	5/17
	hsa04921	Oxytocin signaling pathway	0.030172	9/24
	hsa04142	Lysosome	0.03125	2/16
	hsa04724	Glutamatergic synapse	0.0375	4/18
	hsa05012	Parkinson disease	0.039387	11/44
	hsa05010	Alzheimer disease	0.047826	7/42
	hsa04714	Thermogenesis	0.048458	11/61
Day 19	hsa00562	Inositol phosphate metabolism	0.002604	1/10
	hsa04917	Prolactin signaling pathway	0.002732	4/16
	hsa04934	Cushing syndrome	0.003021	10/33
	hsa05225	Hepatocellular carcinoma	0.003067	15/35
	hsa05226	Gastric cancer	0.003165	18/43
	hsa05224	Breast cancer	0.003344	19/45
	hsa04666	Fc gamma R-mediated phagocytosis	0.005208	2/13
	hsa05217	Basal cell carcinoma	0.00551	6/19
	hsa04916	Melanogenesis	0.00554	6/21
	hsa04310	Wnt signaling pathway	0.006452	17/44
	hsa04012	ErbB signaling pathway	0.006515	1/14
	hsa04070	Phosphatidylinositol signaling system	0.007813	1/13
	hsa04740	Olfactory transduction	0.008086	39/77
	hsa04150	mTOR signaling pathway	0.009202	6/35
	hsa04020	Calcium signaling pathway	0.012658	13/43
	hsa04714	Thermogenesis	0.014035	48/62

	hsa05218	Melanoma	0.016854	10/18
	hsa05165	Human papillomavirus infection	0.017241	17/61
	hsa04530	Tight junction	0.019293	9/29
	hsa05205	Proteoglycans in cancer	0.022581	8/44
	hsa00190	Oxidative phosphorylation	0.023411	35/48
	hsa04976	Bile secretion	0.042071	5/13
	hsa03010	Ribosome	0.04908	27/38
Day 25	hsa00190	Oxidative phosphorylation	0.002257	39/48
	hsa03010	Ribosome	0.004484	34/39
	hsa04024	cAMP signaling pathway	0.004535	10/58
	hsa04714	Thermogenesis	0.006726	41/62
	hsa05010	Alzheimer disease	0.008696	33/43
	hsa04923	Regulation of lipolysis in adipocytes	0.01073	2/13
	hsa05140	Leishmaniasis	0.010846	8/17
	hsa05164	Influenza A	0.011062	13/38
	hsa04932	Non-alcoholic fatty liver disease (NAFLD)	0.011211	24/39
	hsa04917	Prolactin signaling pathway	0.023861	2/17
	hsa00230	Purine metabolism	0.025263	7/24
	hsa04940	Type I diabetes mellitus	0.029279	5/10
	hsa04080	Neuroactive ligand-receptor interaction	0.030879	46/148
	hsa04623	Cytosolic DNA-sensing pathway	0.035955	4/11
	hsa04380	Osteoclast differentiation	0.037815	3/20
	hsa04970	Salivary secretion	0.037815	10/20
	hsa04740	Olfactory transduction	0.039927	37/76
	hsa05224	Breast cancer	0.043182	17/47

Table S5. (Related to Figure 4). Differentially expressed genes between *HNF1B*-mutant and non-mutant control organoids which are associated with genetic diseases of the kidney (Online Mendelian Inheritance In Man® database (OMIM®).

Gene	Description	Disease	OMIM code	Structure affected[a]	Change in mutant[b] Day 19	Day 25
NPHS2	Podocin	Nephrotic syndrome, type 2	600995	G	↑	
CLCN5	Chloride channel	Dent disease 1	300009	PT		↓
LRP2	Megalin	Donnai-Barrow syndrome	222448	PT	↓	↓
SLC34A1	Na^+/phosphate cotransporter	- Hypophosphatemia<br>- Renal Fanconi syndrome 2	612286<br>613388	PT<br>PT	↓	↓
SLC4A4	Na^+/bicarbonate cotransporter	PT acidosis	604278	PT		↓
CLDN19	Tight junction protein	hypomagnesemia type 5	248190	DT		↓
KCNJ16	Potassium channel	Hypokalemic nephropathy and deafness	619406	DT		↓
MUC1	Mucin	Medullary cystic kidney disease	174000	DT		↓
SLC12A1	$Na^+/K^+/Cl^-$ transporter	Bartter syndrome type 1	601678	DT		↓
SCNN1G	Na^+ channel	- Liddle syndrome<br>- Pseudohyoaldosteronism type IB3, autosomal recessive	618114<br>620126	CD		↑
MET	HGF receptor	Renal cell carcinoma, papillary, 1, familial and somatic	605074	Tub		↓
PKHD1	Fibrocystin	Polycystic kidney disease 4, with or without hepatic disease	263200	Tub	↓	↓

[a] G: glomerulus; PT: proximal tubules ; DT: distal tubules; CD: collecting ducts; Tub: Wide tubular expression.

[b] Time points during differentiation when expression differs significantly are indicated: Upwards arrows denote a significant upregulation in the mutant and downward arrows a significant downregulation (p-adjusted<0.01).

Table S6. (Related to Figures 5 and 7). Marker genes used for the annotation of cell clusters in scRNAseq.

	Cell grouping name	Marker genes
1	Podocyte	NPHS1, NPHS2, WT1, PODXL, ROBO2
2	Podocyte	NPHS1, NPHS2, WT1, PODXL, ROBO2
3	Podocyte (Proliferating)	NPHS1, NPHS2, WT1, PODXL, ROBO2, TOP2A, CDK1, PCNA
4	GRIK3+ Nephron progenitor cells	PAX8, PAX2, LHX1, WT1, GRIK3
5	Nephron progenitor cells	PAX2, PAX8, LHX1, WT1
6	Nephron progenitor cells	PAX2, PAX8, LHX1, WT1
7	Nephron progenitor cells (Proliferating)	PAX2, PAX8, LHX1, WT1, TOP2A, CDK1, PCNA
8	GRIK3+ distal nephron cells	HNF1B, EPCAM, PAX2, PAX8, LHX1, GRIK3
9	Distal nephron cells	HNF1B, EPCAM, PAX2, GATA3
10	Distal nephron cells (Proliferating	HNF1B, EPCAM, PAX2, TOP2A, CDK1, PCNA
11	Thick ascending limb	SLC12A1, HNF1B, EPCAM, CDH1
12	Proximal tubular cells	LPR2, CUBN, HFN1A, SPP1, HNF1B
13	Mesenchymal (low RP)	MEIS2, PDGFRA, PDGFC
14	PDGFC+ mesenchymal	MEIS2, PDGFRA, PDGFC, PDGFD
15	Mesenchymal	MEIS2, PDGFRA
16	Mesenchymal	MEIS2, PDGFRA, MEIS1
17	Mesenchymal	MEIS2, PDGFRA
18	Mesenchymal (Proliferating)	MEIS2, PDGFRA, PDGFC, TOP2A, CDK1, PCNA
19	Muscle cells	MYOG, MYLPF
20	Neural progenitor cells	CRABP1, MAP2
21	Neuronal cells	ELAVL2/3/4, CRABP1, MAP2
22	Melanocytes	PMEL, MITF
23	Endothelial cells	PECAM, KDR

SUPPLEMENTAL EXPERIMENTAL PROCEDURES

Sources of human tissues

First trimester fetal kidneys collected after maternal consent and ethical approval (ethics REC 08/H0906/21+5; and REC 18/NE/0290) and were provided by the MRC and Wellcome Trust Human Developmental Biology Resource (http://www.hdbr.org/). Third trimester human fetal kidneys, were used for research histology studies with approval from the Ethics committee of the Hôpital Robert Debré, as detailed previously (Haumaitre et al., 2006). To generate hiPSCs, venous blood samples were obtained with informed consent from a family with inherited HNF1B-associated DKMs (Manchester Gene Identification Consortium Study (REC 11/H1003/3; IRAS ID 64321).

hPSC cell culture

MAN13 is a clinical grade hESC line (Ye et al., 2017), which has been well characterised (https://hpscreg.eu/cell-line/UMANe002-A) and was derived under local ethical approval (Central Manchester LREC favourable opinion 03/CM/684) and UK Human Fertilisation and Embryology Authority license (HFEA licence R0171) with fully informed parental consent. Stem cells were grown on culture plates coated with 5 µg ml^{-1} recombinant human Vitronectin (rhVTN-N, Life Technologies, #A14700). HESCs were grown in mTeSR1 (StemCell Technologies, #85850) medium, whereas iPSCs were grown in TeSR-E8 (StemCell Technologies, #05990), with the medium changed every two days. The cells were passaged by treatment of the cultures with 0.5mM EDTA solution, pH8 (Invitrogen, #15575-038; diluted in PBS) and replating the cells in medium containing 5µM ROCK inhibitor, Y-27632 (Tocris, #1254) for 24h.

CRIPSR/Cas9^n editing of hESCs

We first constructed a pair of plasmids expressing the nickase (D10A) version of Cas9 (Cas9^n) and a gRNA each. The two gRNAs targeted the Cas9^n to nick at position 231 of the coding strand of *HNF1B* and at position 171 of the complementary strand, resulting in a deletion of 58 bases (plus/minus any indels) near the beginning of the coding sequence of the gene (Fig. 1A). Each gRNA-coding insert was designed as a pair of oligonucleotides that, upon annealing, produced overhangs that allowed cloning into the pX461 plasmid vector, (Addgene, #48140), under the control of a U6 promoter. The vector also expresses Cas9^n, and a GFP tag for cell sorting. The primers used to create the inserts are listed in Table S2. Each pair of oligonucleotides was annealed and phosphorylated, using T4 Polynucleotide Kinase (NEB, #M0201S), then ligated into BbsI-digested pX461, to produce pX461-gRNA(HNF1B/231+) and pX461-gRNA(HNF1B/177-). $4x10^5$ MAN13 hESCs were nucleofected with both pX461-gRNA(HNF1B/231+) and pX461-gRNA(HNF1B/177-), using the Amaxa™ P3 Primary Cell 4D-Nucleofector™ X Kit L (Lonza, #V4XP-3024), in 100µl nucleofection buffer according to the manufacturer's instructions, on a Lonza 4D-Nucleofector (program DN100). 1ml of TeSR1 medium, containing 10 µM ROCK inhibitor, was then added to the cell mix and the cells were plated on a well of a 12-well plate coated with Vitronectin. The medium was replaced 16h later with 1ml fresh TeSR1 medium. Two days post-nucleofection, the cells were collected by TrypLE treatment (Life Technlogies, #12605-028) for 2min at 37°C and sorted for GFP fluorescence on a BD FACSAria Fusion flow cytometer. Two aliquots of the sorted, GFP+ cells of 5,000 and 10,000 cells were then plated in one Vitronectin-coated well each in a 6-well plate. The rest of the GFP+ cells (approximately 35,000) were plated on a separate well and, when confluent, they were stored in liquid N_2, as backup. After 8-15 days, separate cell colonies had emerged on the sparsely seeded plates. Each was manually passaged, using a flame-pulled glass Pasteur pipette, into a separate well of a 24-well plate. Each clonal line thus produced was expanded further by EDTA passaging when confluent, and genomic DNA (gDNA) was extracted for genotyping using the Wizard® Genomic DNA Purification Kit (Promega, #A1120). A 781bp fragment around the expected mutation site was amplified by PCR, using Herculase II polymerase (Agilent, #600675) and was then sequenced to verify the presence/absence of the deletion. The primers used for PCR amplification and sequencing are detailed in Table S2.

iPSC derivation

To generate hiPSCs, venous blood samples were obtained with informed consent from a family with inherited *HNF1B*-associated DKMs (Manchester Gene Identification Consortium Study (REC 11/H1003/3; IRAS ID 64321). Three siblings, each from a separate pregnancy, had bilateral DKMs detected on fetal ultrasonography. The female sibling had oligohydramnios and underwent termination because of a poor prognosis. Her two male siblings were born and were found to have ultrasound-bright kidneys, with loss of distinction between the cortex and medulla, core sonographic features of DKMs. When assessed as young adults, their estimated glomerular filtration rates were modestly decreased (60-70 ml/min), and each had evidence of tubulopathy, e.g. hyperuricaemia, with one brother also having urinary glucose wasting. Each brother carries a deletion of exon 9 of *HNF1B* (del c.1654-? – c.1674-?; abbreviated as $HNF1B^{+/\Delta Exon9}$) inherited from their father who has kidney disease and gout.

A total of 4 ml of blood were withdrawn and transferred to BD Vacutainer™ Hemogard closure plastic K2-EDTA tubes (BD 367525, BD). Tubes were inverted 10 times to ensure blood and EDTA were well mixed and kept at room temperature (RT) until processing. Peripheral blood mononuclear cells (PBMCs) were isolated by first mixing the each blood sample with an equal volume of PBS. An equal amount of Ficoll® Paque Plus (GE17-1440-02, Sigma) was then added in a 15 ml Falcon tube and the blood/PBS mix was carefully layered over the Ficoll reagent and centrifuged at 400g for 40 min, at RT. A total of 1ml PBMCs was isolated using a sterile Pasteur pipette and transferred into a new 15ml Falcon tube containing 9 ml of PBS. Diluted PBMCs were centrifuged at 200g for 10 min. The supernatant was discarded and the pellet was washed with 10 ml of PBS. Centrifugation was repeated at RT and the supernatant were discarded. PBMCs were resuspended in 2ml (0.5 ml medium per ml of starting blood) of Erythroid expansion medium, StemSpan™ SFEM II (#09605, STEMCELL Technologies) supplemented with StemSpan™ Erythroid Expansion Supplement (100X) (#02692, 50 STEMCELL Technologies). Typically, $1x10^6$ PBMCs were recovered per ml of blood. To expand the PBMC population, $5x10^5$ PBMC were plated in one well of a 6-well plate, containing 2 ml of erythroid expansion medium and incubated overnight. The following day, all non-adherent cells were transferred to a new plate and incubated overnight, while adherent cells were discarded. The cells were incubated and allowed to grow for a further 6 days, being fed every two days by carefully removing 1.5 ml of used erythroid expansion medium and replaced with fresh medium. The cells were then transduced with CytoTune™-iPS 2.0 Sendai Reprogramming Kit (A16517, Thermo Fisher), according to the manufacturer's instructions. Briefly, per sample, $5x10^4$ cells were pelleted by centrifugation at 300g and were transduced by resuspension in 250micro µl erythroid expansion medium containing Sendai vectors (SeV) at a multiplicity of infection (MOI; viral particles per cell) of 5 for SeV-hKOS, 5 for SeV-hc-Myc and 3 for SeV-hKlf4. Cells were then centrifuged at 300g for 35 min to assist transduction, at RT, then resuspended and plated into two wells of a 24-well plate, where they were allowed to expand for a further four days, in a total volume of 600µl per well. The cells in each well were then transferred to a Vitronectin-coated well of a 6-well plate, containing 0.5 ml of ReproTeSR™ medium (#05921, STEMCELL Technologies). After two days, an extra 1ml of medium was added per well and the cells were allowed to settle down over the next 15 days, during which time iPSC colonies began to emerge. During this time, the medium was replaced with 1.5 ml ReproTeSR™ every 2 days. Colonies were manually cut into several pieces, using a pulled glass pipette, and each transferred to a Vitronectin-coated well of a 6-well plate. They were then passaged either manually, using a pulled glass pipette as above, or by EDTA treatment (see below), until approximately passage 20 before being used in experiments.

2D and organoid differentiation

2D and organoid differentiation was performed by an adaptation of previously described protocols (Takasato et al., 2015; Bantounas et al., 2018). In both protocols, hPSCs were plated on vitronectin-coated plates (see "hPSC culture" section) at a density of 18,000 cells cm^{-2} in mTeSR1 medium (for hESCs) or TeSR-E8 medium (for iPSCs) containing 10µM ROCK inhibitor Y-27632. Differentiation was initiated the following day ("Day 0" of the protocol) by replacing the medium with STEMdiff™ APEL™2 medium (StemCell Technologies, #05270), supplemented with 1% (v/v) PFHM-II Protein-Free Hybridoma Medium (Thermo Fisher Scientific, # 11370882), containing 8 µM CHIR-99021 (Tocris, #4423). APEL™2 with PFMH-II is hereafter referred to as "base medium".

For 2D differentiation experiments, the medium was replaced daily, until Day 3 of the protocol, when it was replaced with base medium containing 200 ng ml^{-1} FGF9 (Peprotech, #100-23) and 1 µg ml^{-1} heparin (Sigma, #3149). Daily feeds continued with this medium until day 13, when the cells were fixed for immunocytochemistry (see below).

For organoid differentiation, CHIR-containing medium was used until Day 4 of the protocol, at which point it was replaced by FGF-9/heparin-containing medium. Daily feeds continued until Day 7 of the protocol. At Day 7, cells were dissociated by TrypLE treatment for 3-5min, centrifuged at 700g and resuspended in base medium. The suspension was separated into 1.5ml eppendorfs, in aliquots of 2x10^5 cells, which were then centrifuged at 400g for 2 min. The resultant cell pellets/organoids were transferred onto MilliCell cell culture inserts (0.4µm pore size; Millipore, #PICM03050), with three organoids place on each insert. The inserts had previously been placed on APEL™ containing 5µM CHIR-99021. Following a 1h incubation, the medium was replaced with FGF9/Heparin-containing APEL™. From this point on, the medium was replaced every two days. On Day 12, the medium was replaced with just base medium until the end of the protocol on Day 25.

RNA isolation from organoids and human embryonic tissue

RNA from kidney organoids was collected at day 0, 7, 12, 19 and 25 of the differentiation protocol. RNA was extracted using the miRVana miRNA isolation kit (Thermo Fisher, AM1560) according to the manufacturer's instructions. Three organoids were pooled and lysed together to produce each sample. Four human felt kidneys (8, 8, 9 and 10 weeks of gestation) were obtained frozen and were homogenized on dry ice in Eppendorf tubes, using sterile plastic mini-pestles. Homogenized tissue was lysed in 600µl Lysis Buffer (provided with the miRVana kit) and RNA was then isolated using the miRVana kit according to the manufacturer's instructions.

Quantitative PCR

Quantitative real-time PCR was performed using the TaqMan® RNA-to-Ct™ 1-Step Kit (Thermo Fisher, #4392653) according to the manufacturer's instructions, on a BioRad C1000™ Thermal Cycler fit with a CFX384™ Real Time System, using 15ng of RNA per reaction. The primers used are shown in Table S2.

Next Generation RNA sequencing (RNAseq).

IBM13-08 and IBM13-19 hESCs were differentiated as described above in three independent experiments and RNA was collected as described above at days 0, 7 12, 19, 25 of differentiation. RNAseq was performed by the University of Manchester Genomics Facility, using an Illumina HiSeq4000 sequencer and unmapped paired-end sequences FastQC v0.11.3 (http://www.bioinformatics.babraham.ac.uk/projects/fastqc/). Sequence adapters were removed, and reads were quality trimmed using Trimmomatic v0.39 (Bolger et al., 2014). The reads were mapped against the reference human genome (hg38) and counts per gene were calculated using annotation from GENCODE 36 (http://www.gencodegenes.org/) using STAR v2.7.7a (Dobin et al., 2013). Normalisation, Principal Components Analysis, and differential expression was calculated with DESeq2 v1.36.0 (Love et al., 2014) in R v4.2.0 using default settings. Adjusted p-values were corrected for multiple testing (Benjamini and Hochberg method). Heatmaps were drawn with complexHeatmap v2.12.1 (PMID: 27207943).

Single-cell RNA sequencing (scRNAseq)

Day 25 organoids were washed twice with PBS and three organoids were pooled together per sample and resuspended in 1 mL of TrypLE™ at 37°C for 15 minutes. They were then dissociated to single cells by passing through a 27G needle (BD Biosciences, #305540). The suspension was centrifuged at 400xg for 3 minutes and the cell pellet resuspended in 1 mL of basal medium. Cells were diluted into

1000 cells per µl and 16 µl of the diluted cell suspension and 30.6 µL of nuclease-free water were used per reaction. Gene expression libraries were prepared from single cells using the Chromium Controller and Single Cell 3' Reagent Kits v3.1 (10x Genomics, Inc. Pleasanton, USA) according to the manufacturer's protocol (CG000315 Rev B). The resulting sequencing libraries comprised standard Illumina paired-end constructs flanked with P5 and P7 sequences. Paired-end sequencing (26:98) was performed on the Illumina NextSeq500 platform using NextSeq 500/550 High Output v2.5 (150 Cycles) reagents. The .bcl sequence data were processed for quality control purposes using bcl2fastq software (v. 2.20.0.422) and the resulting .fastq files assessed using FastQC (v. 0.11.3), FastqScreen (v. 0.9.2) and FastqStrand (v. 0.0.5) prior to processing with the CellRanger pipeline (v7.0.0). in Orchestrating Single-Cell Analysis with Bioconductor (Amezquita et al., 2020).

The log-normalised expression values of the combined data were re-computed using the "multiBatchNorm" function from the batchelor R package (v1.10.0). The per-gene variance of the log-expression profile was modelled using the "modelGeneVarByPoisson" function and the top 3000 highly variable genes (HVGs) were identified using the "getTopHVGs" function both from the scran R package (v1.22.1). The mutual nearest neighbors (MNN) implemented by the "fastMNN" function from the batchelor R package was used to perform batch correction of scRNA-seq data.

The first 50 dimensions of the MNN low-dimensional corrected coordinates for all cells were used as input to produce the uniform manifold approximation and projection (UMAP) using the "runMAP" functions from the scater R package (v1.22.0). Graph-based clustering was performed using the Leiden algorithm from the igraph R package (v1.3.0) to identify communities of cells. Specifically, the "clusterRows" function from the bluster R package (v1.4.0) was used to perform the clustering procedure.

Western blotting

Per sample, three organoids were pooled and homogenised in 350 µL of RIPA lysis buffer (Thermo fisher Scientific, #89900) with protease and phosphatase inhibitors. Twenty to thirty µg of sample was loaded per well, on a NuPAGE™ 10% Bis-Tris, 1.0 mm, Mini protein Gels (10-well) (Thermo fisher Scientific, #NP0301) and electrophoresed in 1x MES buffer (diluted from a 20X stock; Thermo fisher Scientific, #NP0002). Proteins were transferred onto a nitrocellulose membrane in the iBlot2 transfer stack (Thermo fisher Scientific, #IB23001) using an iBlot 2 Dry Blotting System (Life Technologies). Membranes were blocked with 5% skimmed milk powder diluted in PBS-Tween 0.1% (v/v) (Sigma-Aldrich, #PP9416) (PBS-T), for 1 hour at room temperature with agitation. Primary antibodies (Table S3) were diluted in blocking solution and incubated with the membrane, at 4°C with agitation overnight. The membrane was then washed in PBS-T 3 times for 10 minutes and secondary antibodies (Table S3), diluted in blocking solution, were incubated for 1 hour at room temperature, followed by three final 10-minute PBS-T washes. Membranes were imaged with the Odyssey CLx Imaging system (LI-COR Biosciences, Germany) and densitometry was performed with the Image Studio Lite quantification software.

Immunocytochemistry of 2D cultures

Cells were washed twice in PBS and then fixed in 4% paraformaldehyde (PFA) for 20 minutes, followed by another two PBS washes. The fixed cells were blocked and permeabilised for 30 min with 3% bovine serum albumin (BSA)/0.3% Triton-X in PBS before overnight incubation at 4˚C with primary antibodies (Table S3) diluted in 3% BSA/PBS. They were then washed three times with PBS/0.1% Triton-X, followed by Alexa-Fluor™-488- or Alexa-Fluor™-594-labelled, species-specific secondary antibodies (Life Technologies; 1:300 dilution in 3% BSA/PBS). Images were collected on a Zeiss Axioimager.D2 upright microscope using a 63x/Plan-neofluar objective and captured using a Coolsnap HQ2 camera (Photometrics) through Micromanager software v1.4.23. Images were then processed and analysed using Fiji-ImageJ (http://imagej.net/Fiji/Downloads).

Immunohistochemistry of organoid sections

Organoids were fixed in 4% paraformaldehyde, embedded in paraffin and sectioned at 5 µm. Sections were dewaxed and rehydrated, and alternate slides were stained with haematoxylin and eosin (H&E) to assess overall tissue architecture. Images were acquired on a 3D-Histech Pannoramic-250 microscope slide-scanner using a x20 objective (Zeiss) and selected images were captured using the Case Viewer software (3D-Histech). After rehydration, other slides were boiled in an 800W microwave in 10 mM sodium citrate buffer (pH 6.0). After cooling to room temperature, endogenous peroxidase activity was blocked using 0.3% H2O2 in PBS for 10 minutes. Sections were permeabilized using 0.2% Triton X-100 (Sigma-Aldrich) for 10 minutes and blocked using 1% bovine serum albumin (BSA) with 10% serum from the species in which the secondary antibody was raised. Sections were incubated overnight at 4°C with the primary antibody + 1% BSA. Primary antibodies used are listed in Table S3. Biotin-conjugated species specific secondary antibodies with 1% BSA were incubated at room temperature for 2 hours. Following PBS washes, slides were incubated in avidin-biotin enzyme complex (Vector Laboratories VECTASTAIN Elite ABC Reagent, PK-6100) for 1 hour at room temperature. Peroxidase activity was detected with the 3, 3'-diaminobenzidine (DAB) peroxidase substrate solution (Vector Laboratories, SK4100) in some cases with haematoxylin counterstain. Sections were dehydrated and mounted with DPX mounting medium and examined under a Leica DMLB 2 microscope. Negative controls omitted primary antibodies. For immunofluorescent imaging, anti-rabbit Alexa-Fluor™ 488 (Thermo Fisher Scientific, # A11034) and anti-mouse Alexa-Fluor™ 594 (Thermo Fisher Scientific, #A11032) secondary antibodies and DAPI nuclear stain were used. Sections were mounted with Vectashield antifade mounting medium (Vector Laboratories, #H-1000). Images were acquired using an Olympus BX63 upright microscope using a DP80 camera (Olympus) through CellSens Dimension v1.16 software (Olympus) and slide scanned on a 3D-Histech Pannoramic-250 microscope slide-scanner using a 40x/0.95 Plan Apochromat objective (Zeiss) and captured using the Case Viewer Pannoramic250 slide scanner software (3D-Histech) at the University of Manchester Bioimaging facility, and processed and analysed using Fiji-ImageJ (http://imagej.net/Fiji/Downloads).

Immunohistochemistry and periodic acid-Schiff (PAS) staining of fetal kidneys

Sections were dewaxed and rehydrated, endogenous peroxidase activity was blocked using 0.3% H2O2 in PBS for 20 minutes with agitation. Sections were then boiled in a microwave in 10micro mM sodium citrate buffer (pH 6.0). After cooling to room temperature, they were incubated with saturated lithium carbonate cat. 26684-03 (Generon Ltd) in distilled water for 30 minutes, permeabilised with Tris-buffered saline (TBS)/triton X-100 0,3% for 20 minutes followed by incubation in blocking buffer for 45 minutes in a humidified chamber at room temperature. Primary antibody GRIK3 (Table S3) was diluted in 1:800 and tissues were incubated overnight at 4°C. After washing, biotin-conjugated secondary antibody was incubated at room temperature for 1 hour. Following washes, slides were incubated in avidin-biotin enzyme complex for 30 minutes at room temperature and peroxidase activity was detected as above. Periodic acid-Schiff stain (PAS) was carried out according to manufacturer's instructions using the kit 395B (Scientific Laboratory Supplies Ltd). Images were captured as above.

In situ RNA hybridisation (BaseScope™)

Organoids were fixed in 4% paraformaldehyde, paraffin-embedded and sectioned at 5 µm. BaseScope *in situ* hybridisation (ACDBio, Newark, CA, USA) was adapted from Lopes et al (Lopes et al., 2021), and conducted following the manufacturer's instructions, using the BaseScope detection reagent Kit v2-RED. RNA (red) was detected using Fast RED and nuclei counterstained with Gill's haematoxylin. The following custom-made BaseScope probes were used: BA-Hs-*HNF1B*-3zz-st targeting 1078-1252; BA-Hs-*HNF1A*-3zz-st targeting 1453-1565 and BA-Hs-*GRIK3*-No-XMm-3zz-st, targeting 3258-3414 and the following control probes: human (HS)-*PPIB-3zz* cat. 701031 (positive control); and *DapB* (bacterial gene)-*3ZZ* cat.701011 (negative control). The positive control probe was the widely expressed *PPIB* transcript encoding peptidylprolyl isomerase B, and a negative control probe was *DapB* encoding 4-hydroxy-tetrahydrodipicolinate reductase from the *Bacillus subtilis* soil bacterium, a gene that is absent in mammals. Images were acquired using an Olympus BX63 upright microscope using a DP80 camera (Olympus) through CellSens Dimension v1.16 software (Olympus).

cAMP-induced tubule dilatation

We used a previously described protocol used for intact metanephroi maintained in organ culture (Anders et al., 2013). The growth medium of organoids was supplemented with 100μM with 8-Bromoadenosine 3',5'-cyclic monophosphate sodium salt (8-Br-cAMP) (Sigma, #B7880) in iPSC organoids or 200μM forskolin (FSK) (Tocris, #1099) in hESC organoids, starting on day 14 of the protocol. Treatment continued until day 25 (iPSC organoids) or day 32 (hESC organoids). Dilatations were identified by their translucent/bright appearance under phase microscopy. Their number and size were measured using Fiji-ImageJ.

Cell Proliferation Assay

Day 25 differentiated kidney organoids were incubated with 10 µM of 5-Bromo-2'-deoxyuridine (BrdU) (Sigma-Aldrich, #B5002-100MG) in basal medium for 2 hours at 37°C, by placing 1.2ml BrdU supplemented medium underneath the transwell filter and 1ml inside the transwell. Organoids were then fixed and processed and immunohistochemistry was used to stain BrdU positive nuclei.

GO and KEGG pathway analysis

Gene ontology enrichment was studied using Enrichr v3.1 (PMID: 27141961). Enriched KEGG (Kanehisa et al., 2017) and Reactome (Fabregat et al., 2016) pathways were identified using the clusterProfiler package (Yu et al., 2012) and visualized using the pathview package (Luo and Brouwer, 2013). For Gene Set Enrichment Analysis (GSEA), a pre-ordered list of log_2fold changeFC values, and for Over Representation Analysis (ORA), an input list of DEGs was used to get enriched pathways. Up-regulated pathways were defined by a normalized enrichment score (NES) > 0 and the down-regulated pathways were defined by an NES < 0. Pathways with BH adjusted-P value ≤ 0.05 were chosen as significantly enriched.

In silico identification HNF1B-regulated promoters

Transcription factor (TF), HNF1B-responsive genes were searched using iRegulon (Janky et al., 2014). iRegulon detects the TFs and their targets by scanning known TF-binding promoter motifs as well as the predicted motifs discovered from the Encyclopedia of DNA Elements (ENCODE) Project chromatin immunoprecipitation-sequencing data. It includes 1,121 human regulatory tracks with ChIP-seq data for 247 sequence-specific TFs across 43 different cell types and conditions. We selected 20-kb upstream parameter for the options "Putative regulatory region," "Motif rankings database," and "Track rankings database" to identify the targets. Motif sequences of HNF1B were explored with in silico predicted and experimentally validated DNA-binding motifs collection databases (JASPAR2018_CORE_vertebrates_non-redundant, Jolma 2013, TRANSFAC, UniPROBE mouse, HOCOMOCO v11 and Swiss Regulon) using TOMTOM (Gupta et al., 2007) (e-value 0.5; min overlap between motifs).

Quantification and statistical analysis

qPCR

For quantification of *HNF1B* mRNA expression levels, RNA from four independent differentiation experiments was assessed by qPCR. *GAPDH* expression levels were used as a loading control, to normalise *HNF1B* readings. Each qPCR reaction was performed in triplicate on the reaction plate and one outlier was removed if the standard deviation (S.D.) of the average C_t values exceeded 0.250 and this could bring the S.D lower than 0.250. Average *GAPDH* C_t values were then subtracted from corresponding average *HNF1B* C_t values (yielding ΔC_t values) to normalise for loading. Results were finally expressed as $2^{-\Delta Ct}$.

Western Blots

Membranes were imaged with the Odyssey CLx Imaging system (LI-COR Biosciences, Germany) and densitometry was performed with the Image Studio Lite quantification software (installed with the machine). Readings for the amounts of HNF1B and GRIK3 were normalised against those of GAPDH (housekeeping gene).

Organoid size

Organoid size comparison between day-25 mutant and non-mutant organoids was performed using Fiji-ImageJ on phase images of organoids, measuring the total area in each image. In total, 12 non-mutant and 11 mutant organoids from three independent differentiation experiments were measured. Each dot on the graph represents a separate organoid.

Lumen and epithelial area of LTL+ and CDH1+ tubules

Following sectioning of each paraffin-embedded organoid, the middle section was chosen for staining with either LTL lectin or an anti-CDH1+ antibody. Of the LTL+ or CDH1+ tubules, only the ones that lay perpendicular or near-perpendicular to the sectioning plain were assessed. This was judged by tracing the outline of the cross-section of the tubule, using Fiji-ImageJ, and calculating its circularity, using the formula $c=4\pi*(A/P^2)$, where c is the circularity, A is the area of the traced shape and P its perimeter. Only tubule cross-sections with $c>0.85$ were analysed. Once the tubules were selected using the above criterion, we used Fiji-ImageJ to encircle each such tubule and its lumen (see Figure 2G for a schematic representation). When presenting the results, “Total area” corresponds to the shape encircling all of the tubule, “Lumen” to the empty lumen area in the centre and “Epithelium” to the “Total” minus the “Lumen”. In total, per experimental group, nine organoids from three independent differentiation experiments were quantified this way. Each dot on the graph corresponds to a different organoid and represents the average value of all tubules measured for that organoid.

BrdU+ and active-Caspase3+ cells

Total and BrdU+ nuclei were counted in tubular epithelia of mutant and non-mutant organoid sections. One section per organoid was selected, within 3 sections of the most central section, and nuclei were counted manually on all tubules of the section. Eight organoids for the non-mutant group and 7 from the mutant group were used, from three independent differentiation experiments. The results were expressed as a percentage of BrdU+ to total nuclei in all tubules of each section. Each dot on the graph (Figure S1N) represents a separate organoid.

Images of activated-Caspase3-immunostained sections previously captured were captured using a 3D-Histech Pannoramic-250 microscope slide-scanner using a 40x/0.95 Plan Apochromat objective (Zeiss) at the University of Manchester Bioimaging facility (see “Immunohistolchemistry of organoid section” section above) were loaded onto the Caseviewer software (ver 2.2; 3DHISTECH Ltd.). For each section, we captured images (using the 20x virtual magnification objective of the software) in a line from top to bottom of the section, passing through its centre. This typically yielded between 4 and 8 images per section. For each image, we used the ImageJ-Fiji “Color Deconvolution” function and selected the “H DAB” option in order to separate the Caspase3 staining (brown) from the haematoxylin background. Positive cells in the tubular epithelia were then counted and expressed as a percentage of total cells in tubular epithelia. In total, one section from each of 9 organoids from the non-mutant group and 7 from the mutant group were used. The organoids were from three separate differentiation experiments. Each dot on the graph (Figure S1Q) represents a separate organoid.

Dilatations number and area

For the experiment performed using the CRISPR-edited hESC lines: One section for each of nine organoids, from three independent differentiation experiments was quantified. For dilatation number (Figure 3C), dilatations (areas that appeared empty under brightfield microscopy) in organoid sections

treated with FSK were counted manually. Each dot on the graph represents the count in a single organoid. For dilatation area as a percentage of the total area of the section (Figure 3D), each dilatation was encircled manually using Fiji-ImageJ and the area measured. The individual area values were summed, then divided by the total section area and expressed as a percentage. Each dot on the graph represents a separate organoid.

For the equivalent experiment using the patient-derive iPSC lines (Figures S2H, I), quantification was performed as above, with the exception that three organoids were quantified per group, each from an independent differentiation experiment.

Statistical Analysis

Information on statistics used in the analysis of each experiment can be also found in the figure legends and supplementary figure legends. Two-tailed t-tests were performed for the comparison – between non-mutant and mutant groups – of organoid size (Figure S1F), LTL+ and CDH1+ staining area (Figures 2H, I), and percentage of BrdU+ and active-Caspase3 cells (Figures S1N, Q). One-way ANOVA, followed by post-hoc t-tests was performed to analyse the results of the cAMP-induced tubule lumen dilatation experiment (Figures 3C, D and Figures S2H, I). Graphs for all of the above experiments present mean values, with error bars denoting standard error of the mean (S.E.M.). The dots represent individual data points and n-numbers count individual organoids.

SUPPLEMENTAL REFERENCES

Amezquita, R.A., Lun, A.T.L., Becht, E., Carey, V.J., Carpp, L.N., Geistlinger, L., Marini, F., Rue-Albrecht, K., Risso, D., Soneson, C., et al. (2020). Orchestrating single-cell analysis with Bioconductor. Nat Methods *17*, 137-145. 10.1038/s41592-019-0654-x.

Bolger, A.M., Lohse, M., and Usadel, B. (2014). Trimmomatic: a flexible trimmer for Illumina sequence data. Bioinformatics *30*, 2114-2120. 10.1093/bioinformatics/btu170.

Dobin, A., Davis, C.A., Schlesinger, F., Drenkow, J., Zaleski, C., Jha, S., Batut, P., Chaisson, M., and Gingeras, T.R. (2013). STAR: ultrafast universal RNA-seq aligner. Bioinformatics *29*, 15-21. 10.1093/bioinformatics/bts635.

Fabregat, A., Sidiropoulos, K., Garapati, P., Gillespie, M., Hausmann, K., Haw, R., Jassal, B., Jupe, S., Korninger, F., McKay, S., et al. (2016). The Reactome pathway Knowledgebase. Nucleic Acids Res *44*, D481-487. 10.1093/nar/gkv1351.

Gupta, S., Stamatoyannopoulos, J.A., Bailey, T.L., and Noble, W.S. (2007). Quantifying similarity between motifs. Genome Biol *8*, R24. 10.1186/gb-2007-8-2-r24.

Janky, R., Verfaillie, A., Imrichova, H., Van de Sande, B., Standaert, L., Christiaens, V., Hulselmans, G., Herten, K., Naval Sanchez, M., Potier, D., et al. (2014). iRegulon: from a gene list to a gene regulatory network using large motif and track collections. PLoS Comput Biol *10*, e1003731. 10.1371/journal.pcbi.1003731.

Kanehisa, M., Furumichi, M., Tanabe, M., Sato, Y., and Morishima, K. (2017). KEGG: new perspectives on genomes, pathways, diseases and drugs. Nucleic Acids Res *45*, D353-D361. 10.1093/nar/gkw1092.

Lopes, F.M., Kimber, S.J., and Bantounas, I. (2021). In situ Hybridization of miRNAs in Human Embryonic Kidney and Human Pluripotent Stem Cell-derived Kidney Organoids. Bio Protoc *11*, e4150. 10.21769/BioProtoc.4150.

Love, M.I., Huber, W., and Anders, S. (2014). Moderated estimation of fold change and dispersion for RNA-seq data with DESeq2. Genome Biol *15*, 550. 10.1186/s13059-014-0550-8.

Luo, W., and Brouwer, C. (2013). Pathview: an R/Bioconductor package for pathway-based data integration and visualization. Bioinformatics *29*, 1830-1831. 10.1093/bioinformatics/btt285.

Ye, J., Bates, N., Soteriou, D., Grady, L., Edmond, C., Ross, A., Kerby, A., Lewis, P.A., Adeniyi, T., Wright, R., et al. (2017). High quality clinical grade human embryonic stem cell lines derived from fresh discarded embryos. Stem Cell Res Ther *8*, 128. 10.1186/s13287-017-0561-y.

Yu, G., Wang, L.G., Han, Y., and He, Q.Y. (2012). clusterProfiler: an R package for comparing biological themes among gene clusters. Omics *16*, 284-287. 10.1089/omi.2011.0118.
